# Virtual Fragment Screening Identification of a Quinoline‐5,8‐dicarboxylic Acid Derivative as a Selective JMJD3 Inhibitor

**DOI:** 10.1002/cmdc.201800198

**Published:** 2018-05-22

**Authors:** Assunta Giordano, Federica del Gaudio, Catrine Johansson, Raffaele Riccio, Udo Oppermann, Simone Di Micco

**Affiliations:** ^1^ Institute of Biomolecular Chemistry (ICB) Consiglio Nazionale delle Ricerche (CNR) Via Campi Flegrei 34 80078 Pozzuoli (Napoli) Italy; ^2^ Department of Pharmacy University of Salerno Via Giovanni Paolo II, 132 84084 Fisciano (Salerno) Italy; ^3^ PhD Program in Drug Discovery and Development University of Salerno Via Giovanni Paolo II, 132 84084 Fisciano (Salerno) Italy; ^4^ Farmaceutici Damor S.p.A Via E. Scaglione 27 80145 Naples Italy; ^5^ Botnar Research Centre, Oxford NIHR BRU Oxford University, Oxford Centre for Translational Myeloma Research Oxford OX3 7LD UK; ^6^ Freiburg Institute for Advanced Studies (FRIAS) University of Freiburg Albertstraße 19 79104 Freiburg Germany

**Keywords:** anticancer agents, drug discovery, fragment-based approach, molecular modeling, selective JMJD3 inhibitor

## Abstract

The quinoline‐5,8 dicarboxylic acid scaffold has been identified by a fragment‐based approach as new potential lead compound for the development of JMJD3 inhibitors. Among them, 3‐(2,4‐dimethoxypyrimidin‐5‐yl)quinoline‐5,8‐dicarboxylic acid (compound **3**) shows low micromolar inhibitory activity against Jumonji domain‐containing protein 3 (JMJD3). The experimental evaluation of inhibitory activity against seven related isoforms of JMJD3 highlighted an unprecedented selectivity toward the biological target of interest.

Jumonji domain‐containing protein 3 (JMJD3), along with ubiquitously transcribed X chromosome tetratricopeptide repeat protein (UTX) constitutes the KDM6 subfamily, which catalyzes the demethylation of lysine 27 on histone H3 (H3K27). Both enzymes play key roles in the epigenetic regulation of gene expression, altering cellular memory, and reprogramming cellular fate. These proteins share a highly homologous Jumonji C domain endowed with Fe^2+^ and α‐ketoglutarate for the demethylation of H3K27.^1^ Overexpression of JMJD3 is correlated with inflammation,^2^, ^3^ neurological disorders,^4^ as well as cancer pathologies^5^ such as T‐cell acute lymphoblastic leukemia,^6^ Hodgkin's lymphoma,^7^ and metastatic prostate cancer.^8^ It has also been recently shown that JMJD3 can be a new target for pediatric brainstem glioma.^9^ Because JMJD3 is an inducible enzyme, its suppression could be very attractive for cancer treatment. Moreover, the specific biological function of JMJ enzymes in regulating cellular processes is still poorly understood due to the absence of selective inhibitors. Despite a vast body of work investigating the function of this protein in recent years, only one JMJD3/UTX binder (GSK‐J1) has been reported to date.^1^ Recently, a series of GSK‐J1 derivatives was reported, showing activity similar to or lower than that of the reference compound.^10^ Thus, the discovery of small molecules that are able to selectively modulate the biological function of JMJD3 is very attractive, in that they will shed light on its role, both in normal biological processes and under disease conditions, expanding the cancer therapy toolkit.

In detail, by using an in silico approach, we screened a fragment library of metal chelators (Supporting Information Figure S1) that was previously proposed to develop metalloprotein inhibitors.^11^, ^12^ As protein model, we used two available X‐ray structures of JMJD3 (PDB IDs https://www.rcsb.org/structure/4ASK as Model A and https://www.rcsb.org/structure/2XXZ as Model B), as structural experiments revealed different spatial rearrangements of some residues and of the Fe^2+^ ion upon GSK‐J1 binding.^1^ Following the same strategy adopted for our previous investigations of metalloproteins,^13^, ^14^, ^15^, ^16^, ^17^, ^18^ the charges of iron and its coordinating amino acids (H1390, E1392, and H1470) were refined by DFT calculations (see experimental details in the Supporting Information), and they were subsequently used for molecular docking calculations. Based on our analysis, we selected the quinoline‐8‐carboxylic acid fragment (B11, Figure S1, Supporting Information), which was advantageously accommodated into the α‐ketoglutarate cavity. With respect to the other fragments, its docked pose is deeply positioned, maximizing interactions with macromolecular counterparts. The docked pose suggested a modification at C8 to increase the interaction network with JMJD3; accordingly, we inserted a second carboxylic acid group to interact with K1381, T1387 and N1400. The docking pose of the modified fragment (B11′) showed the establishment of the foreseen interactions without affecting its global conformation. We also observed that the docked pose of B11′ orients the C3 position toward a small cavity adjacent to the α‐ketoglutarate pocket (Supporting Information Figure S2). Therefore, to identify additional possible interactions with this pocket by chemical decoration of the C3 position of B11′, we performed an AutoLigand^19^, ^20^ analysis. This investigation (Supporting Information Figure S2) suggested the advantageous placement of H‐bond donors/acceptors close to residues R1246 and N1331, as well as hydrophobic substituents to interact with other delimiting residues (F1328, T1330, T1387, and P1388). Thus, we designed a small library inserting chemically diverse aromatic substituents endowed with H‐bond acceptor/donors (Scheme 1 and Supporting Information Figure S3), and docked them on both protein conformations (Models A and B). Based on molecular docking energies and visual inspection, the docking outcomes of all tested compounds (**3**–**68**, Scheme 1 and Supporting Information Figure S3) led to a focused library of quinoline derivatives (**3**–**12**, Scheme 1), useful to provide information for structure–activity relationships.

**Scheme 1:**
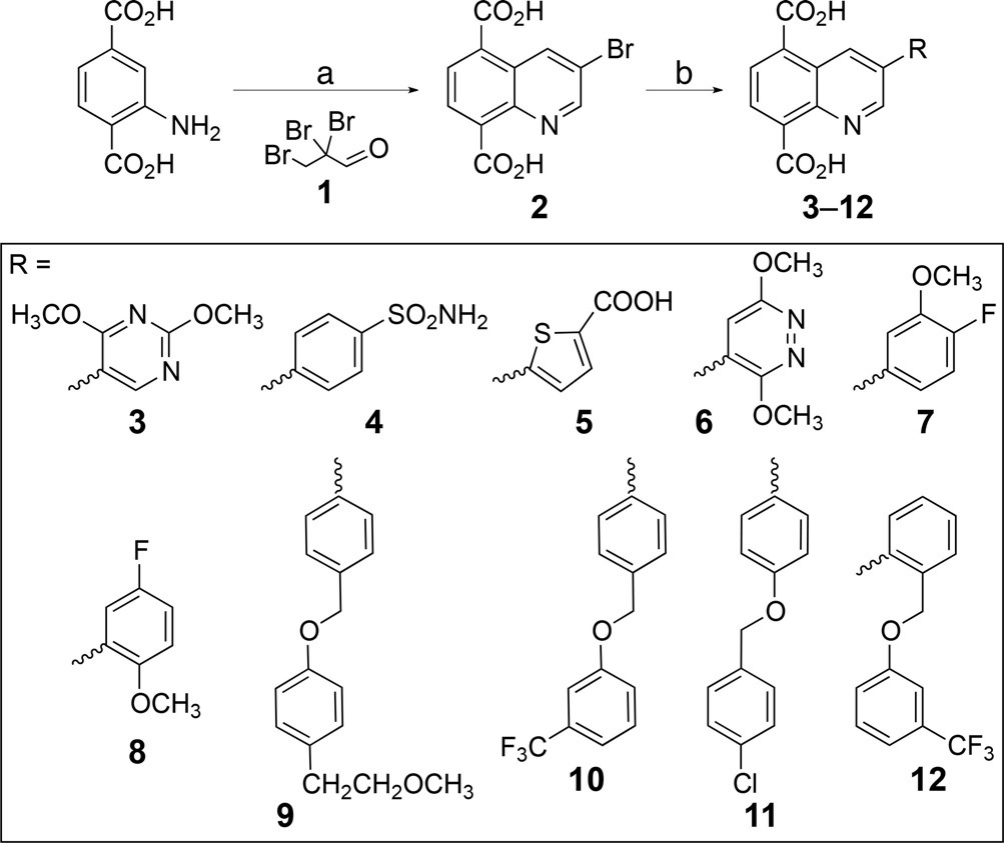
Structures and synthesis of compounds **3**–**12**: a) 2,2,3‐tribromopropanal **1**, AcOH, 110 °C; b) RB(OH)_2_, K_2_CO_3_, Pd(PPh_3_)_4_, 1,4‐dioxane/H_2_O, 80 °C. See experimental details in the Supporting Information.

The docked poses of compounds **3**–**12** into Model A highlighted the respect of a pattern of similar interactions by the common structural portion. Indeed, they coordinate the Fe^2+^ ion in a bidentate manner by the carboxylate group at C5, whereas the second carboxylic function establishes ionic interactions with K1381 and forms H‐bonds with T1387 and N1400. Notably, these interactions were observed in the co‐crystal structure with GSK‐J1.^1^ The quinoline ring forms π–π interactions with Y1379 and is H‐bonded to T1387. Compounds **3**–**12** differ in terms of the interaction given by the substituent at C3 (Scheme 1). Indeed, the methoxy group at C2 of the pyrimidine ring of **3** establishes H‐bonds with R1246 and N1331, and the nitrogen atom at position 1 is H‐bonded with N1331 (Figure 1 a).

An interaction is observed between the pyrimidine ring and N1246 (Figure 1 a). The second methoxy group establishes van der Waals interactions with the side chains of F1328, T1387, and P1388 (Figure 1 a). The sulfonamide and carboxylic acid groups of **4** and **5** (Supporting Information Figures S4 a, S5 a), respectively, accept two H‐bonds from R1246 and N1331. The pyridazine ring of **6** is H‐bonded to N1331 and establishes van der Waals contacts with F1328 and P1388 (Figure S6 a). Compound **7** accepts an H‐bond by methoxy group and fluorine from N1331 and R1246 (Figure S7 a). Compound **8** orthogonally interacts with N1331 by fluorine and establishes van der Waals interactions with F1328 and P1388 by a methoxy group (Figure S8 a). The linear chain oxygen atom of **9** accepts an H‐bond from R1246, and the 2‐methoxyethylphenyl moiety undergoes van der Waals interactions with P1388 and H1390 (Figure S9 a). The substituent at C3 of **10**–**12** establishes van der Waals contacts with F1328, T1387, P1388, H1390, and L1433 (Figures S10 a–12 a). Concerning the theoretical results on Model B, we observed that **3** is well accommodated into the binding pocket (Figure 1 b), keeping the same docked pose and interactions with protein residues found for the predicted conformation into Model A. In contrast, for compounds **4**–**12** (Figures S4 b–S12 b) we found only partial accommodation into the protein cavity, where some contacts with T1330 and F1328 are lost, especially for **9**–**12**, which are endowed with bulky substituents at C3 (Figures S9 b–S12 b). In greater detail, we observed that **4**–**12** coordinate the Fe^2+^ ion in a monodentate manner (Figures S4 b–S12 b). Although **4** and **5** establish π–cation interactions with R1246, they do not correctly orient the sulfonamide and carboxylic groups to interact with R1246, as observed for Model A (Figures S4 and S5). Compounds **6** and **7** are H‐bonded with R1246, by a pyridazine ring and methoxy group, respectively (Figures S6 and S7).

**Figure 1:**
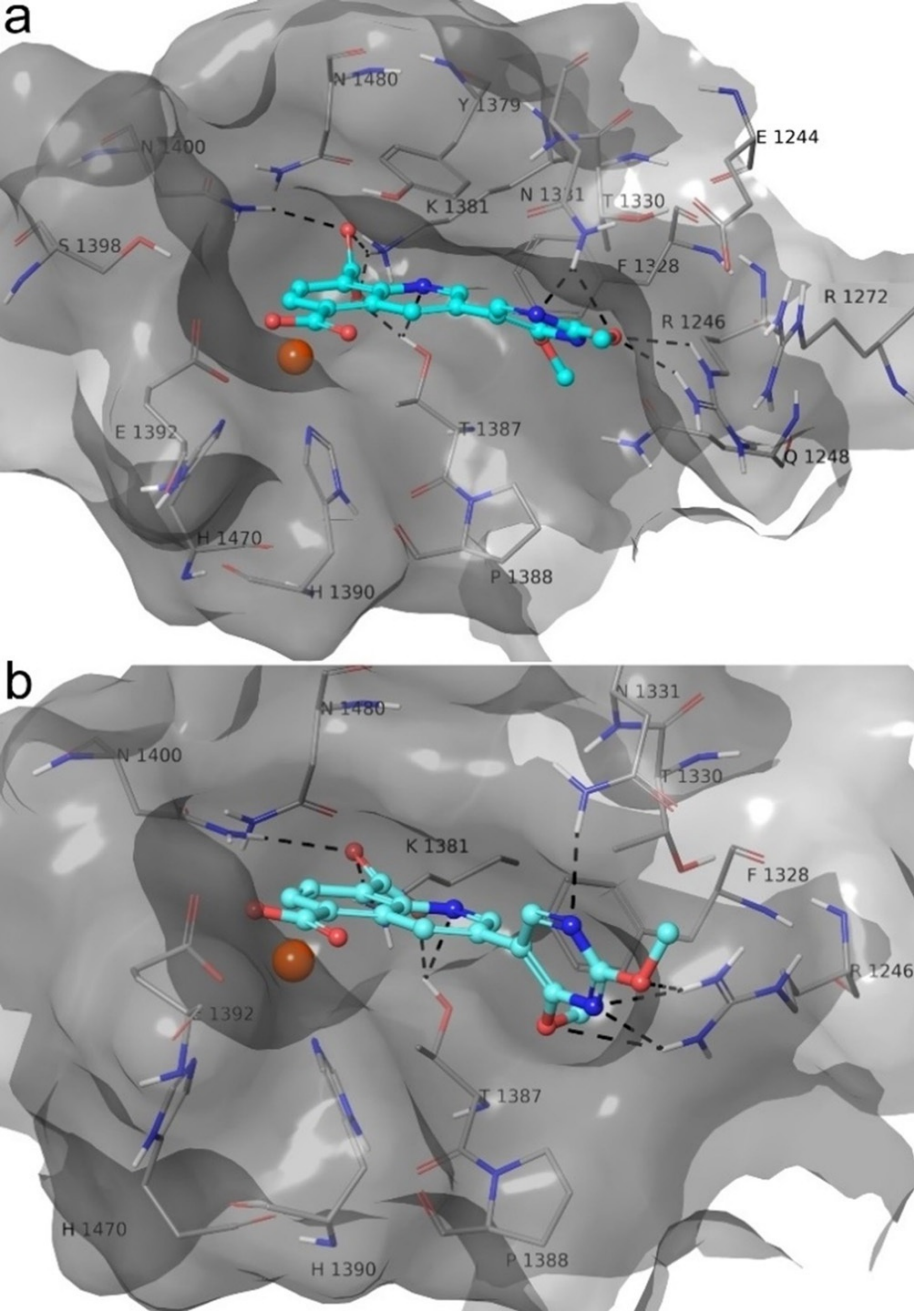
3D model of the interactions of a) **3**‐Model A and b) **3**‐Model B. JMJD3 is represented by molecular surface and tube, and **3** by sticks (cyan) and balls. The atom color codes are: C (**3**), cyan; C (JMJD3), grey; polar H, white; N, dark blue; O, red. The dashed black lines indicate the H‐bonds between ligand and protein.

To evaluate the stability of the complexes between JMJD3 and compounds **3**–**12** obtained by docking analysis, we performed molecular dynamics simulations (50 ns, 310 K; see experimental details in the Supporting Information).^21^, ^22^ The trajectory analysis revealed that **3** gives a high number of contacts with protein residues, and it maintains most of the contacts observed from the docked pose during the entire simulation (>50 %) with both protein models (Figure 2, bottom). The heavy‐atom‐positional RMSD of **3** shows high stability during molecular dynamics simulations with respect to the protein backbone, with similar behavior on both protein models (Figure 2, top). The atom‐relative orientation of **3** is kept during the simulation with Models A and B. The trajectory of **4** and **6** bound to Model A was found to be stable, but with a larger RMSD than **3**‐Model A (Figures S13, S15), whereas the remaining compounds showed large fluctuations during the simulation (Figures S14, S16–S21). Large deviations from their initial positions were observed for **4**–**12**‐Model B complexes (Figures S13–S21). Compounds **4**–**12** bound to both models give a lower number of contacts during the simulations relative to **3** (Figures S13–S21). Overall, the comparison of docking results on both models, integrated by molecular dynamics, suggested higher activity of compound **3** with respect to **4**–**12** as experimentally confirmed (see below).

**Figure 2:**
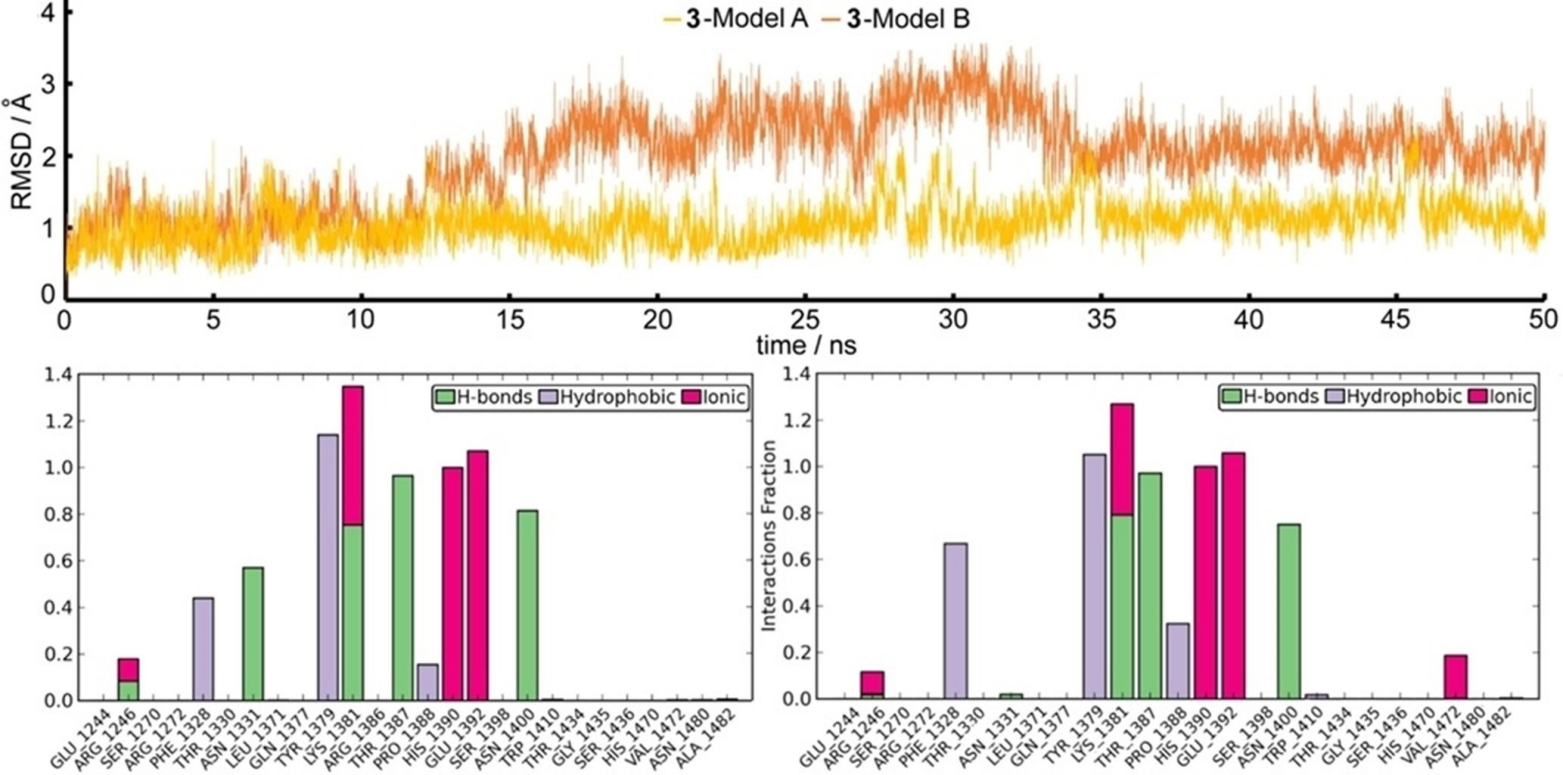
Top: Heavy atom‐positional RMSD (Å) of **3**‐Model A (yellow line) and **3**‐Model B (orange line) as function of simulation time (ns). Bottom: Protein–ligand contact histograms during the simulation of **3**‐Model A (left) and **3**‐Model B (right).

A modification of the Skraup reaction was used to synthesize the core structure (Scheme 1).^23^ 2,2,3‐Tribromopropanal **1** was prepared by bromination of acrolein with two moles of bromine in dichloroethane (see experimental details in the Supporting Information). Addition of the brominated aldehyde to aminoterephtalic acid in glacial acetic acid at 110 °C afforded 3‐bromo‐5,8‐dicarboxy‐quinoline **2**. The quinoline ring was further functionalized at position **3** by Suzuki–Miyaura coupling reaction^24^ with proper boronic acids under standard conditions with Pd(PPh_3_)_4_ as catalyst to give **3**–**12** (Scheme 1). Products **3**–**12** were obtained in moderate to high yields (53–75 %). Reactions proceeded in lower yields with electron‐deficient heterocyclic boronic acids, which are known to be susceptible to protodeboronation.^25^

The synthesized compounds **3**–**12** were investigated for inhibition of JMJD3 (at 25 μm) by using AlphaScreen (Figure 3; see experimental details in the Supporting Information). The experiments revealed that **3** has the highest inhibitory activity (90 %). Compounds **4** and **5** also appreciably decrease enzyme activity by 65 % and 60 %, respectively. Lower enzyme modulation was observed for **6** (≈25 %) and **7** (≈12 %), whereas the remaining analogues **8**–**12** presented very low or no inhibitory activity. Notably, these experimental data are in good qualitative agreement with the theoretical outcomes, highlighting the structural features of substituents at C3 as responsible for the binding affinity for the macromolecule. We proceeded to investigate the inhibitory activity of **3** further by evaluation of its IC_50_ value against JMJD3. The results highlighted a low micromolar activity of compound **3** against JMJD3 (Table 1). Based on this result, we evaluated the selectivity of compound **3** versus seven highly structural related isoforms of our macromolecular target. Interestingly, unlike GSK‐J1,^1^ no significant inhibition (at 10 μm) was observed against UTX. Similar outcomes were obtained for **3** versus JMJD2C and JMJD2D (Table 1). Compound **3** weakly inhibits JMJD1A and FBXL11: 12 % and 15 % at 10 μm (Table 1), respectively. Relative to JMJD1A and FBXL11, we found a decrease in enzymatic activity (Table 1) toward Jarid1A and Jarid1B of 33 % and 26 % by **3**.

**Figure 3:**
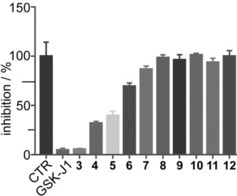
Effect of compounds **3**–**12** (25 μm) and GSK‐J1 (10 μm, reference compound) on JMJD3 activity. Data are given as the means±SEM, *n*=3.

**Table 1:** IC_50_±SEM and percent inhibition of eight isoforms by compound **3**.

Protein	IC_50_ [μm] or Percent Inhibition
JMJD3	1.52±0.50
UTX	NI^[a]^
JMJD2C	NI^[a]^
JMJD2D	NI^[a]^
JMJD1A	12 %^[b]^
FBXL11	15 %^[b]^
Jarid1A	33 %^[b]^
Jarid1B	26 %^[b]^

[a] No significant inhibition at 10 μm. [b] IC_50_>10 μm; the percentage inhibition at 10 μm was calculated.

In conclusion, we have identified the novel quinoline‐5,8 dicarboxylic acid scaffold by a fragment‐based approach to develop selective JMJD3 inhibitors. This unprecedented result affords the possibility of shedding light on the role of this enzyme in normal and altered tissues related to pathological events, such as cancer and inflammation. Moreover, the obtained outcomes validate our in silico strategy based on molecular docking studies against two JMJD3 conformations integrated by molecular dynamics simulations, which allows the identification of potential binders from virtual screening. These encouraging results prompt us to further explore other chelating fragments to design new and potent JMJD3 ligands for safer cancer treatment, as well as therapies for inflammation and neurological disorders.

## Experimental Section

Full experimental details are provided in the Supporting Information.

## Conflict of interest

*The authors declare no conflict of interest*.

## Supporting information

As a service to our authors and readers, this journal provides supporting information supplied by the authors. Such materials are peer reviewed and may be re‐organized for online delivery, but are not copy‐edited or typeset. Technical support issues arising from supporting information (other than missing files) should be addressed to the authors.

Supplementary

## References

[1] L. Kruidenier , C. W. Chung , Z. Cheng , J. Liddle , K. Che , G. Joberty , M. Bantscheff , C. Bountra , A. Bridges , H. Diallo , D. Eberhard , S. Hutchinson , E. Jones , R. Katso , M. Leveridge , P. K. Mander , J. Mosley , C. Ramirez-Molina , P. Rowland , C. J. Schofield , R. J. Sheppard , J. E. Smith , C. Swales , R. Tanner , P. Thomas , A. Tumber , G. Drewes , U. Oppermann , D. J. Patel , K. Lee , D. M. Wilson , Nature 2012, 488, 404–408. 22842901 10.1038/nature11262 PMC4691848

[2] F. De Santa , V. Narang , Z. H. Yap , B. K. Tusi , T. Burgold , L. Austenaa , G. Bucci , M. Caganova , S. Notarbartolo , S. Casola , G. Testa , W. K. Sung , C. L. Wei , G. Natoli , EMBO J. 2009, 28, 3341–3352. 19779457 10.1038/emboj.2009.271 PMC2752025

[3] F. De Santa , M. G. Totaro , E. Prosperini , S. Notarbartolo , G. Testa , G. Natoli , Cell 2007, 130, 1083–1094. 17825402 10.1016/j.cell.2007.08.019

[4] B. Yao , P. Jin , Genes Dev. 2014, 28, 1253–1271. 24939932 10.1101/gad.241547.114 PMC4066397

[5] M. Barradas , E. Anderton , J. C. Acosta , S. Li , A. Banito , M. Rodriguez-Niedenfuhr , G. Maertens , M. Banck , M. M. Zhou , M. J. Walsh , G. Peters , J. Gil , Genes Dev. 2009, 23, 1177–1182. 19451218 10.1101/gad.511109 PMC2685533

[6] P. Ntziachristos , A. Tsirigos , G. G. Welstead , T. Trimarchi , S. Bakogianni , L. Xu , E. Loizou , L. Holmfeldt , A. Strikoudis , B. King , J. Mullenders , J. Becksfort , J. Nedjic , E. Paietta , M. S. Tallman , J. M. Rowe , G. Tonon , T. Satoh , L. Kruidenier , R. Prinjha , S. Akira , P. Van Vlierberghe , A. A. Ferrando , R. Jaenisch , C. G. Mullighan , I. Aifantis , Nature 2014, 514, 513–517. 25132549 10.1038/nature13605 PMC4209203

[7] J. A. Anderton , S. Bose , M. Vockerodt , K. Vrzalikova , W. Wei , M. Kuo , K. Helin , J. Christensen , M. Rowe , P. G. Murray , C. B. Woodman , Oncogene 2011, 30, 2037–2043. 21242977 10.1038/onc.2010.579

[8] Y. Xiang , Z. Zhu , G. Han , H. Lin , L. Xu , C. D. Chen , Cell Res. 2007, 17, 850–857. 17923864 10.1038/cr.2007.83

[9] R. Hashizume , N. Andor , Y. Ihara , R. Lerner , H. Gan , X. Chen , D. Fang , X. Huang , M. W. Tom , V. Ngo , D. Solomon , S. Mueller , P. L. Paris , Z. Zhang , C. Petritsch , N. Gupta , T. A. Waldman , C. D. James , Nat. Med. 2014, 20, 1394–1396. 25401693 10.1038/nm.3716 PMC4257862

[10] J. Hu , X. Wang , L. Chen , M. Huang , W. Tang , J. Zuo , Y.-C. Liu , Z. Shi , R. Liu , S. Jingkang , B. Xiong , Bioorg. Med. Chem. Lett. 2016, 26, 721–725. 26776360 10.1016/j.bmcl.2016.01.006

[11] A. Agrawal , S. L. Johnson , J. A. Jacobsen , M. T. Miller , L. H. Chen , M. Pellecchia , S. M. Cohen , ChemMedChem 2010, 5, 195–199. 20058293 10.1002/cmdc.200900516 PMC2825879

[12] J. A. Jacobsen , J. L. Fullagar , M. T. Miller , S. M. Cohen , J. Med. Chem. 2011, 54, 591–602. 21189019 10.1021/jm101266s PMC3024453

[13] N. Maulucci , M. G. Chini , S. Di Micco , I. Izzo , E. Cafaro , A. Russo , P. Gallinari , C. Paolini , M. C. Nardi , A. Casapullo , R. Riccio , G. Bifulco , F. De Riccardis , J. Am. Chem. Soc. 2007, 129, 3007–3012. 17311384 10.1021/ja0686256

[14] S. Di Micco , S. Terracciano , I. Bruno , M. Rodriquez , R. Riccio , M. Taddei , G. Bifulco , Bioorg. Med. Chem. 2008, 16, 8635–8642. 18715788 10.1016/j.bmc.2008.08.003

[15] A. A. Grolla , V. Podesta , M. G. Chini , S. Di Micco , A. Vallario , A. A. Genazzani , P. L. Canonico , G. Bifulco , G. C. Tron , G. Sorba , T. Pirali , J. Med. Chem. 2009, 52, 2776–2785. 19344175 10.1021/jm801529c

[16] T. Pirali , V. Faccio , R. Mossetti , A. A. Grolla , S. Di Micco , G. Bifulco , A. A. Genazzani , G. C. Tron , Mol. Diversity 2010, 14, 109–121. 10.1007/s11030-009-9153-9 19475493

[17] S. Terracciano , S. Di Micco , G. Bifulco , P. Gallinari , R. Riccio , I. Bruno , Bioorg. Med. Chem. 2010, 18, 3252–3260. 20381359 10.1016/j.bmc.2010.03.022

[18] S. Di Micco , M. G. Chini , S. Terracciano , I. Bruno , R. Riccio , G. Bifulco , Bioorg. Med. Chem. 2013, 21, 3795–3807. 23693069 10.1016/j.bmc.2013.04.036

[19] R. Harris , A. J. Olson , D. S. Goodsell , Proteins Struct. Funct. Bioinf. 2007, 70, 1506–1517. 10.1002/prot.21645 17910060

[20] S. Forli , R. Huey , M. E. Pique , M. F. Sanner , D. S. Goodsell , A. J. Olson , Nat. Protoc. 2016, 11, 905–919. 27077332 10.1038/nprot.2016.051 PMC4868550

[21] S. Di Micco , B. Renga , A. Carino , M. V. D′Auria , A. Zampella , R. Riccio , S. Fiorucci , G. Bifulco , Steroids 2014, 80, 51–63. 24315836 10.1016/j.steroids.2013.11.017

[22] M. G. Chini , N. Malafronte , M. C. Vaccaro , M. J. Gualtieri , A. Vassallo , M. Vasaturo , S. Castellano , C. Milite , A. Leone , G. Bifulco , N. De Tommasi , F. Dal Piaz , Chem. Eur. J. 2016, 22, 13236–13250. 27492719 10.1002/chem.201602242

[23] C. Lamberth , F. Murphy Kessabi , R. Beaudegnies , L. Quaranta , S. Trah , G. Berthon , F. Cederbaum , T. Vettiger , C. S. Prasanna , Synlett 2014, 25, 858–862.

[24] I. Maluenda , O. Navarro , Molecules 2015, 20, 7528–7557. 25919276 10.3390/molecules20057528 PMC6272665

[25] K. M. Clapham , A. S. Batsanov , R. D. R. Greenwood , M. R. Bryce , A. E. Smith , B. Tarbit , J. Org. Chem. 2008, 73, 2176–2181. 18294000 10.1021/jo702420q

