# Supplementary material for: Virtual Fragment Screening Identification of a Quinoline‐5,8‐dicarboxylic Acid Derivative as a Selective JMJD3 Inhibitor
Source: ChemMedChem. 2018 May 22;13(12):1160–4. doi: 10.1002/cmdc.201800198 (PMC6055880; doi:10.1002/cmdc.201800198)
Supplement: Supplementary file 1 — Supplementary [file CMDC-13-1160-s001.pdf]

CHEMMEDCHEM
Chemistry & Drug Discovery

Supporting Information

Virtual Fragment Screening Identification of a Quinoline-5,8-dicarboxylic Acid Derivative as a Selective JMJD3 Inhibitor

Assunta Giordano,[a, b] Federica del Gaudio,[b, c, d] Catrine Johansson,[e] Raffaele Riccio,[b] Udo Oppermann,[e, f] and Simone Di Micco*[b]

cmdc_201800198_sm_miscellaneous_information.pdf

Supporting information

Figure S1. Molecular structures of chelator fragments. S3
Figure S2. Autoligand analysis of Model Aand Model B complexed with B11e. S4
Figure S3. Molecular structures of cmpounds **13-68**……………………….…................ S5
Figure S4. 3D model of the interactions between **4** and the Model A and Model B. S6
Figure S5. 3D model of the interactions between **5** and the Model A and Model B. S7
Figure S6. 3D model of the interactions between **6** and the Model A and Model B. S8
Figure S7. 3D model of the interactions between **7** and the Model A and Model B. S9
Figure S8. 3D model of the interactions between **8** and the Model A and Model B. S10
Figure S9. 3D model of the interactions between **9** and the Model A and Model B. S11
Figure S10. 3D model of the interactions between **10** and the Model A and Model B. .. S12
Figure S11. 3D model of the interactions between **11** and the Model A and Model B. .. S13
Figure S12. 3D model of the interactions between **12** and the Model A and Model B. .. S14
Figure S13. (a) Heavy atom-positional RMSD (Å) of **4** bound to Model A and Model B. (b) Protein-ligand contacts histograms during the simulation of **4**-Model A and **4**-Model B. S15
Figure S14. (a) Heavy atom-positional RMSD (Å) of **5** bound to Model A and Model B. (b) Protein-ligand contacts histograms during the simulation of **5**-Model A and **5**-Model B. S16
Figure S15. (a) Heavy atom-positional RMSD (Å) of **6** bound to Model Aand Model B. (b) Protein-ligand contacts histograms during the simulation of **6**-Model A and **6**-Model B. S17
Figure S 16. (a) Heavy atom-positional RMSD (Å) of **7** bound to Model A and Model B. (b) Protein-ligand contacts histograms during the simulation of **7**-Model A and **7**-Model B. S18
Figure S17. (a) Heavy atom-positional RMSD (Å) of **8** bound to Model A and Model B. (b) Protein-ligand contacts histograms during the simulation of **8**-Model A and **8**-Model B. S19
Figure S18. (a) Heavy atom-positional RMSD (Å) of **9** bound to Model A and Model B. (b) Protein-ligand contacts histograms during the simulation of **9**-Model A and **9**-Model B. S20
Figure S19. (a) Heavy atom-positional RMSD (Å) of **10** bound to Model A and Model B. (b) Protein-ligand contacts histograms during the simulation of **10**-Model A and **10**-Model B. S21
Figure S20. (a) Heavy atom-positional RMSD (Å) of **11**-Model A and **11**-Model B. (b) Protein-ligand contacts histograms during the simulation of **11**-Model A and **11**-Model B. S22
Figure S21. (a) Heavy atom-positional RMSD (Å) of **12**-Model A and **12**-Model B. (b) Protein-ligand contacts histograms during the simulation of **12**-Model A and **12**-Model B. S23
Experimental details…………………………………………………………………………S24
Figure S22. Superimposition of co-crystallized and docked poses of GSK-J1. .... S25
Figure S23. Compound **2** ^{1}H NMR (CD_3OD, 300 MHz). S35
Figure S24. Compound **3** ^{1}H NMR (DMSO, 600 MHz). S36
Figure S25. Compound **3** HSQC (DMSO, 600 MHz). S37
Figure S26. Compound **3** HMBC (DMSO, 600 MHz). S38
Figure S27. Compound **4** ^{1}H NMR ($CDCl_3$/CD_3OD 1:1, 300 MHz). S39

Figure S28. Compound **5** ^{1}H NMR (DMSO, 500 MHz).. S40
Figure S29. Compound **6** ^{1}H NMR (DMSO, 300 MHz).. S41
Figure S30. Compound **7** ^{1}H NMR (DMSO, 300 MHz).. S42
Figure S31. Compound **8** ^{1}H NMR ($CDCl_3$/CD_3OD 1:1, 300 MHz)................................. S43
Figure S32. Compound **9** ^{1}H NMR (DMSO, 300 MHz).. S44
Figure S33. Compound **10** ^{1}H NMR (DMSO, 300 MHz).. S45
Figure S34. Compound **11** ^{1}H NMR (DMSO, 300 MHz).. S46
Figure S35. Compound **12** ^{1}H NMR (DMSO, 300 MHz).. S47
Figure S36. Compound **2** ESI-MS... S48
Figure S37. Compound **3** HRMS S49
Figure S38. Compound **4** HRMS S50
Figure S39. Compound **5** ESI-MS... S51
Figure S40. Compound **6** ESI-MS... S52
Figure S41. Compound **7** ESI-MS... S53
Figure S42. Compound **8** ESI-MS... S54
Figure S43. Compound **9** ESI-MS... S55
Figure S44. Compound **10** ESI-MS... S56
Figure S45. Compound **11** ESI-MS... S57
Figure S46. Compound **12** ESI-MS... S58
References…………………………………………………………………………… S59

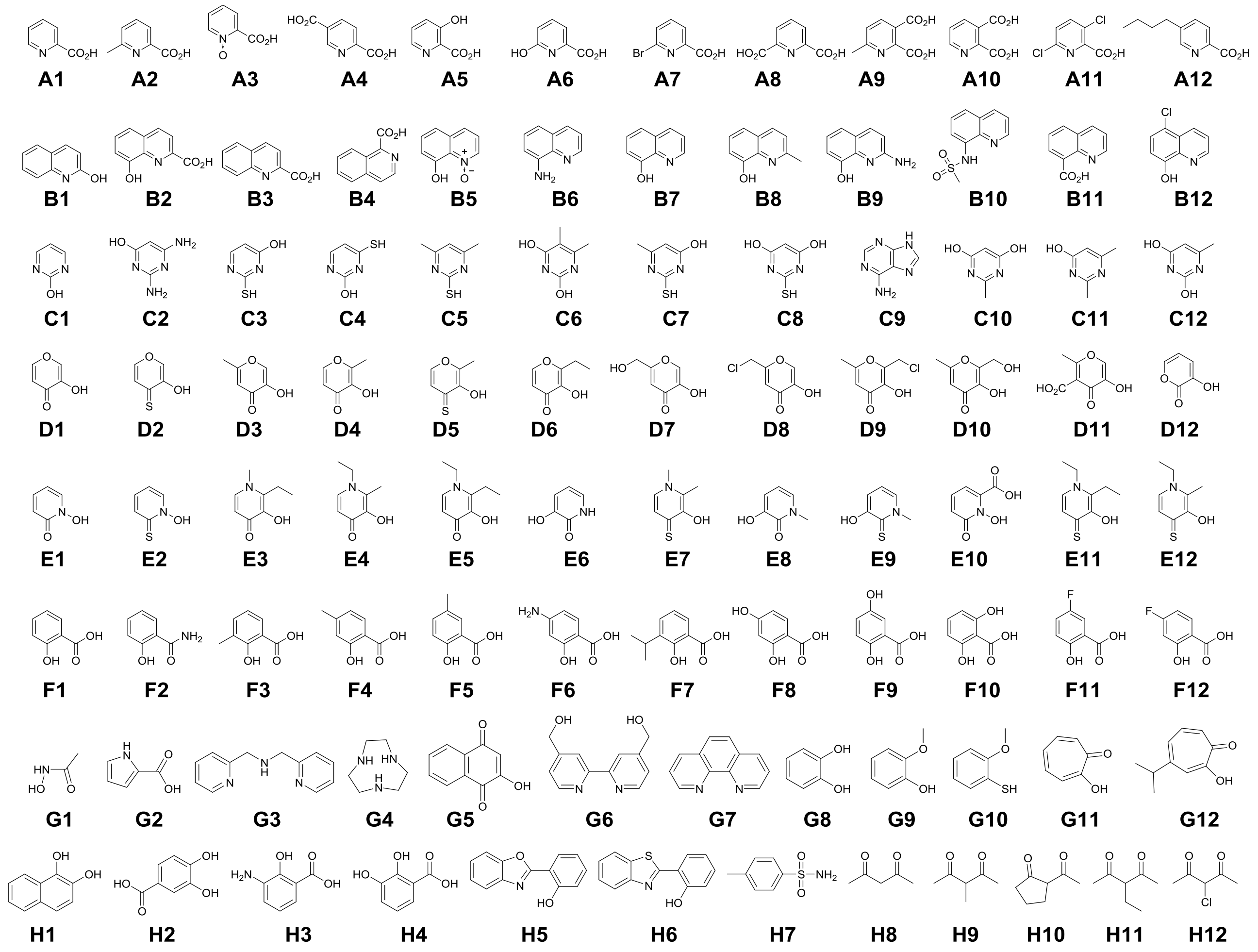

Figure S1. Molecular structures of chelator fragments.

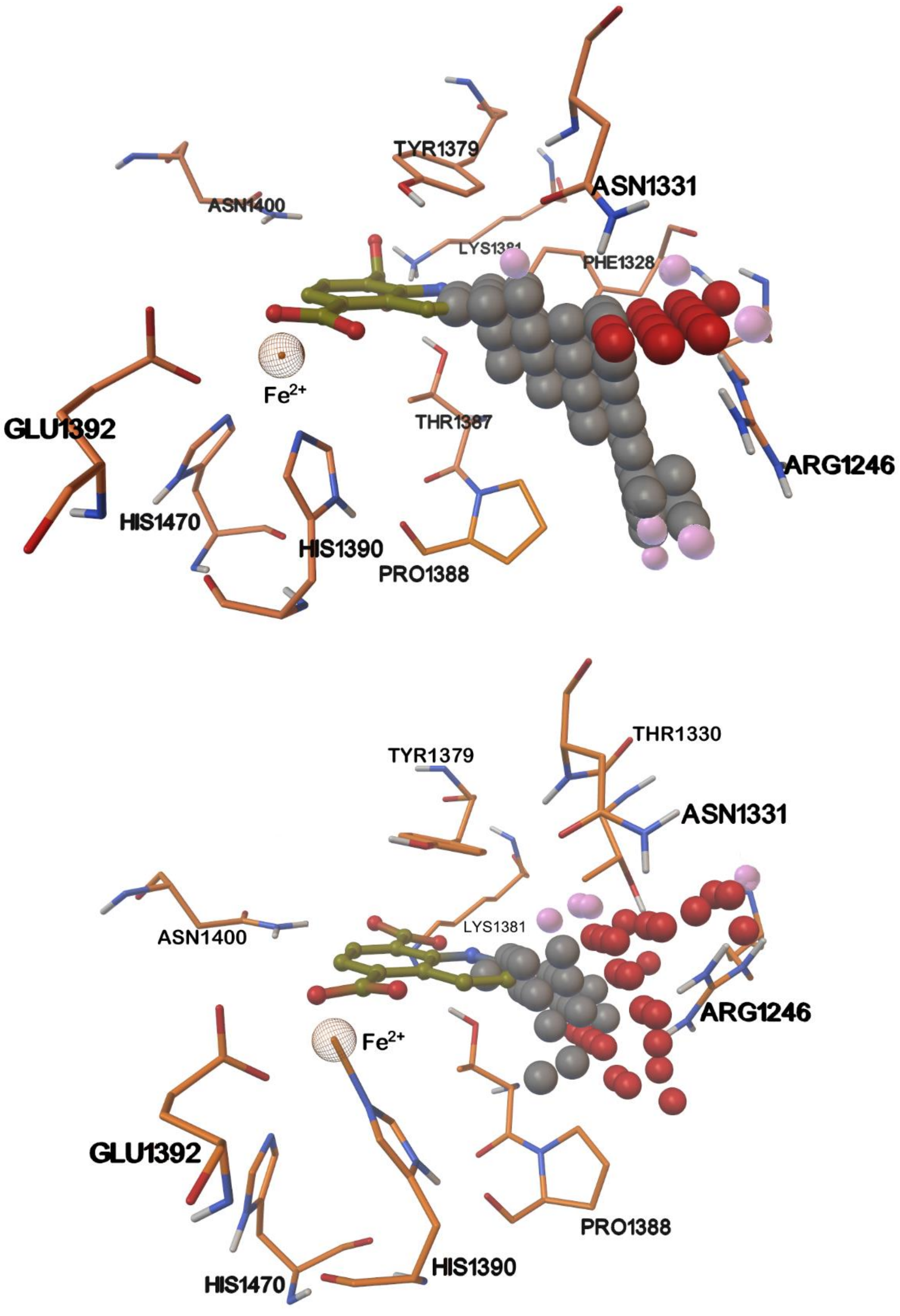

Figure S2. Model A (top, PDB ID: 4ASK) and Model B (bottom, PDB ID: 2XXZ) complexed with B11e (in green) and the AutoLigand generated best affinity fill volume (70 points) colored: H-bond donor, pink; H-bond acceptor, red; hydrophobic group, grey. The fragment is depicted in sticks (kaki) and balls. Protein residues and $Fe^{2+}$are represented in tube and orange cpk, respectively. The atoms are colored: polar H, white; N, dark-blue; O, red; protein C atom, orange.

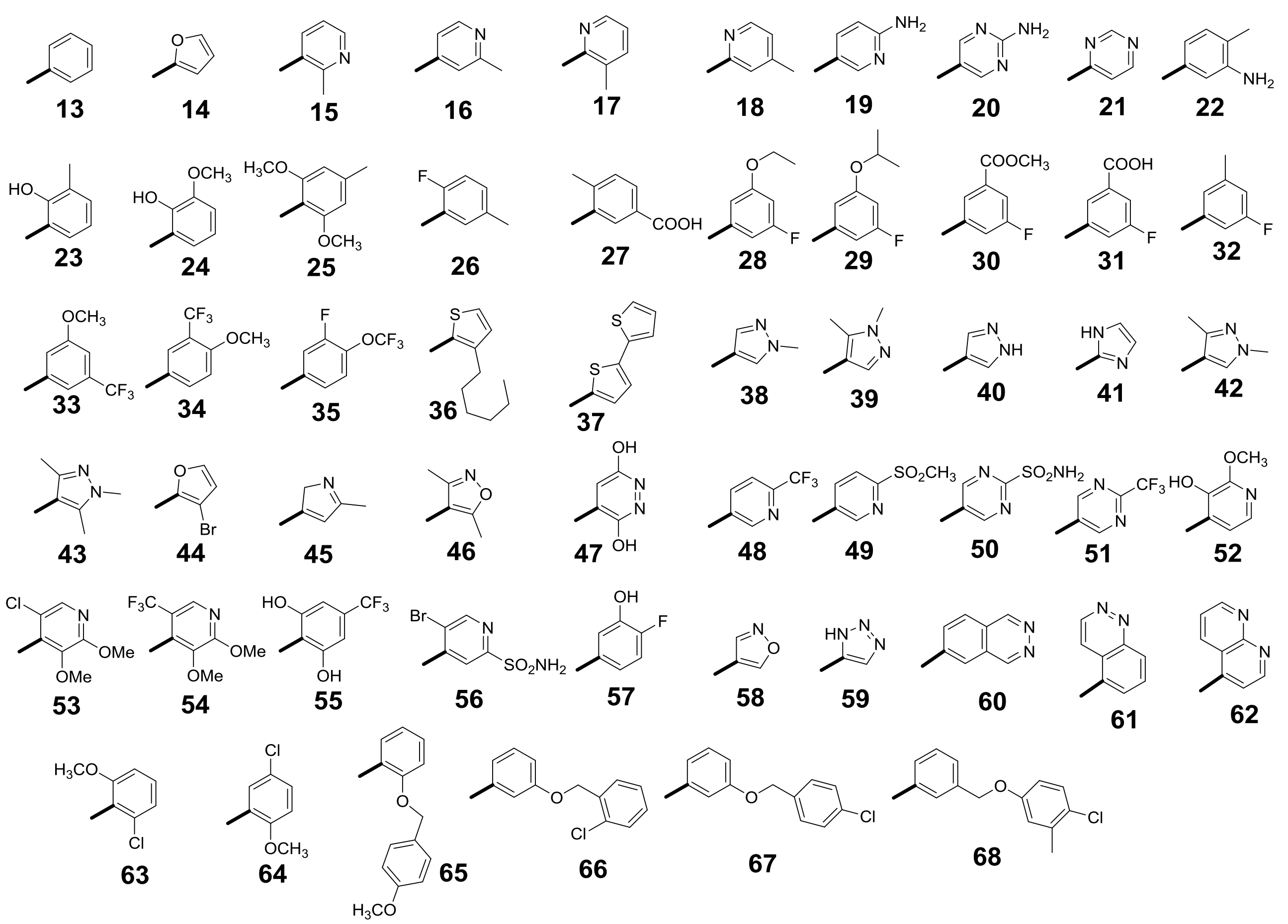

Figure S3. Molecular structures of substituents at C3 of **2** to obatin compounds **13-68**.

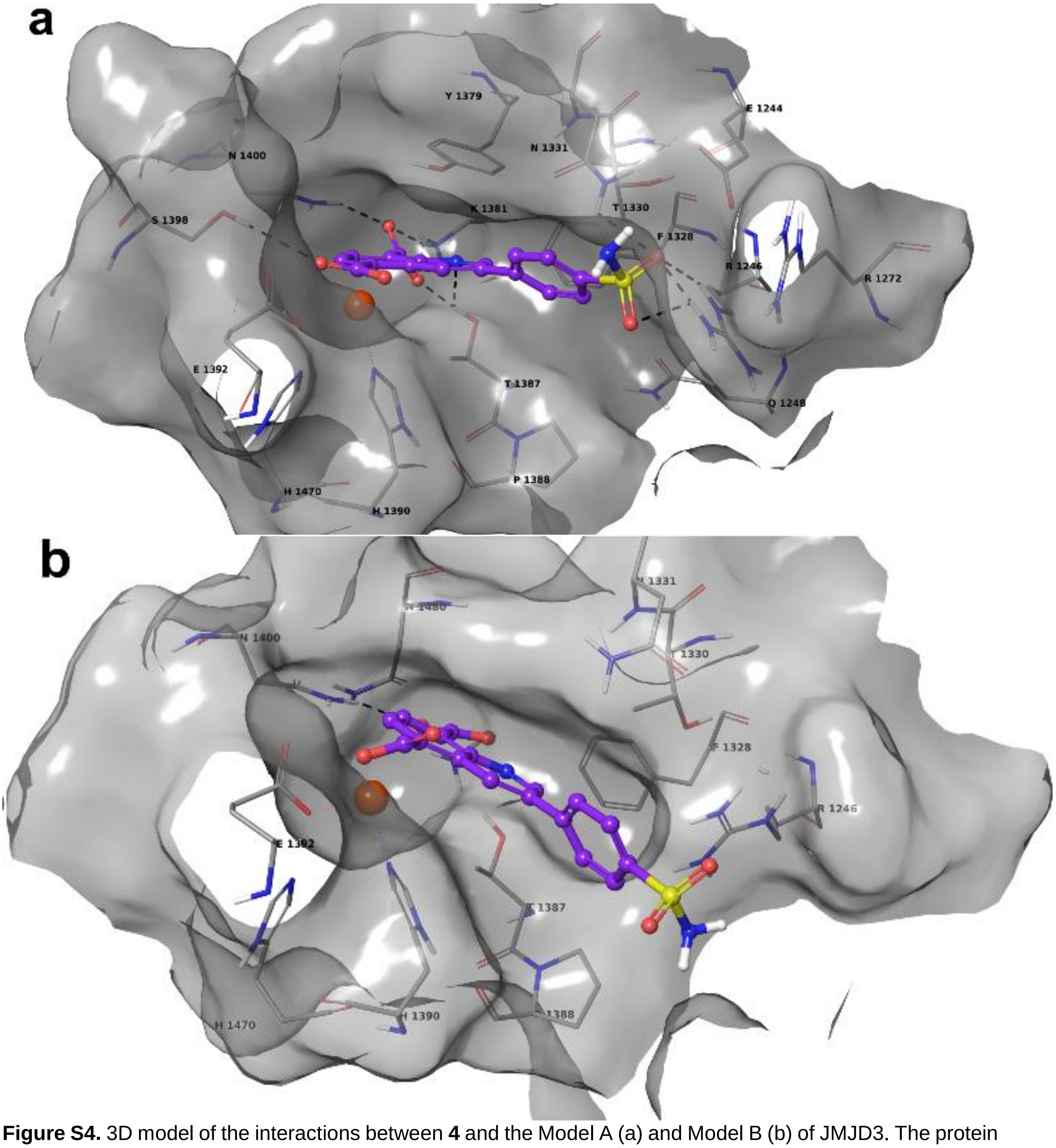

Figure S4. 3D model of the interactions between **4** and the Model A (a) and Model B (b) of JMJD3. The protein is represented by molecular surface and tube. **4** is depicted by sticks (violet) and balls. The atom color codes are: C (**4**), violet; C (JMJD3), grey; polar H, sky blue; N, dark blue; O, red. The dashed black lines indicate the hydrogen bonds between ligand and protein.

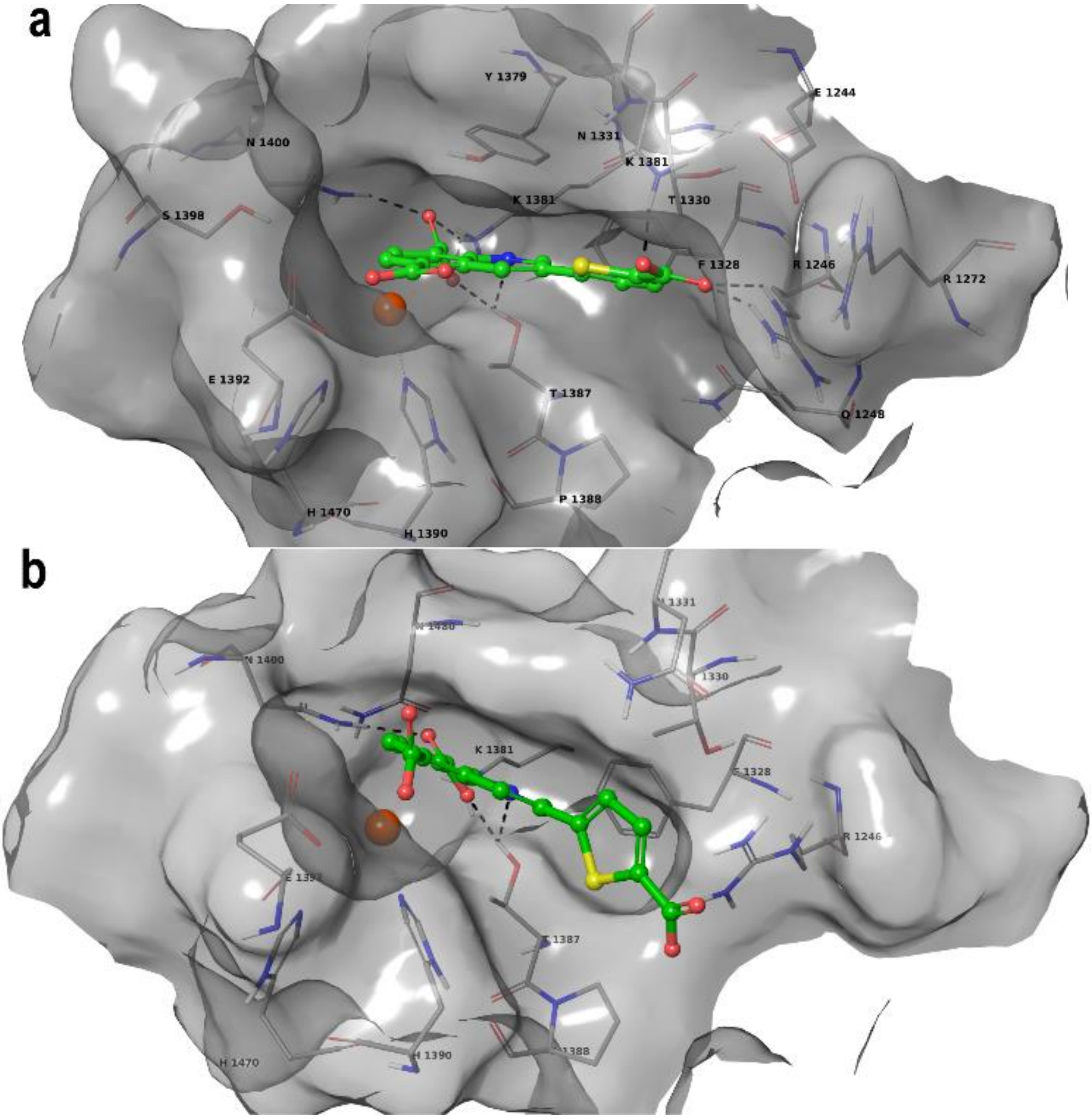

Figure S5. 3D model of the interactions between **5** and the Model A (a) and Model B (b) of JMJD3. The protein is represented by molecular surface and tube. **5** is depicted by sticks (green) and balls. The atom color codes are: C (**5**), green; C (JMJD3), grey; polar H, sky blue; N, dark blue; O, red. The dashed black lines indicate the hydrogen bonds between ligand and protein.

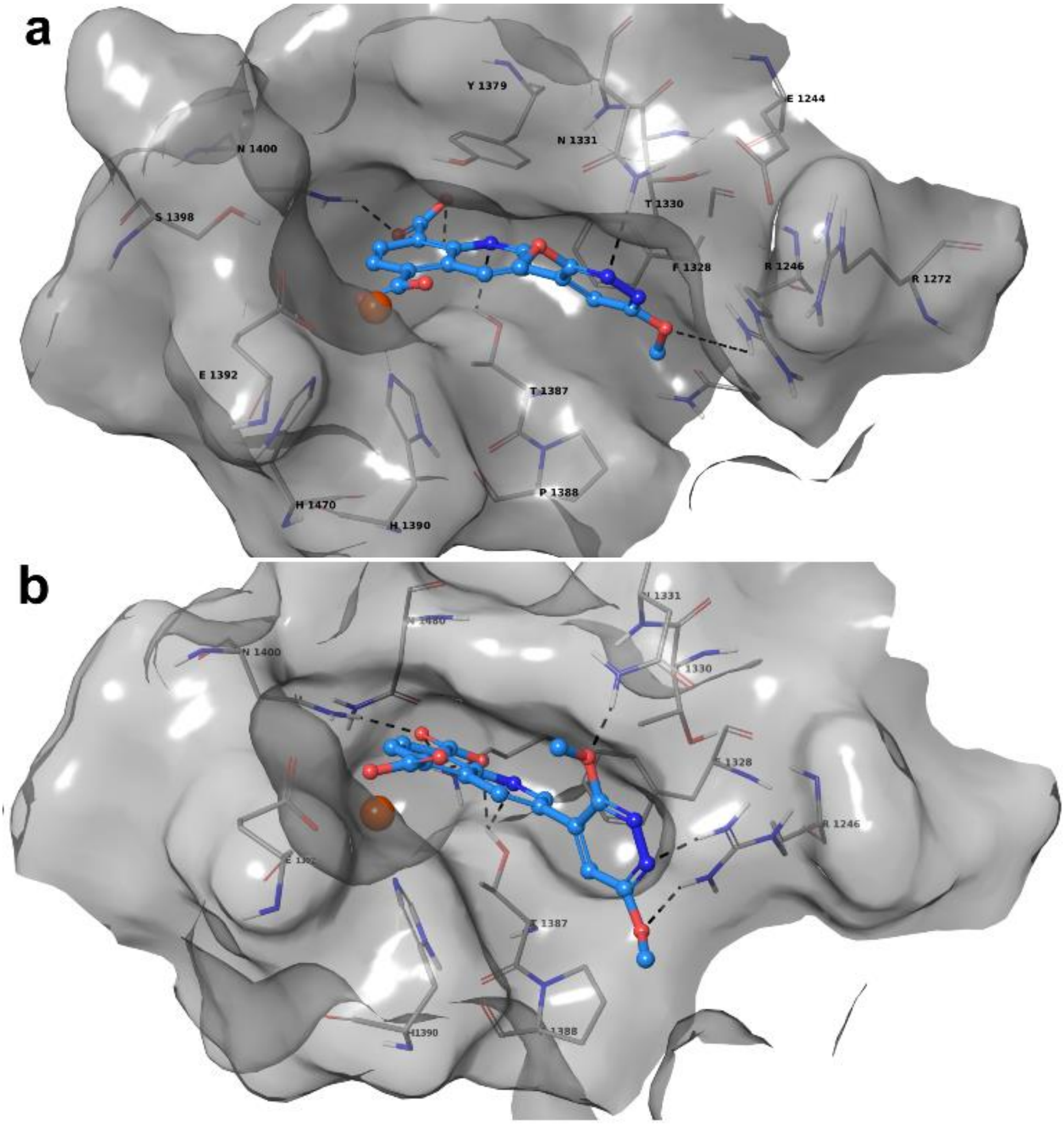

Figure S6. 3D model of the interactions between **6** and the Model A (a) and Model B (b) of JMJD3. The protein is represented by molecular surface and tube. **6** is depicted by sticks (blue) and balls. The atom color codes are: C (**6**), blue; C (JMJD3), grey; polar H, sky blue; N, dark blue; O, red. The dashed black lines indicate the hydrogen bonds between ligand and protein.

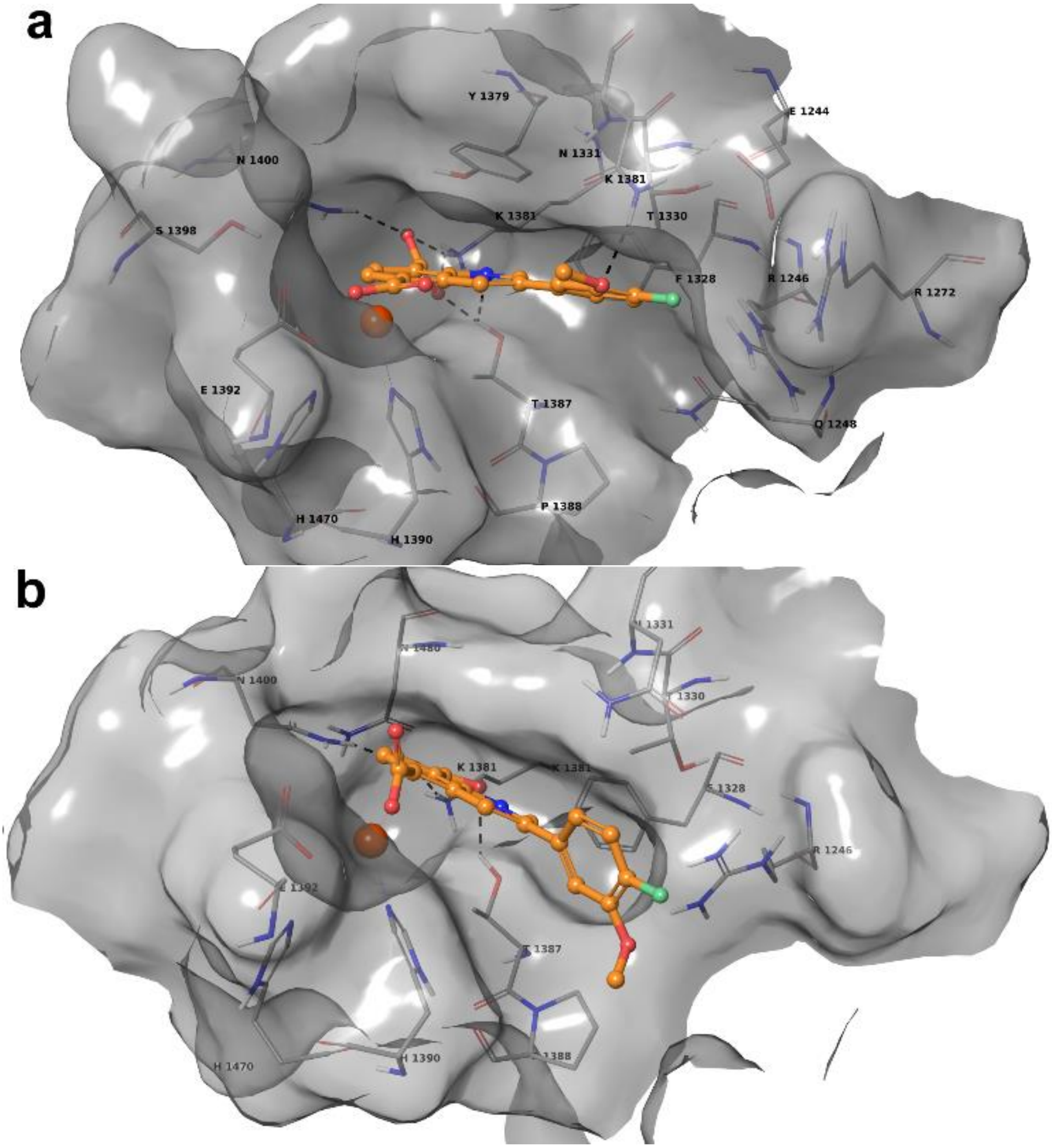

Figure S7. 3D model of the interactions between **7** and the Model A (a) and Model B (b) of JMJD3. The protein is represented by molecular surface and tube. **7** is depicted by sticks (orange) and balls. The atom color codes are: C (**7**), orange; C (JMJD3), grey; polar H, sky blue; N, dark blue; O, red. The dashed black lines indicate the hydrogen bonds between ligand and protein.

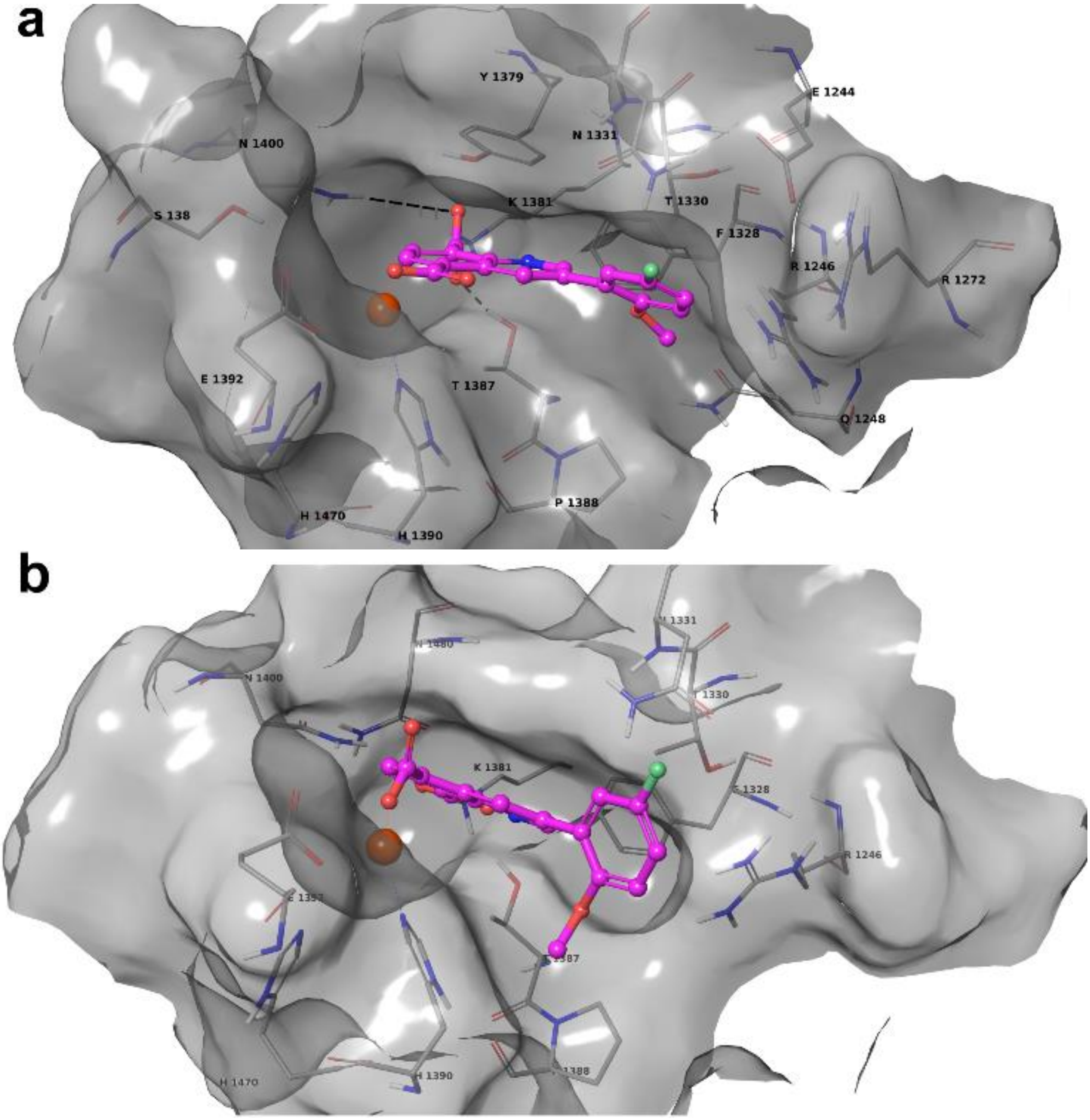

Figure S8. 3D model of the interactions between **8** and the Model A (a) and Model B (b) of JMJD3. The protein is represented by molecular surface and tube. **8** is depicted by sticks (pink) and balls. The atom color codes are: C (**8**), pink; C (JMJD3), grey; polar H, sky blue; N, dark blue; O, red. The dashed black lines indicate the hydrogen bonds between ligand and protein.

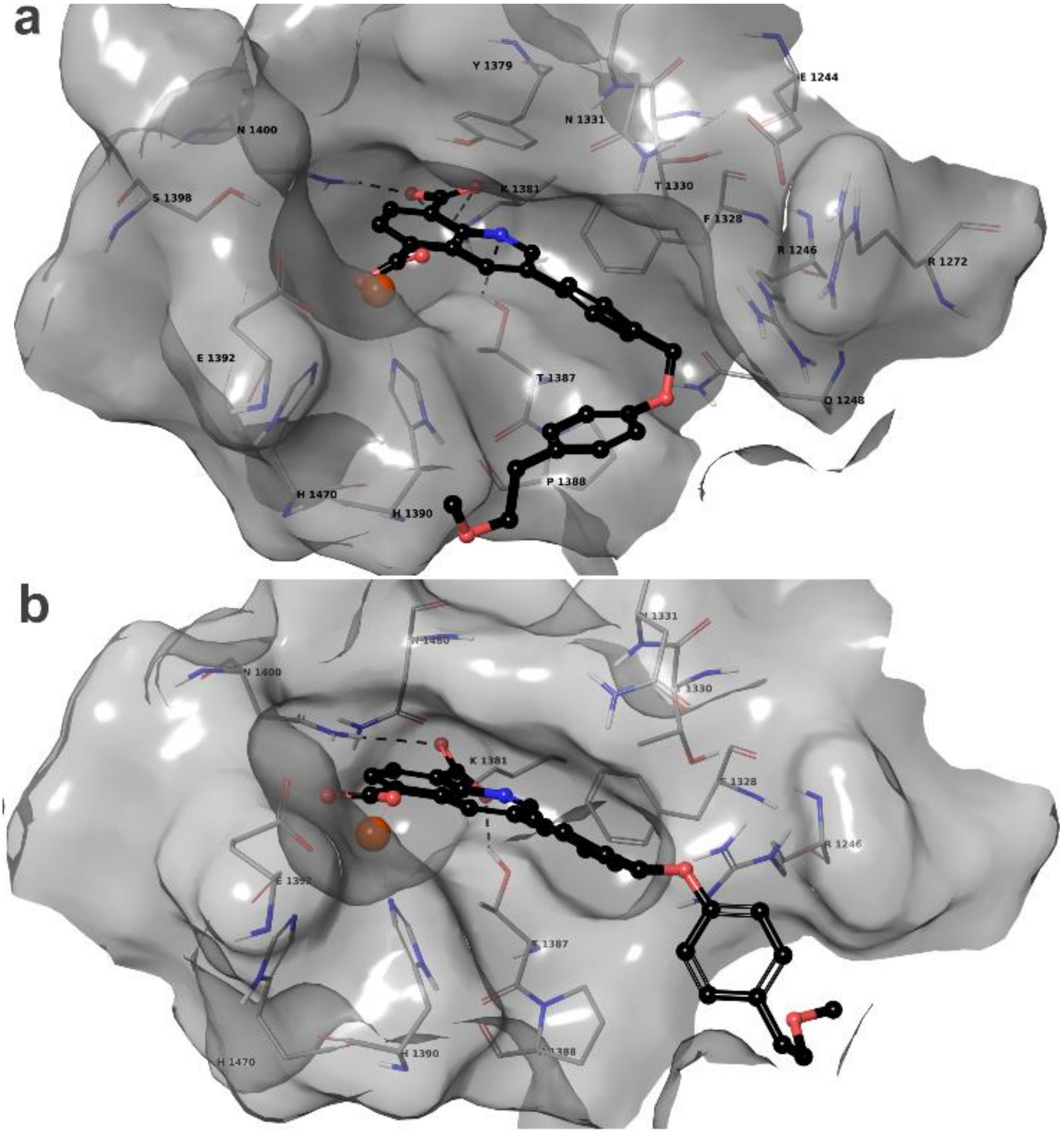

Figure S9. 3D model of the interactions between **9** and the Model A (a) and Model B (b) of JMJD3. The protein is represented by molecular surface and tube. **9** is depicted by sticks (black) and balls. The atom color codes are: C (**9**), black; C (JMJD3), grey; polar H, sky blue; N, dark blue; O, red. The dashed black lines indicate the hydrogen bonds between ligand and protein.

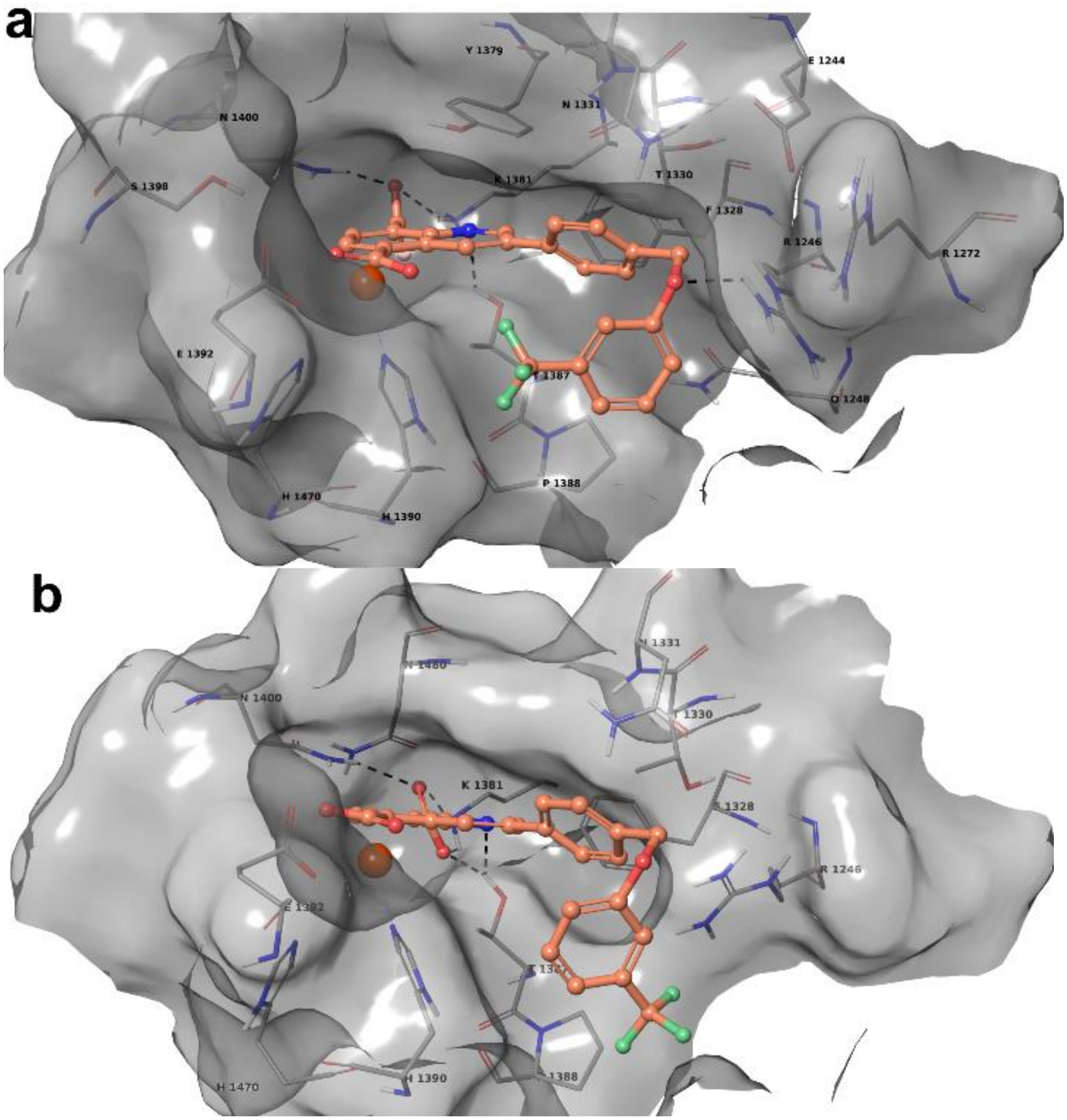

Figure S10. 3D model of the interactions between **10** and the Model A (a) and Model B (b) of JMJD3. The protein is represented by molecular surface and tube. **10** is depicted by sticks (light brown) and balls. The atom color codes are: C (**10**), light brown; C (JMJD3), grey; polar H, sky blue; N, dark blue; O, red. The dashed black lines indicate the hydrogen bonds between ligand and protein.

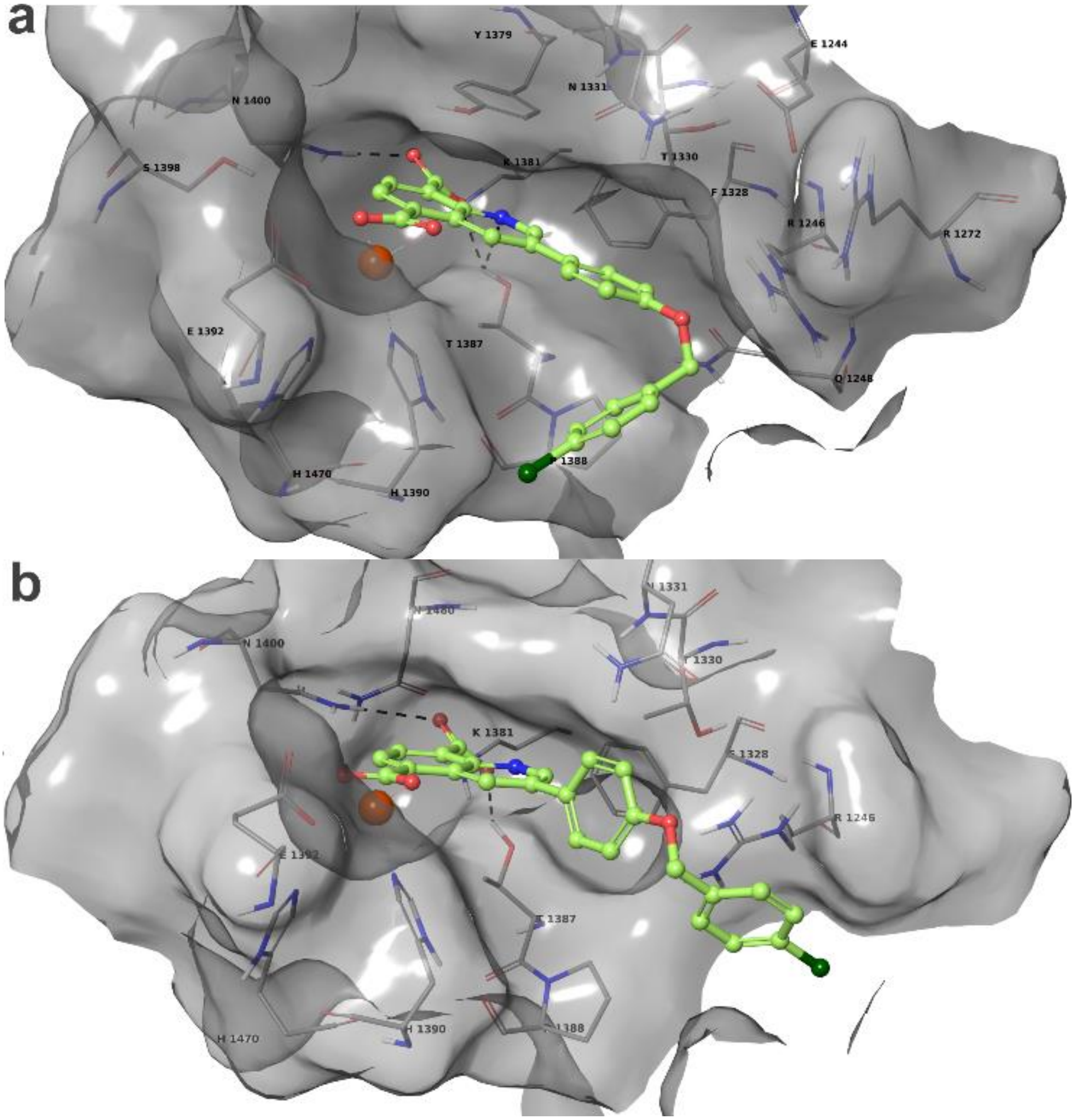

Figure S11. 3D model of the interactions between **11** and the Model A (a) and Model B (b) of JMJD3. The protein is represented by molecular surface and tube. **11** is depicted by sticks (faded yellow-green) and balls. The atom color codes are: C (**11**), faded yellow-green; C (JMJD3), grey; polar H, sky blue; N, dark blue; O, red. The dashed black lines indicate the hydrogen bonds between ligand and protein.

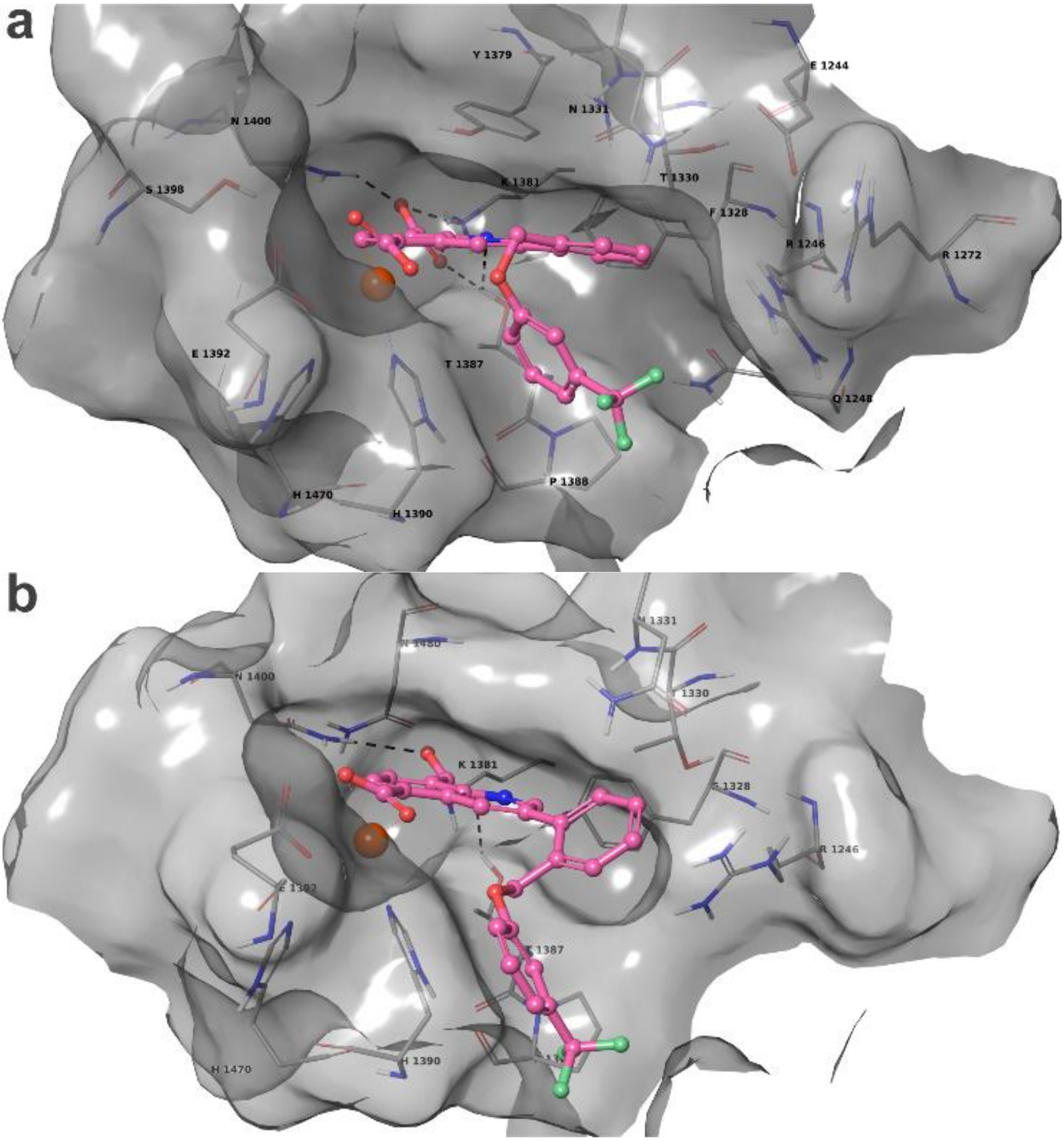

Figure S12. 3D model of the interactions between **12** and the Model A (a) and Model B (b) of JMJD3. The protein is represented by molecular surface and tube. **12** is depicted by sticks (faded salmon) and balls. The atom color codes are: C (**12**), faded salmon; C (JMJD3), grey; polar H, sky blue; N, dark blue; O, red. The dashed black lines indicate the hydrogen bonds between ligand and protein.

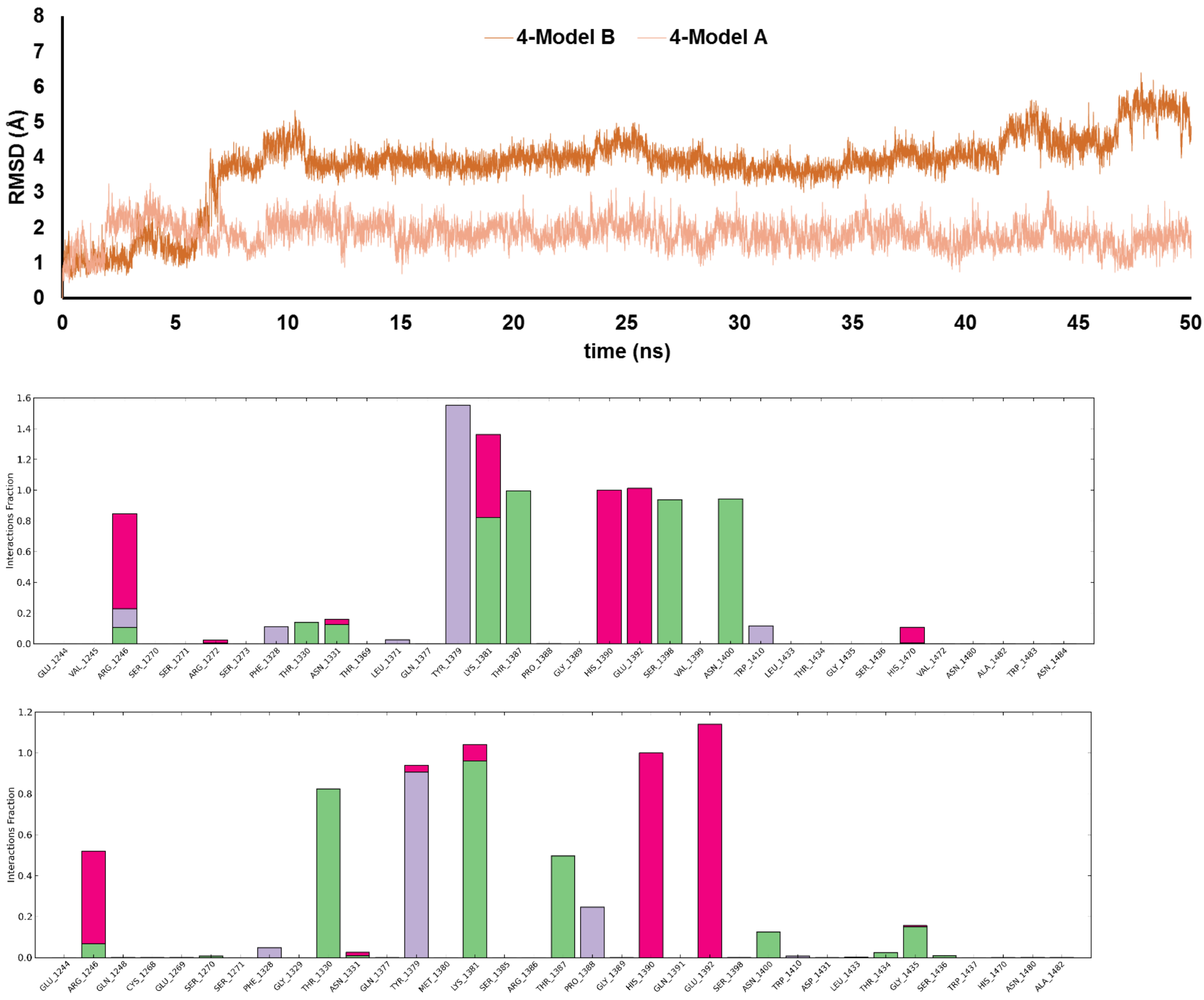

Figure S13. Heavy atom-positional RMSD (Å) of **4** bound to Model A (salmon line) and Model B (brown line) as function of simulation time (50 ns, 310 K). (b) Protein-ligand contacts histograms during the simulation of **4**-Model A and **4**-Model B.

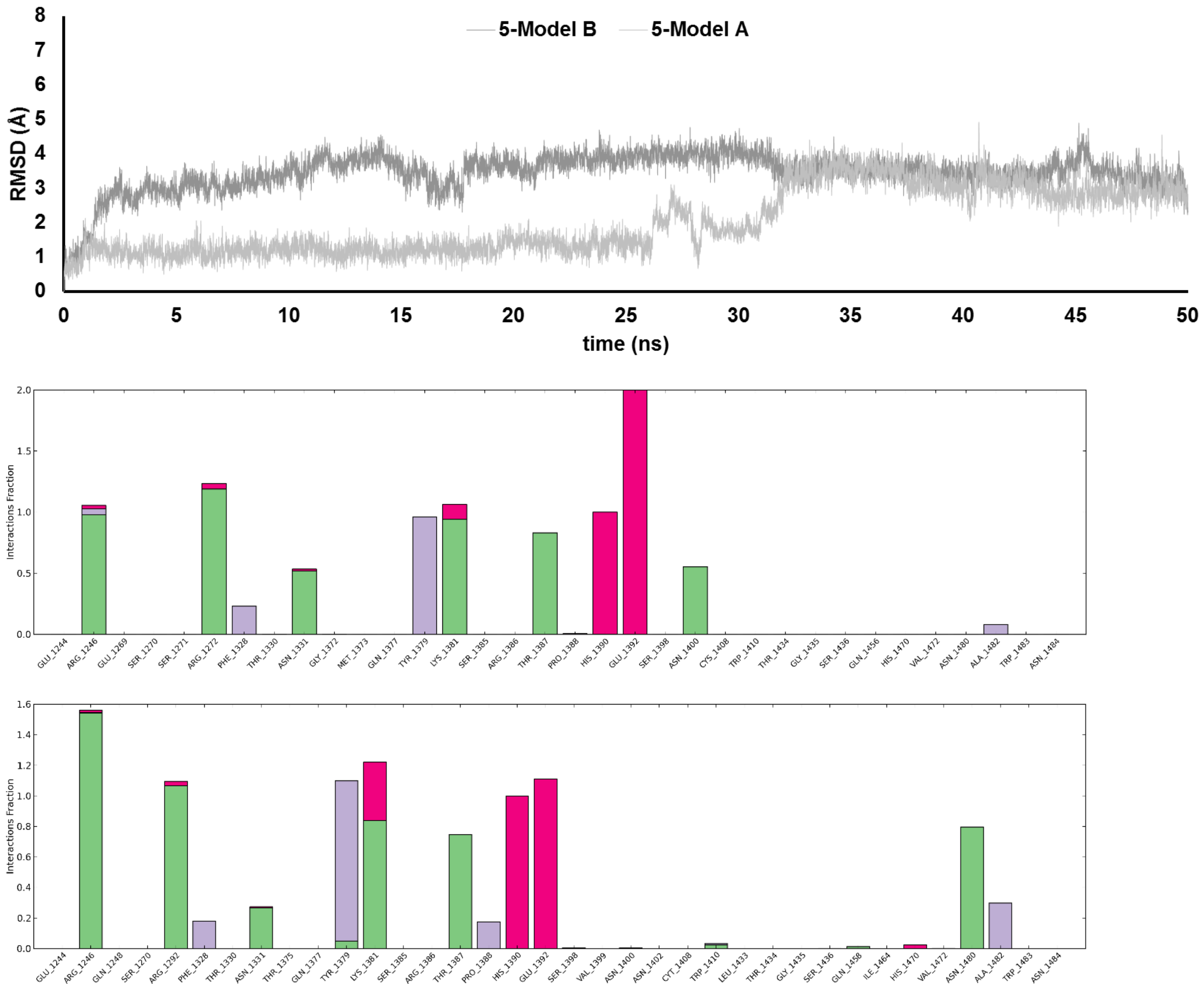

Figure S14. Heavy atom-positional RMSD (Å) of **5** bound to Model A (light grey line) and Model B (dark grey line) as function of simulation time (50 ns, 310 K). (b) Protein-ligand contacts histograms during the simulation of **5**-Model A and **5**-Model B.

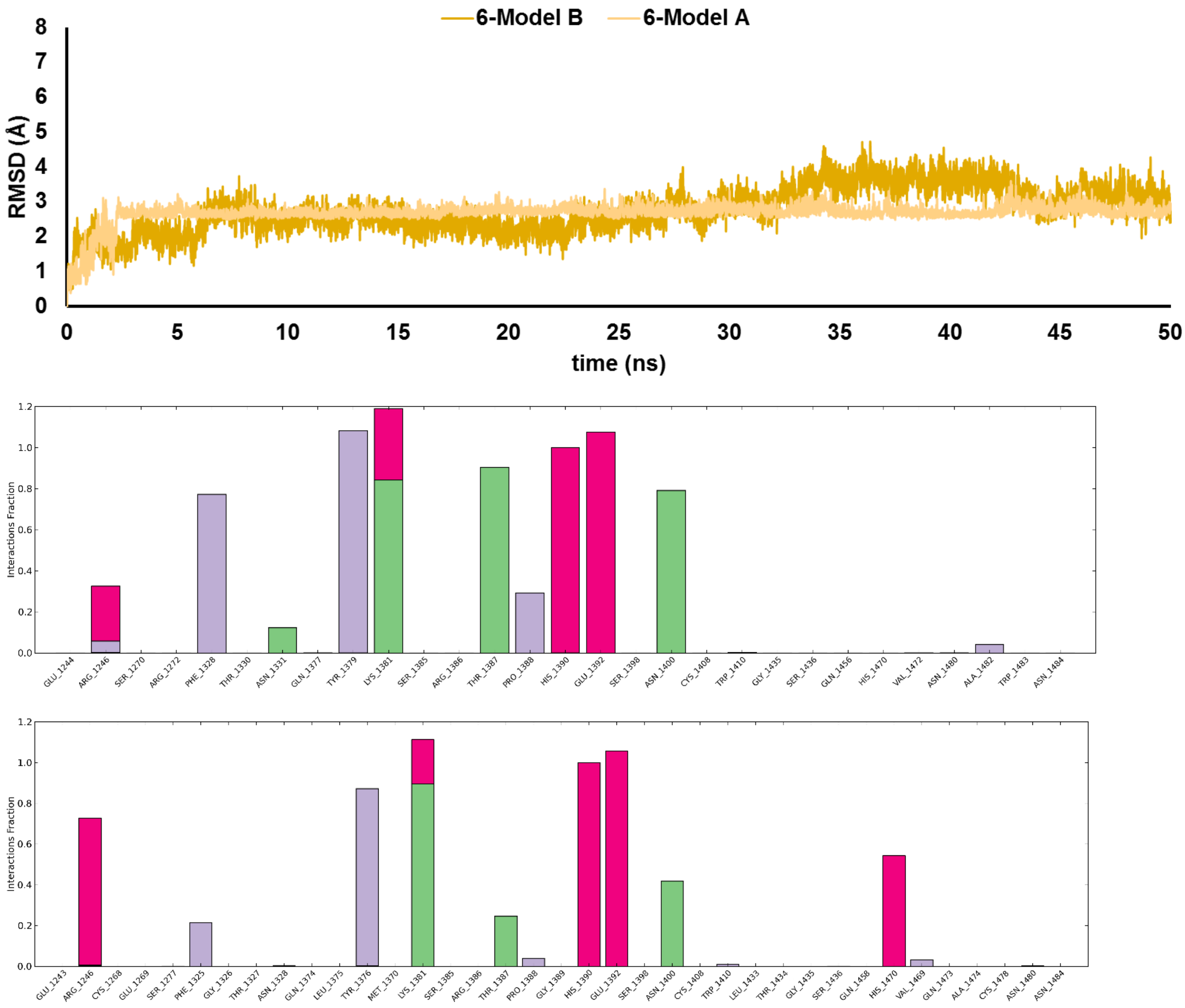

Figure S15. Heavy atom-positional RMSD (Å) of **6** bound to Model A (light yellow line) and Model B (dark yellow line) as function of simulation time (50 ns, 310 K). Protein-ligand contacts histograms during the simulation of **6**-Model A and **6**-Model B.

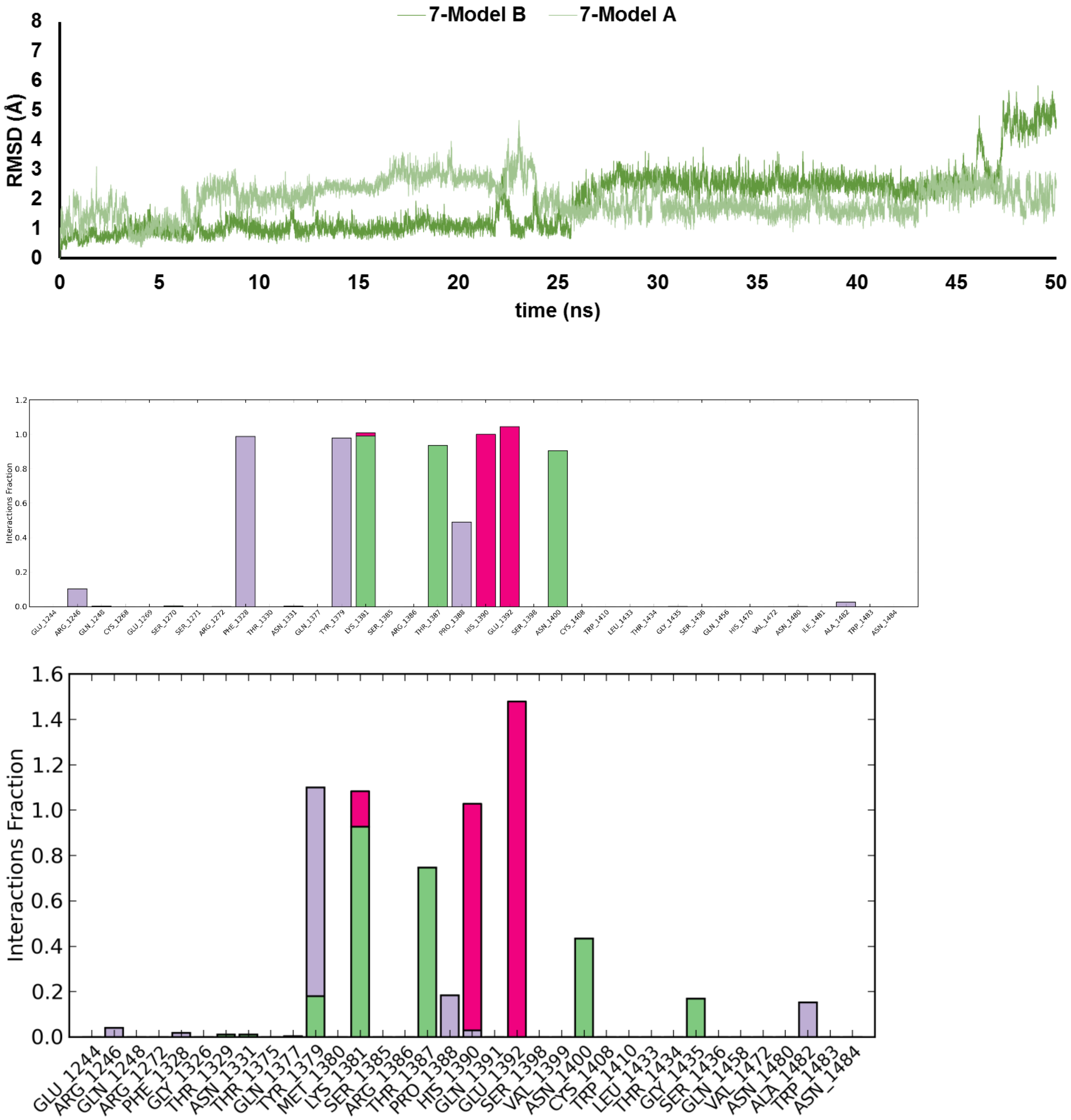

Figure S 16. Heavy atom-positional RMSD (Å) of **7** bound to Model A (light green line) and Model B (dark green line) as function of simulation time (50 ns, 310 K). Protein-ligand contacts histograms during the simulation of **7**-Model A and **7**-Model B.

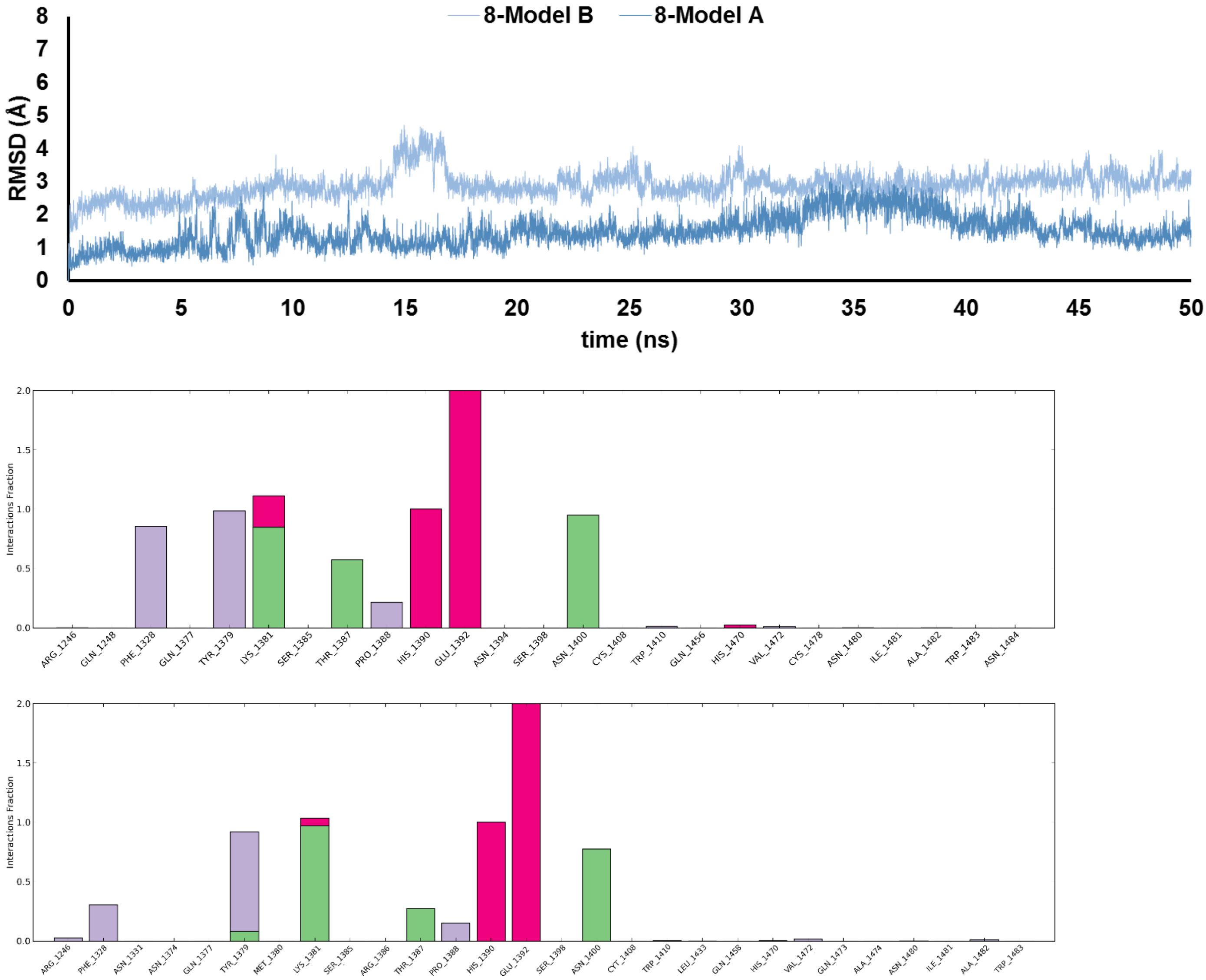

Figure S17. Heavy atom-positional RMSD (Å) of **8** bound to Model A (blue line) and Model B (cyan line) as function of simulation time (50 ns, 310 K). Protein-ligand contacts histograms during the simulation of **8**-Model A and **8**-Model B.

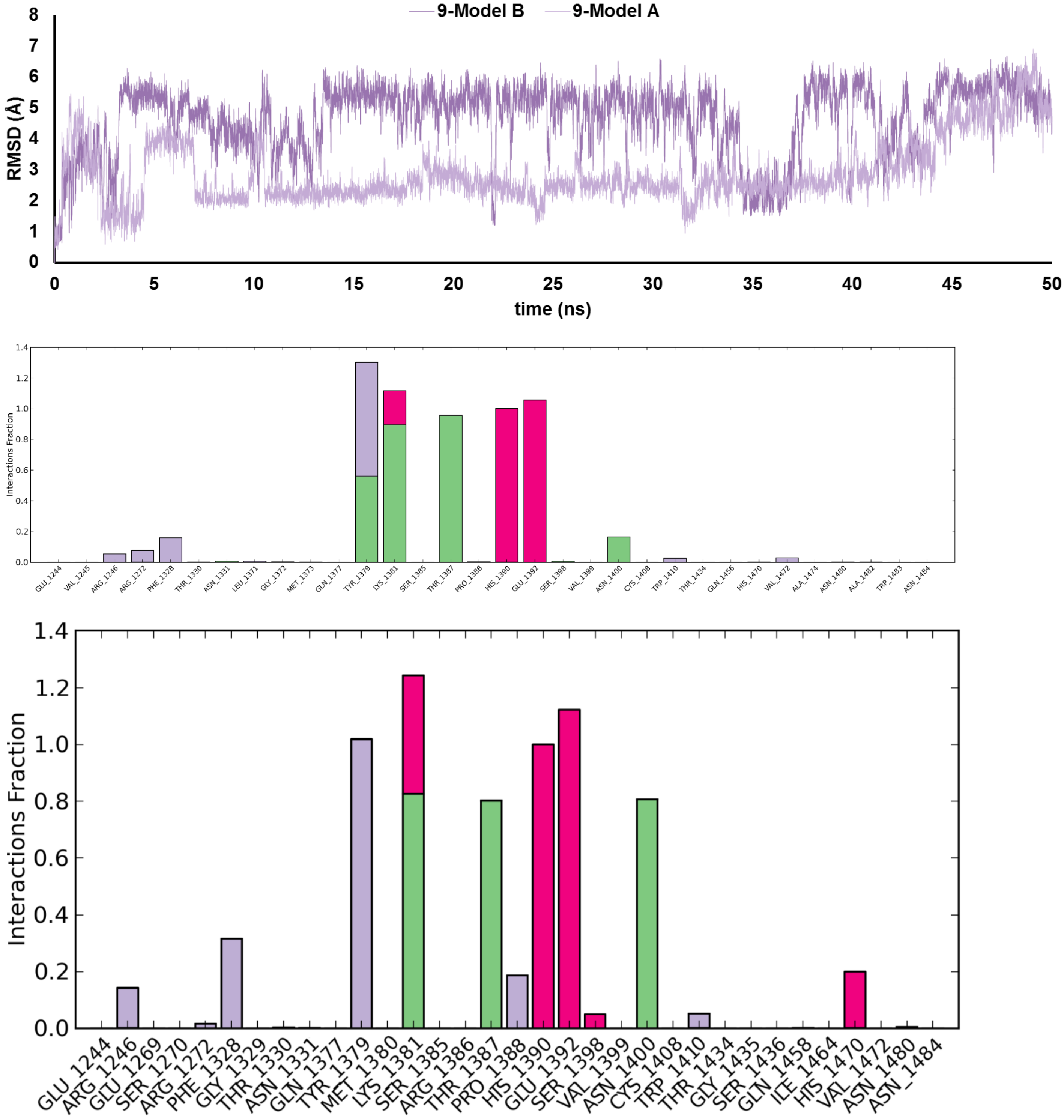

Figure S18. Heavy atom-positional RMSD (Å) of **9** bound to Model A (light violet line) and Model B (dark violet line) as function of simulation time (50 ns, 310 K). Protein-ligand contacts histograms during the simulation of **9**-Model A and **9**-Model B.

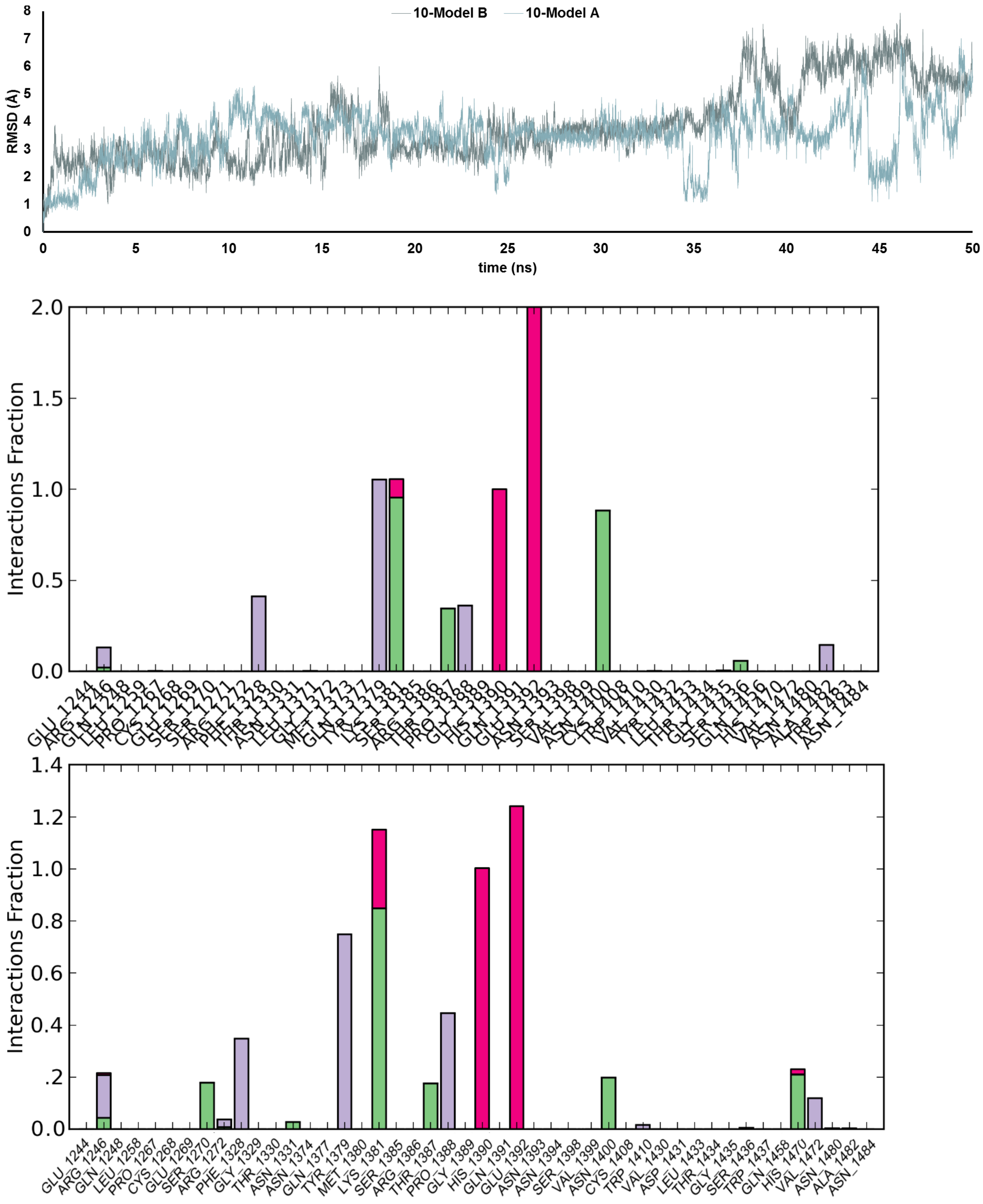

Figure S19. Heavy atom-positional RMSD (Å) of **10** bound to Model A (light blue line) and Model B (grey line) as function of simulation time (50 ns, 310 K). Protein-ligand contacts histograms during the simulation of **10**-Model A and **10**-Model B.

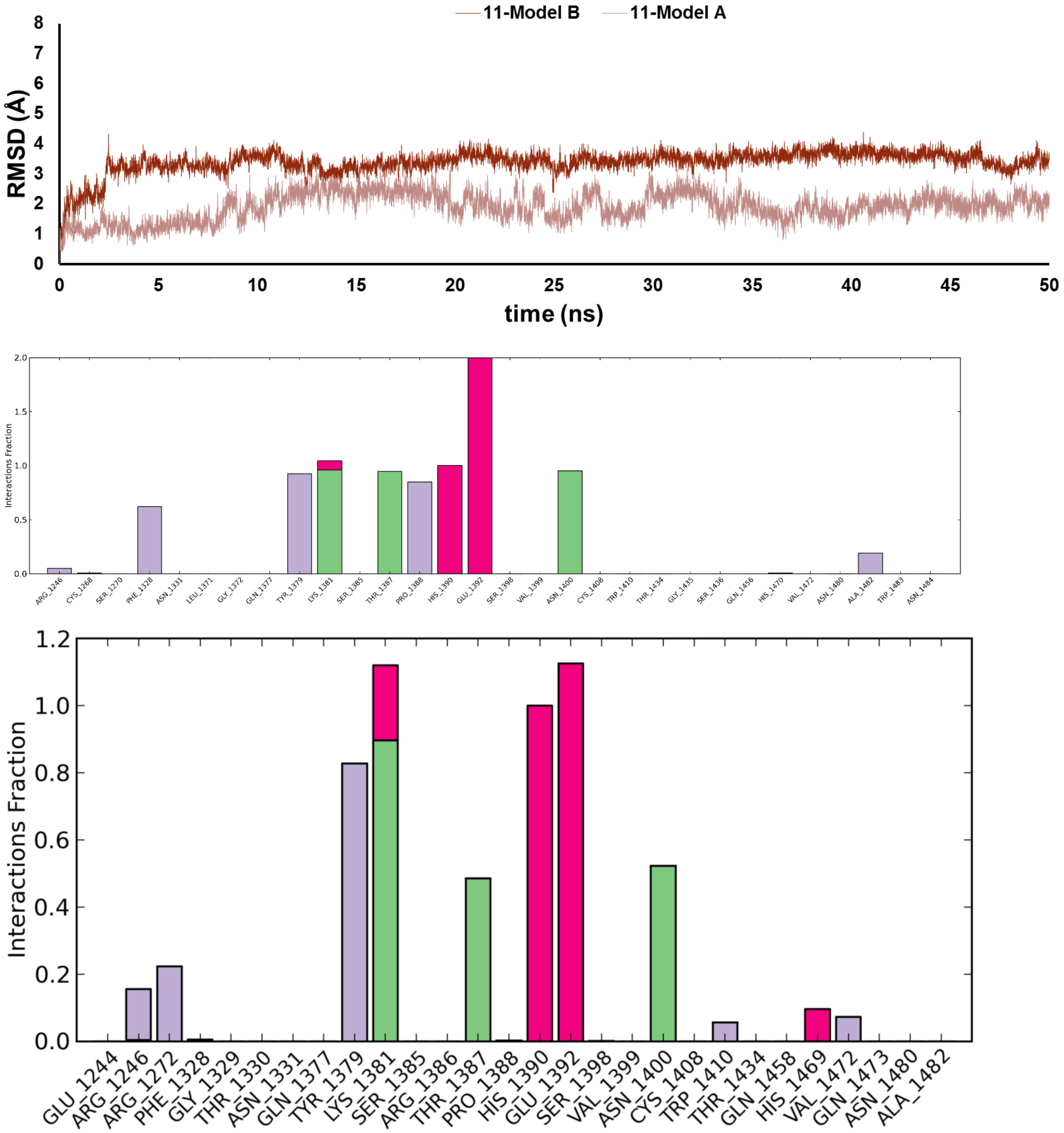

Figure S20. Heavy atom-positional RMSD (Å) of **11** bound to Model A (light brown line) and Model B (dark brown line) as function of simulation time (50 ns, 310 K). Protein-ligand contacts histograms during the simulation of **11**-Model A and **11**-Model B.

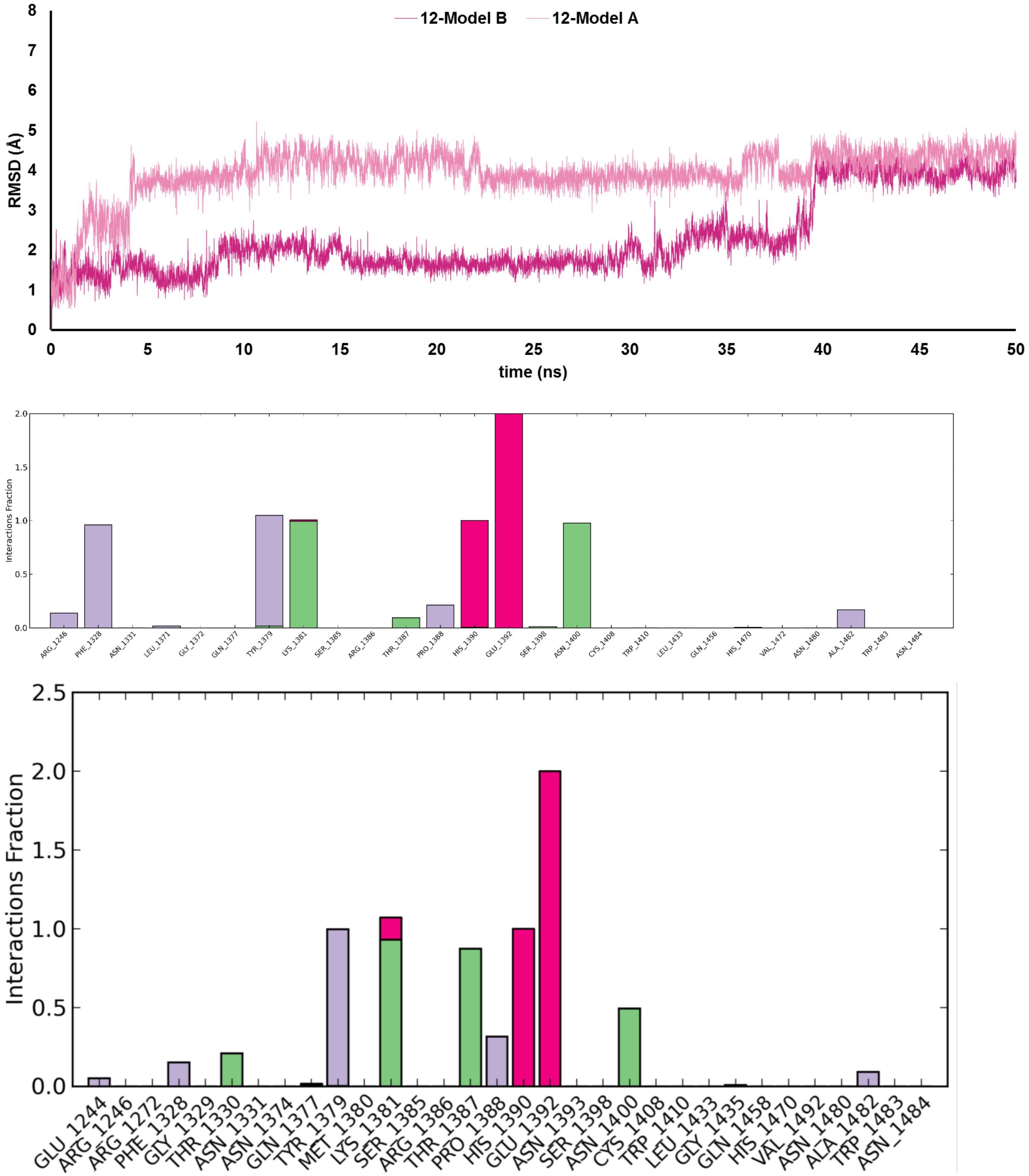

Figure S21. Heavy atom-positional RMSD (Å) of **12** bound to Model A (light pink line) and Model B (dark pink line) as function of simulation time (50 ns, 310 K). Protein-ligand contacts histograms during the simulation of **12**-Model A and **12**-Model B.

Experimental details

Quantum mechanical calculations

In order to get an accurate electrostatic contribution in molecular docking calculations, we obtained the partial charge of Fe^{2+} and of the coordinating amino acids (Table S1) by DFT theory using: M05 functional,[1] 6-31+G(d) basis set and ChelpG method[2] for population analysis (GAUSSIAN 09 Software Package).[3] The DFT derived partial charges were used in the subsequent docking calculations.

Table S1. Charges of amino acids constituting the catalytic site of JMJD3 for Model A (PDB ID 4ASK) and Model B (PDB ID 2XXZ), calculated at DFT M05 level and 6-31+G(d) basis set by using the ChelpG method for population analysis.

		JMJD3	
		Molde A	Model B
residue	PDB atom name	charge	charge
HIS1390	CB	–0.063	–0.025
	CD2	–0.043	0.363
	ND1	–0.086	–0.049
	CG	0.400	–0.040
	CE1	0.539	0.345
	NE2	–1.18	–0.990
	HD1	0.269	0.258
GLU1392	CB	–0.026	–0.216
	CG	0.174	0.449
	CD	0.777	0.436
	OE1	–0.968	–0.728
	OE2	–0.872	–0.790
HIS1470	CB	–0.075	–0.091
	CD2	0.143	0.135
	ND1	–0.591	–0.675
	CG	0.205	0.202
	CE1	0.63	0.723
	NE2	–0.629	–0.622
	HD1	0.384	0.391
	FE	1.474	1.474

Molecular modeling

Maestro version 11[4] was used to build all small molecule structures. All ligand structures (**A1-A12**, **B1-B12**, **C1-C12**, **D1-D12**, **E1-E12**, **F1-F12**, **G1-G12**, **H1-H12**, **B11'**, **3-12**, **GSK-J1**) were optimized through the MacroModel 11.5,[5] using the OPLS3 force field[6] and the Polak-Ribier conjugate gradient algorithm (PRCG, maximum derivative less than 0.001 kcal/mol). All representative conformations for each ligand were refined by using the Polak-Ribier conjugate gradient algorithm (maximum derivative less than 0.001 kcal/mol). To mimic the presence of H_2O, the GB/SA (generalized Born/surface area)[7] solvent treatment was used, in the geometry optimization and in the conformational search calculations. All

ligands were processed by LigPrep,[8] generating all possible stereoisomers, tautomers, and protonation states at a pH of 7.0 ± 1.0.
As protein models, we used two crystal structures of JMJD3 (PDB IDs: 4ASK and 2XXZ).[9] The proteins were processed by means of Protein Preparation Wizard[10,11] from Schrödinger suite: all hydrogens were added; bond order were assigned; checking of alternate position of residues and of missing side chains and loops. The Co^{2+} and Ni^{2+} ions, respectively from 4ASK and 2XXZ X-ray structures, were replace by Fe^{2+}. The charges of amino acid side chains were assigned taking into account their pK_a at pH 7.0. The network of hydrogen bonds was refined through the optimize option of Protein Preparation Wizard. All water molecules were eliminated. Docking calculations were performed by means of the software AutoDock 4.2.[12,1314] The protein models were processed by AutoDockTools1.5.7rc1:[15] all non-polar hydrogens were merged and Gasteiger charges were added. For Fe^{2+} and of the coordinating amino acids, we used the calculated DFT partial charges. For Model A, a grid box size of 54 x 46 x 54 was applied, with a grid center having the x, y, and z coordinates: 45.578, 44.880, 7.00. For Model B, the grid box was sized as 48 x 52 x 54, and centred at the following coordinates: 44.299, 44.964, 8.492. The grid points for both boxes were spaced by 0.375 Å. In order to validate our docking methodology, we docked the co-crystallized GSK-J1 with JMJD3, obtaining an RMSD 0.81 Å for the docked pose respect to the crystallized (PDB ID: 4ASK, Figure S22) conformation. Calculations consisting of 20 runs were performed, by applying the Lamarckian genetic algorithm. An initial population of 150 randomly placed individuals was used. The maximum number of energy evaluations and of generations was set up at 1750000 and 2700, respectively. A mutation rate of 0.02 and a crossover rate of 0.8 were used, and the local search frequency was set up at 0.26. Results differing by less than 2.0 Å in positional root-mean-square deviation (rmsd) were clustered together and ranked by free energy of binding.
All bonds were treated as active torsional bonds except the double and ester bonds. Docking results were analysed with Auto-DockTools 1.5.7rc1 and figures obtained by using Maestro 11.
For AutoLigand[16,17] analysis, the above reported grid boxes were used but the grid points were spaced of 1.0 Å. For the ligand atom types, the C, HD and OA maps were calculated. Affinity envelopes were obtained ranging the number of point from 10 to 500.

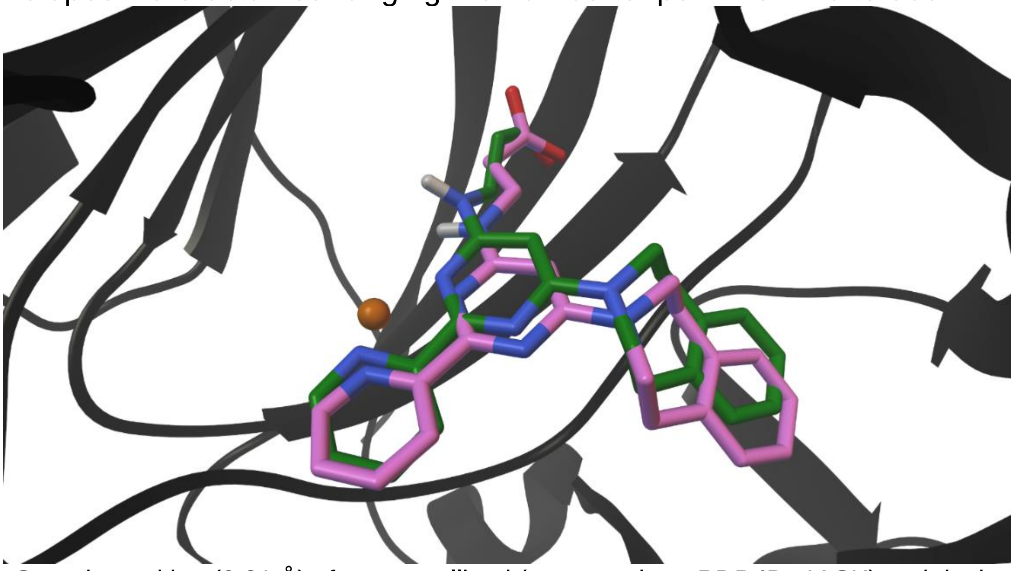

Figure S22. Superimposition (0.81 Å) of co-crystallized (green carbon, PDB ID: 4ASK) and docked pose (pink carbon) structures of GSK-J1. The protein is depicted by black ribbons and the Fe^{2+} is represented in orange cpk. The ligand is represented by tube (colored: C, as for the tube; polar H, white; N, dark-blue; O, red).

Molecular dynamics simulations

The docked poses of **3-12** bound to Models A and B were used as input structures for molecular dynamics simulations. The starting complexes were firstly processed with Protein Preparation Wizard and then were prepared by System Builder[18] in Desmond.[19,20] A cubic box with a 10 Å buffer distance, resulting in a system with approximately 43317 atoms for Model A and 50345 for Model B. The TIP3P[21] water model for solvation and OPLS-2005 force field were used,[22] and Na^+ ions were added for electroneutrality. The so built systems were minimized by LBFGS method, with a maximum number of 2000 iterations and a convergence threshold of 1.0 kcal/mol/Å. Then, the minimized systems were underwent the following relaxation protocol: 1) 1 ns of NVT simulation at 10 K, with small time steps and solute non-hydrogen atoms restrained; 2) 120 ps of NVT simulation using a Berendsen thermostat at 10 K, with fast temperature relaxation constant, a velocity resampling every 1 ps and non-hydrogen solute atoms restrained; 3) 120 ps of NPT simulation using a Berendsen thermostat and a Berendsen barostat at 10 K and a pressure of 1 atm (fast temperature relaxation constant, a slow pressure relaxation constant, velocity resampling every 1 ps, non-hydrogen solute atoms restrained); 4) 120 ps of simulate in the NPT ensemble using a Berendsen thermostat and a Berendsen barostat at 300 K and 1 atm, with a fast temperature relaxation constant, a slow pressure relaxation constant, velocity resampling every 1 ps and non-hydrogen solute atoms restrained; 5) 240 ps of NPT simulation using a Berendsen thermostat and a Berendsen barostat at 300 K and 1 atm, with a fast temperature relaxation constant and a normal pressure relaxation constant. MD simulations of 50 ns at 310 K, using a recording interval of 1.2 ps and an ensemble class NPT (1.01 bar). A 2.0 fs integration time step was used. Each step of equilibration protocol was checked by Simulation Quality Analysis tool of Desmond, monitoring the total energy, potential energy, temperature, pressure and volume. The exa-coordination of Fe^{2+} was also checked.

Chemistry

(a) General information:

All commercially available starting materials were purchased from Sigma-Aldrich and were used without any purification. All solvents used for the synthesis were of HPLC grade; they were purchased from Sigma-Aldrich and Carlo Erba Reagenti. ^1H NMR spectra were recorded on 300, 400, 500 and 600MHz Bruker Advance instrument. All compounds were dissolved in 0.5 mL of the following solvents: chloroform-d (Sigma-Aldrich, 99.8 Atom % D); methanol-d_4 (Sigma-Aldrich, 99.8 Atom % D); dimethylsulfoxide-d_6 (Sigma-Aldrich, 99.96 Atom % D). Coupling constants (*J*) are reported in Hertz, and chemical shifts are expressed in parts per million (ppm) on the delta (δ) scale relative to $CHCl_3$ (7.26 ppm for ^1H and 77.2 ppm for ^{13}C) or CH_3OH (3.31 ppm for ^1H and 49.15 ppm for ^{13}C) or DMSO (2.50 ppm for ^1H

and 39.51 ppm for ^{13}C) as internal reference. Multiplicities are reported as follows: s, singlet; d, doublet; t, triplet; m, multiplet; dd, doublet of doublets. ^{13}C NMR spectra were obtained at 100 MHz and referenced to the internal solvent signals. Electrospray mass spectrometry (ESIMS) was performed on a LCQ DECA TermoQuest (San Josè, California, USA) mass spectrometer. High Resolution Mass Spectrometry spectra were recorded on an Agilent 6545 Accurate-Mass Q-TOF LC/MS system. Reactions were monitored on silica gel 60 F254 plates (Merck) and the spots were visualized under UV light. Analytical and semi-preparative reversed-phase HPLC was performed on Agilent Technologies 1200 Series high performance liquid chromatography using a Synergi Fusion C18reversed-phase column (250 x 4.60mm, 4μ, 80 Å, flow rate = 1 mL/min; 250 x 10.00mm, 10μ, 80 Å, flow rate = 4 mL/min respectively, Phenomenex®). The binary solvent system (A/B) was as follows: 0.1% TFA in water (A) and 0.1% TFA in CH_3CN (B); gradient condition: from 5% B to 100 % B in 50 min. The absorbance was detected at 280 nm. The purity of all tested compounds (>96%) was determined by HPLC analysis.

b) Preparation of 3-Bromo-5,8-dicarboxy-quinoline (2)

2,2,3-Tribromopropanal (1)

Br_2 (10.3 mL, 31.9 g, 0.2 mol) was added dropwise over 2 h to a solution of acrolein (13.3 mL, 11.2 g, 0.2 mol) in DCE (100 mL) at r.t. During the addition, the color changed from colorless to orange and the reaction temperature was kept in the range 20–40 °C. Following addition, the mixture was stirred for 2 h at r.t., then another equivalent of Br_2 (10.3 mL) was added dropwise over 2 h. The red– brown mixture was stirred for 16 h at r.t. and evaporated under reduced pressure to deliver 2,2,3-tribromopropanal (**1**) (49.4 g, 0.17 mol, 84%) as a slightly yellowish liquid.

^{1}H NMR (400 MHz, CDCl3): δ = 4.23 (s, 2 H), 9.16 (s, 1 H).

3-Bromo-5,8-dicarboxy-quinoline (2)

2,2,3-Tribromopropanal (**1**) (29.4 g, 0.1 mol) was added slowly to a suspension of 2-aminoterephtalic acid (18.1 g, 0.1 mol) in glacial AcOH (200 mL). The mixture was heated to 110 °C for 1 h, cooled to r.t., diluted with AcOEt (200 mL) and filtered. The remaining solid was purified by chromatography on silica gel, using CH_2Cl_2 - MeOH 80/20 as eluent to afford 5,8-dicarboxy-quinoline (**2**) (22.7 g, 0.077 mol, 77%) as orange crystals.

^{1}H NMR (400MHz, CD_3OD) 9.81 (d, J = 2.4 Hz, 1H); 9.09 (d, J = 2.3 Hz, 1H); 8.66 (d, J = 7.6 Hz, 1H); 8.47 (d, J = 7.7 Hz, 1H).

c) Typical Procedure for the Cross-Coupling Reactions in Scheme 1.

The boronic acid (1.2 equiv), the aryl halide (1.0 equiv), and $Pd(PPh_3)_4$ as catalyst (ca. 5 mol %) were sequentially added to degassed 1,4-dioxane (10 mL) and the mixture was

stirred at 20 °C for 30 min. Degassed aqueous K_2CO_3 solution (1 M, 2.0 equiv) was added and the reaction mixture was heated under nitrogen at 85 °C for 18 h. Solvent was removed in vacuo, then ethyl acetate was added and the organic layer was washed with brine, separated, and dried over $MgSO_4$. The solvent was removed under reduced pressure, and the residue was purified by semi-preparative reversed-phase. The final products were obtained with high purity (>98%) detected by HPLC analysis and were fully characterized by ESI-MS, and NMR spectra.

d) Characterization data for all compounds

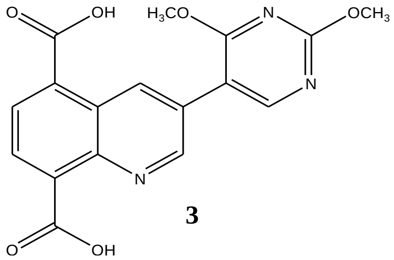

3

3-(2,4-Dimethoxy-pyrimidin-5-yl)-quinoline-5,8-dicarboxylic acid (3)

Compound **3** was obtained as a red solid following the general procedure using 2,4-dimethoxy-5-pyrimidinylboronic acid (67% yield). RP-HPLC t_R = 20.4 min.

^{1}H NMR (DMSO, 600 MHz): 9.63 (d, *J* = 1.7 Hz, 1H); 9.37 (d, *J* = 1.7 Hz, 1H); 8.70 (s, 1H); 8.54 (d, *J* = 7.6 Hz, 1H); 8.43 (d, *J* = 7.6 Hz, 1H); 4.03 (s, 3H); 4.01 (s, 3H).

^{13}C NMR (DMSO, 125 MHz):168.3, 167.3, 166.6, 165.4, 159.2, 150.3, 143.7, 135.8, 132.4, 131.1, 129.7, 128.6, 126.4, 112.1, 55.4, 54.9.

HRMS, calcd for $C_{17}H_{13}N_3O_6$ 355.08; found m/z = 356.0977 $[M+ H]^+$.

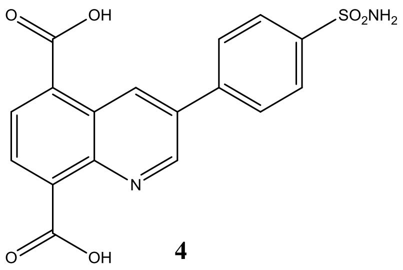

4

3-(4-Sulfamoyl-phenyl)-quinoline-5,8-dicarboxylic acid (4)

Compound **4** was obtained as a brown solid following the general procedure using (4-aminosulfonylphenyl)boronic acid (72% yield). RP-HPLC t_R = 22.0 min.

^1H NMR ($CDCl_3/CD_3OD$ 1:1, 300 MHz)

9.93 (d, *J* = 2.5 Hz, 1H); 9.33 (d, *J* = 2.3 Hz, 1H); 8.76 (d, *J* = 7.6 Hz, 1H); 8.53 (d, *J* = 7.7 Hz, 1H); 8.09 (d, *J* = 8.6 Hz, 2H); 7.95 (d, *J* = 8.6 Hz, 2H).

HRMS, calcd for $C_{17}H_{12}N_2O_6S$ 372.04; found m/z = 373.0624 $[M + H]^+$.

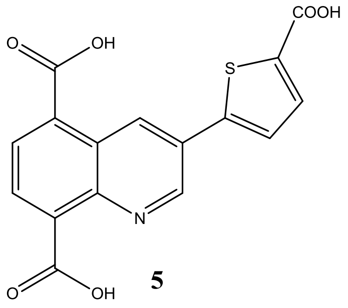

5

3-(5-Carboxy-thiophen-2-yl)-quinoline-5,8-dicarboxylic acid (5)

Compound **5** was obtained as a white solid following the general procedure using 5-(dihydroxyboryl)-2-thiophenecarboxylic acid (53% yield). RP-HPLC t_R = 21.5 min.

^1H NMR (DMSO, 500 MHz)

9.94 (d, *J* = 2.4 Hz, 1H); 9.48 (d, *J* = 2.4 Hz, 1H); 8.73 (d, *J* = 7.9 Hz, 1H); 8.55 (d, *J* = 7.6 Hz, 1H); 7.85 (d, *J* = 3.7 Hz, 1H); 7.80 (d, *J* = 4.0 Hz, 1H).

ESMS, calcd for $C_{16}H_9NO_6S$ 343.02; found m/z 344.2 = $[M + H]^+$.

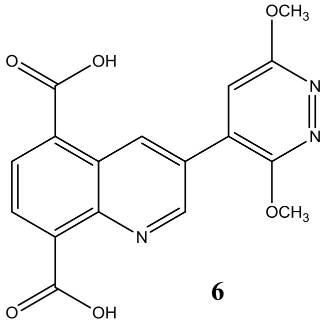

6

3-(3,6-Dimethoxy-pyridazin-4-yl)-quinoline-5,8-dicarboxylic acid (6)

Compound **6** was obtained as a white solid following the general procedure using 3,6-dimethoxylpyridazine-4-boronic acid (65.0% yield). RP-HPLC t_R = 22.1 min.

^1H NMR (DMSO, 300 MHz)

9.71 (d, *J* = 2.1 Hz, 1H); 9.36 (d, *J* = 2.2 Hz, 1H); 8.51 (d, *J* = 7.6 Hz, 1H); 8.40 (d, *J* = 7.6 Hz, 1H); 7.58 (s, 1H); 4.02 (s, 3H); 4.00 (s, 3H).

ESMS, calcd for $C_{17}H_{13}N_3O_6$355.08; found m/z = 356.3 $[M+ H]^+$.

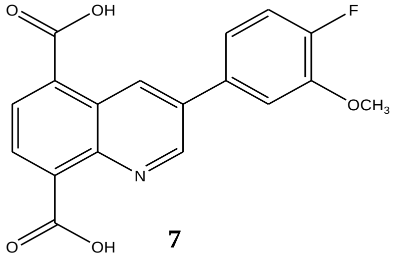

7

3-(4-Fluoro-3-methoxy-phenyl)-quinoline-5,8-dicarboxylic acid (7)

Compound **7** was obtained as a white solid following the general procedure using (4-fluoro-3-methoxyphenyl)boronic acid (68% yield). RP-HPLC t_R = 22.8 min.

^{1}H NMR (DMSO, 300 MHz)

9.65 (d, *J* = 2.3 Hz, 1H); 9.54 (d, *J* = 2.3 Hz, 1H); 8.54 (d, *J* = 7.7 Hz, 1H); 8.43 (d, *J* = 7.6 Hz, 1H); 7.70 (d, *J* = 7.4 Hz, 1H); 7.54 – 7.30 (m, 2H); 3.98 (s, 3H).

ESMS, calcd for $C_{18}H_{12}FNO_5$ 341.07; found m/z 342.11 = $[M+ H]^+$.

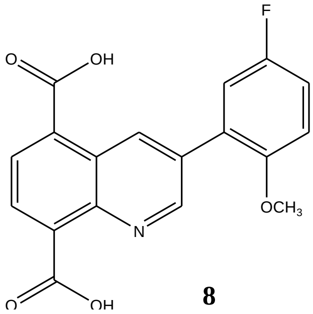

8

3-(5-Fluoro-2-methoxy-phenyl)-quinoline-5,8-dicarboxylic acid (8)

Compound **8** was obtained as a colorless oil following the general procedure using 5-fluoro-2-methoxyphenylboronic acid (70% yield). RP-HPLC t_R = 22.8 min.

^{1}H NMR (CDCl3/CD_3OD 1:1, 300 MHz)

9.42 (d, *J* = 2.2 Hz, 1H); 9.16 (d, *J* = 2.2 Hz, 1H); 8.66 (d, *J* = 7.6 Hz, 1H); 8.08 (d, *J* = 7.5 Hz, 1H); 7.27 (dd, *J* = 8.8, 3.0 Hz, 1H), 7.12 (td, *J* = 8.4, 3.1 Hz, 1H); 7.05 (dd, *J* = 9.1, 4.4 Hz, 1H); 3.85 (s, 3H).

ESMS, calcd for $C_{18}H_{12}FNO_5$ 341.07; found m/z 342.1 = $[M+ H]^+$.

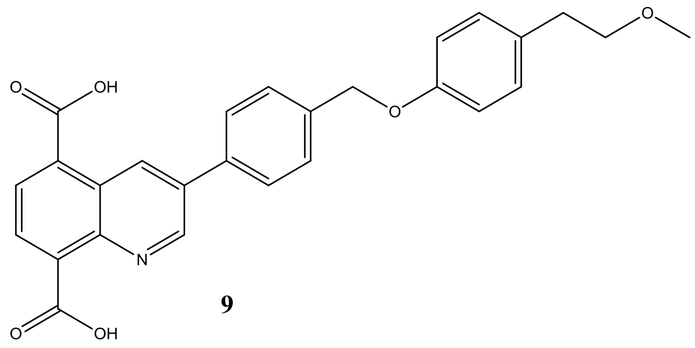

9

3-{4-[4-(2-Methoxy-ethyl)-phenoxymethyl]-phenyl}-quinoline-5,8-dicarboxylic acid (9)

Compound **9** was obtained as an oil following the general procedure using 4-[(4-(2-methoxyethyl)phenoxy)methyl]phenylboronic acid (75%yield). RP-HPLC t_R = 32.7 min.

^{1}H NMR (DMSO, 300 MHz)

9.68 (d, *J* = 2.3 Hz, 1H); 9.51 (d, *J* = 2.3 Hz, 1H); 8.52 (d, *J* = 7.7 Hz, 1H); 8.42 (d, *J* = 8.1 Hz, 1H); 7.90 (d, *J* = 8.3 Hz, 2H); 7.66 (d, *J* = 7.8 Hz, 2H); 7.15 (d, *J* = 8.2 Hz, 2H); 6.95 (d, *J* = 8.5 Hz, 2H); 5.18 (s, 2H); 3.47 (t, *J* = 6.7 Hz, 3H); 2.73 (t, *J* = 6.8 Hz, 3H).

ESMS, calcd for C27H23NO6 457.15; found m/z 458.30 = $[M+ H]^+$.

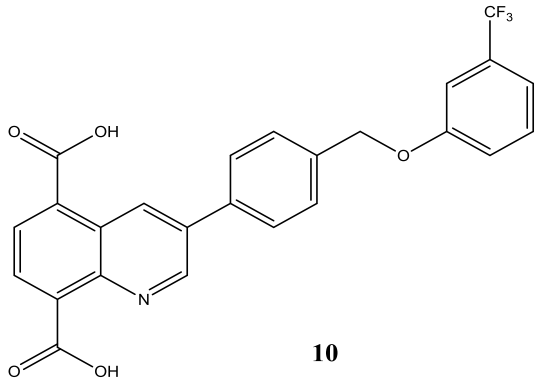

10

3-[4-(3-Trifluoromethyl-phenoxymethyl)-phenyl]-quinoline-5,8-dicarboxylic acid (10)

Compound **10** was obtained as an oil following the general procedure using 4-(3-(trifluoromethyl)phenoxymethyl)phenylboronic acid (70% yield). RP-HPLC t_R = 35.5 min.

^{1}H NMR (DMSO, 300 MHz)

9.69 (d, *J* = 2.2 Hz, 1H); 9.52 (d, *J* = 2.3 Hz, 1H); 8.53 (d, *J* = 7.6 Hz, 1H): 7.94 (d, *J* = 8.1 Hz, 2H); 7.71 (d, *J* = 8.0 Hz, 2H); 7.56 (t, *J* = 8.1 Hz, 1H); 7.42 – 7.24 (m, 4H); 5.31 (s, 2H).

ESMS, calcd for $C_{25}H_{16}F_3NO_5$ 467.10; found m/z 468.24 = $[M+H]^+$.

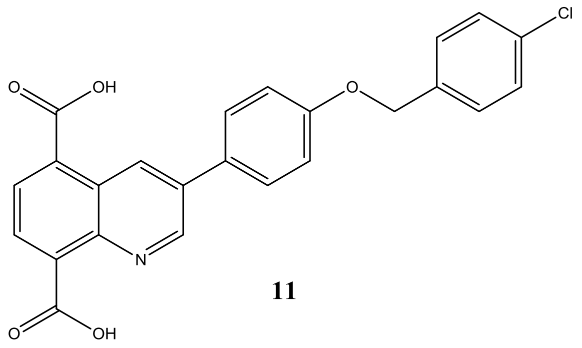

11

3-[4-(4-Chloro-benzyloxy)-phenyl]-quinoline-5,8-dicarboxylic acid (11)

Compound **11** was obtained as an oil following the general procedure using 4-(4-chlorobenzyloxy)phenylboronic acid (75% yield). RP-HPLC t_R = 35.8 min.

^{1}H NMR (DMSO, 300 MHz)

9.66 (1H, d, *J*: 2.4); 9.51 (1H, d, *J*: 2.7); 8.53 (1H, d, *J*: 7.6); 8.42 (1H, d, *J*: 7.7); 7.65 – 7.30 (m, 7H); 7.17 (1H, dd, *J*: 8.1, 2.4); 5.24 (s, 2H).

ESMS, calcd for $C_{24}H_{16}ClNO_5$ 433.07; found m/z 434.34 = $[M+H]^+$.

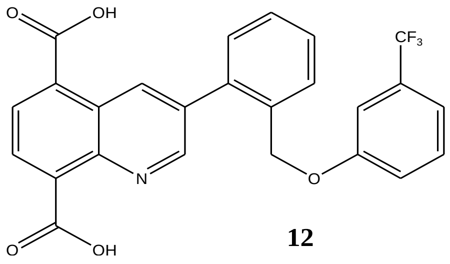

12

3-[2-(3-Trifluoromethyl-phenoxymethyl)-phenyl]-quinoline-5,8-dicarboxylic acid (12)

Compound **12** was obtained as an oil following the general procedure using 2-[(3-(trifluoromethyl)phenoxy)methyl]phenylboronic acid (73% yield). RP-HPLC t_R = 35.0 min.

^{1}H NMR (DMSO, 300 MHz)

9.47 (s, 1H); 9.23 (s, 1H); 8.53 (d, *J* = 7.7 Hz, 1H); 8.40 (d, *J* = 7.6 Hz, 1H); 7.88 – 7.69 (m, 1H); 7.57 (m, 4H); 7.40 (t, *J* = 8.0 Hz, 1H); 7.16 (m, 2H); 7.05 (s, 1H); 5.16 (s, 2H).

ESMS, calcd for $C_{25}H_{16}F_3NO_5$ 467.10; found m/z 468.2 = $[M+H]^+$.

Alphascreen
Materials
2,4-pyridine dicarboxylic acid (2,4-PC) is purchased from Acros Organics (New Jersey, Catalog number 101860010).
GSK J1 is purchased from Sigma Aldrich (Italy, Catalog number SML0709).

JIB-04 is purchased from Sigma-Aldrich (St. Louis, MO, Catalog number SML0808).

AlphaLISA anti-mIgG acceptor beads from PerkinElmer (Santa Clara, CA, Catalog number AL105C).

AlphaLISA anti-rIgG acceptor beads from PerkinElmer (Santa Clara, CA, Catalog number AL104C).

AlphaScreen Streptavidin-conjaugated donor beads from PerkinElmer (Santa Clara, CA, Catalog number 6760002).

Primary antibody 2 from BPS (Catalog number 52140B1).

Primary antibody 5 from BPS (Catalog number 52140E).

Primary antibody 6 from BPS (Catalog number 52140F).

Primary antibody 16-2 from BPS (Catalog number 52140P2).

Primary antibody 17-3 from BPS(Catalog number 52140Q3).

Biotinylated histone H3 peptide substrate (JMJD2's) (BPS).

Biotinylated histone H3 peptide substrate (Jarid's) (BPS).

Biotinylated histone H3 peptide substrate (UTX and JMJD3) (BPS and Anaspec).

Biotinylated histone H3 peptide substrate (FBXL11) (BPS).

Biotinylated histone H3 peptide substrate (JMJD1A) (BPS).

JMJD2C Assay Buffer from BPS.

JMJD2D Assay Buffer from BPS.

Jarid1A Assay Buffer from BPS.

Jarid1B Assay Buffer from BPS.

JMJD1A Assay Buffer from BPS.

JMJD3 Assay Buffer from BPS.

FBXL11 Assay Buffer from BPS.

UTX Assay Buffer from BPS.

4x Detection buffer from BPS (Catalog number 52301).

Assay conditions

All of the enzymatic reactions were conducted at room temperature for 60 minutes in a 10 µl mixture containing assay buffer, histone H3 peptide substrate, demethylase enzyme, and the test compound. These 10 µl reactions were carried out in wells of 384-well Optiplate (PerkinElmer). The serial dilution of the compounds was first performed in 100% DMSO with the highest concentration at 1 mM. Each intermediate compound dilution (in 100% DMSO) will then get directly diluted 30x fold into assay buffer for 3.3x conc (DMSO). Enzyme only and blank only wells have a final DMSO concentration of 1%. From this intermediate step, 3 µl of compound is added to 4 µl of demethylase enzyme dilution is incubated for 30 minutes at room temperature. After this incubation, 3 µl of peptide substrate is added. The final DMSO concentration is 1%.

After enzymatic reactions, 5 µl of anti-Mouse Acceptor beads (PerkinElmer, diluted 1:500 with 1x detection buffer) or 5 µl of anti-Rabbit Acceptor beads (PerkinElmer, diluted 1:500 with 1x detection buffer) and 5 µl of Primary antibody (BPS, diluted 1:200 with 1x detection buffer) were added to the reaction mix. After brief shaking, plate was incubated for 30 minutes. Finally, 10 µl of AlphaScreen Streptavidin-conjugated donor beads (Perkin, diluted 1:125 with 1x detection buffer) were added. In 30 minutes, the samples were measured in AlphaScreen microplate reader (EnSpire Alpha 2390 Multilabel Reader, PerkinElmer).

For JMJD3, Donor and acceptor beads and primary antibody were incubated for 1 h,[9] for the remaining enzymes for 30'.

5 µL of AlphaScreen assay buffer containing EDTA (30 mM) and NaCl (800 mM) to stop the JMJD3 reactions.[9]

Except for JMJD3, the experiments were performed by BPS Bioscience.

Data analysis

Enzyme activity assays were performed in duplicates at each concentration. The A-screen intensity data were analyzed and compared. In the absence of the compound, the intensity in each data set was defined as 100% activity. In the absence of enzyme, the intensity in each data set was defined as 0% activity. The values of % activity versus a series of compound concentrations were then plotted using non-linear regression analysis of Sigmoidal dose-response curve generated with the equation Y=B+(T-B)/1+10((LogEC_{50}-X)×Hill Slope), where Y=percent activity, B=minimum percent activity, T=maximum percent activity, X= logarithm of compound and Hill Slope=slope factor or Hill coefficient. The IC_{50} value was determined by the concentration causing a half-maximal percent activity.

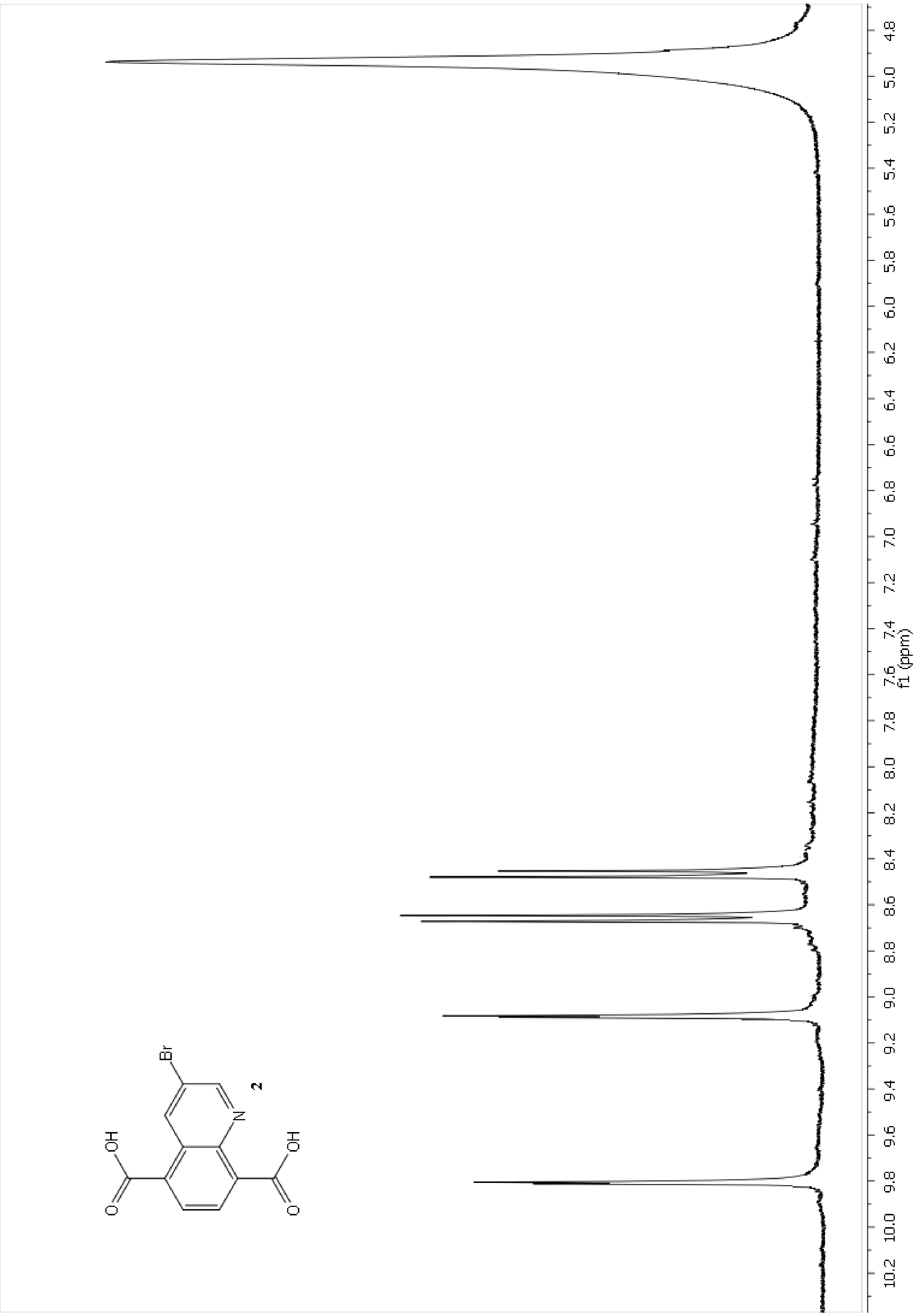

Figure S23. Compound **2** ^{1}H NMR (CD_3OD, 300 MHz).

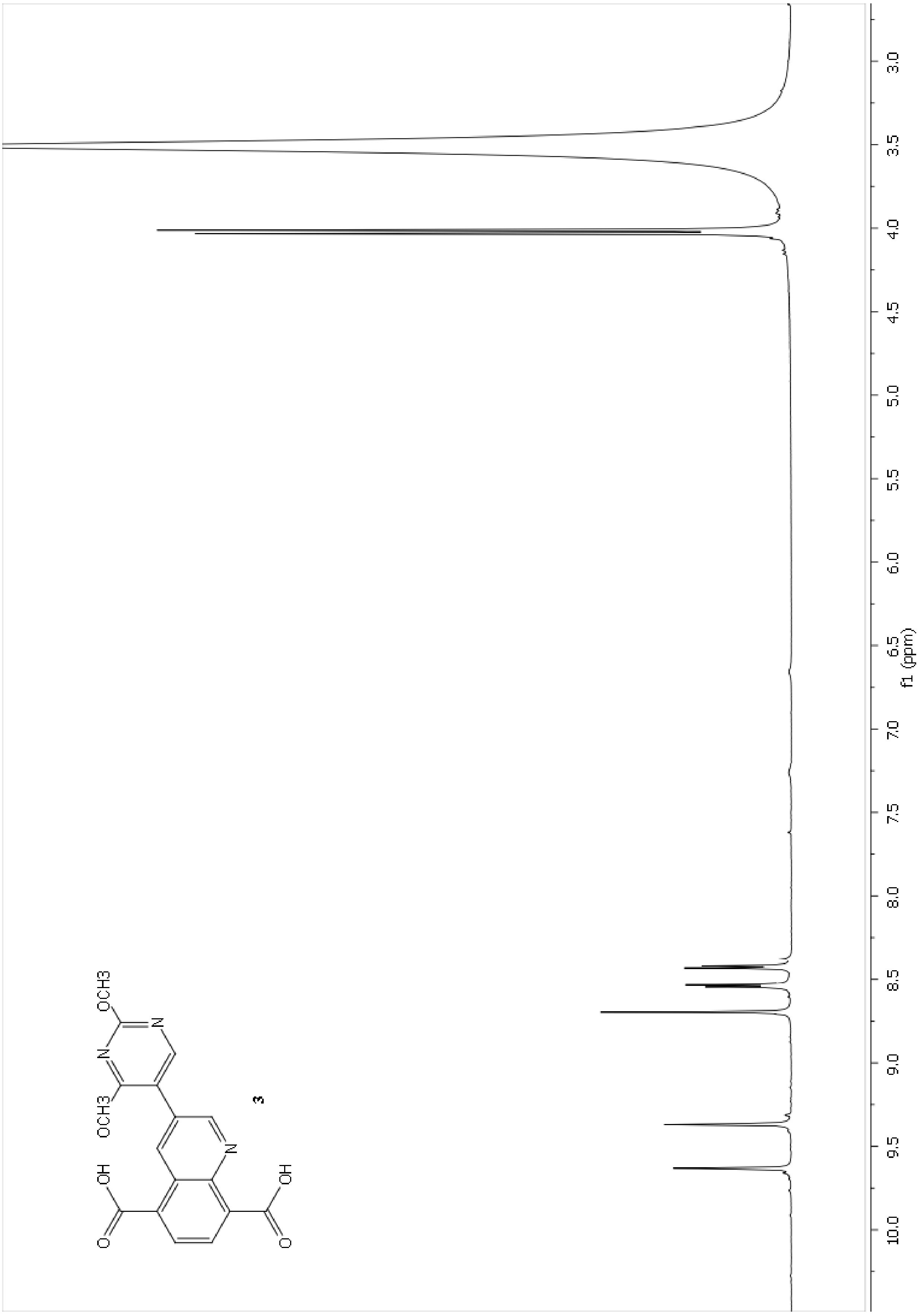

Figure S24. Compound **3** ^{1}H NMR (DMSO-d_6, 600 MHz).

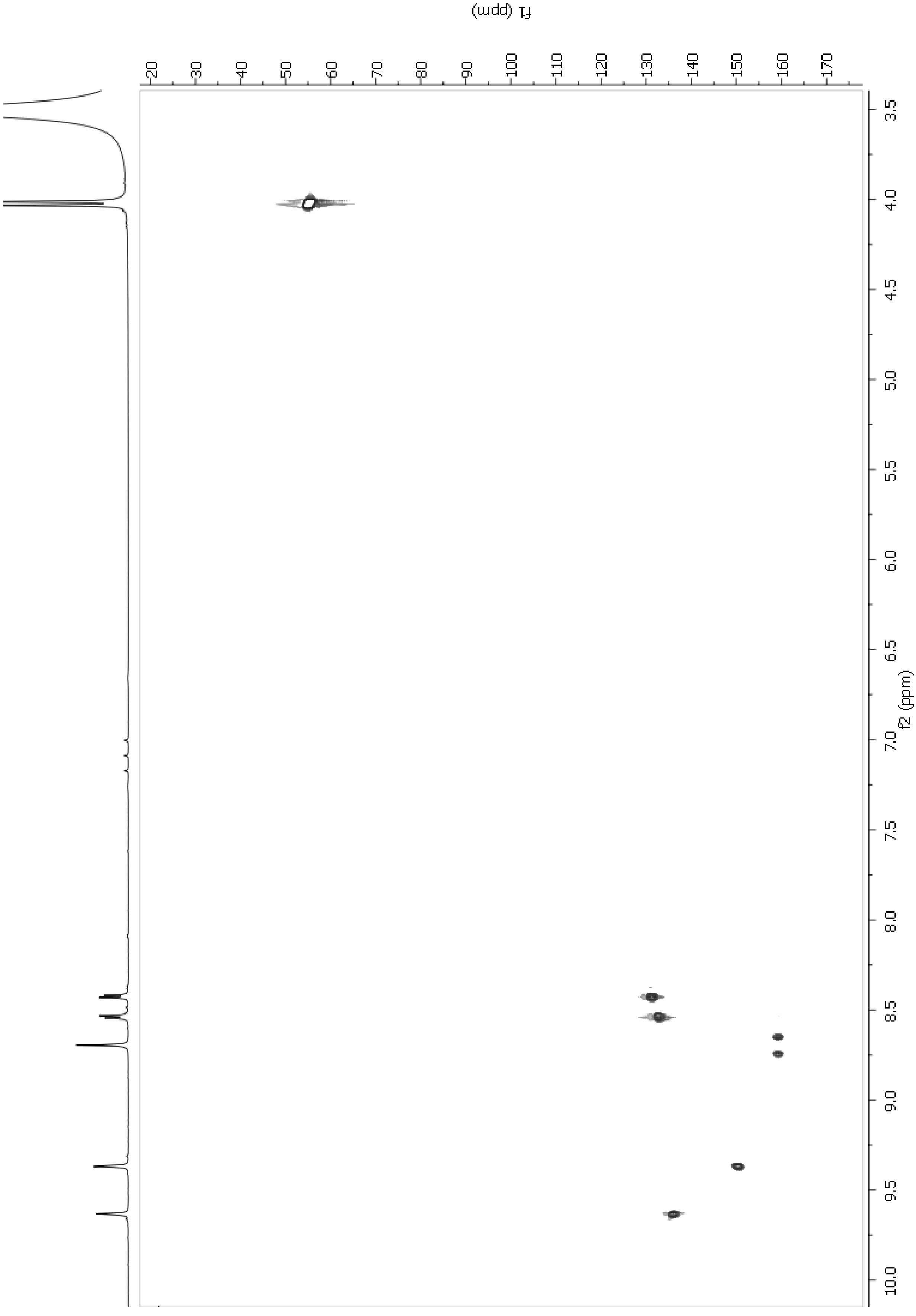

Figure S25. Compound **3** HSQC (DMSO-d_6, 600 MHz).

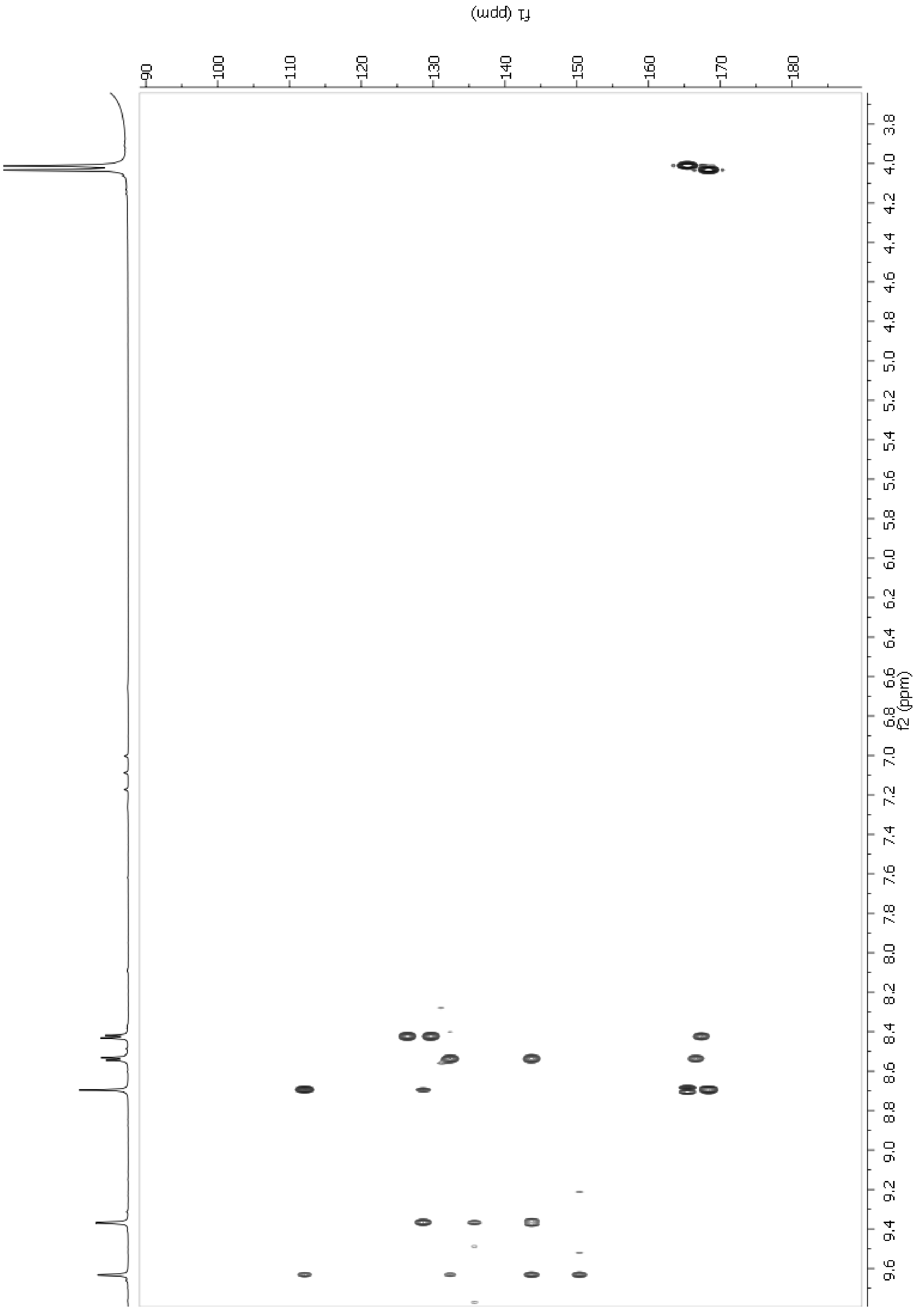

Figure S26. Compound **3** HMBC (DMSO-d$_6$, 600 MHz).

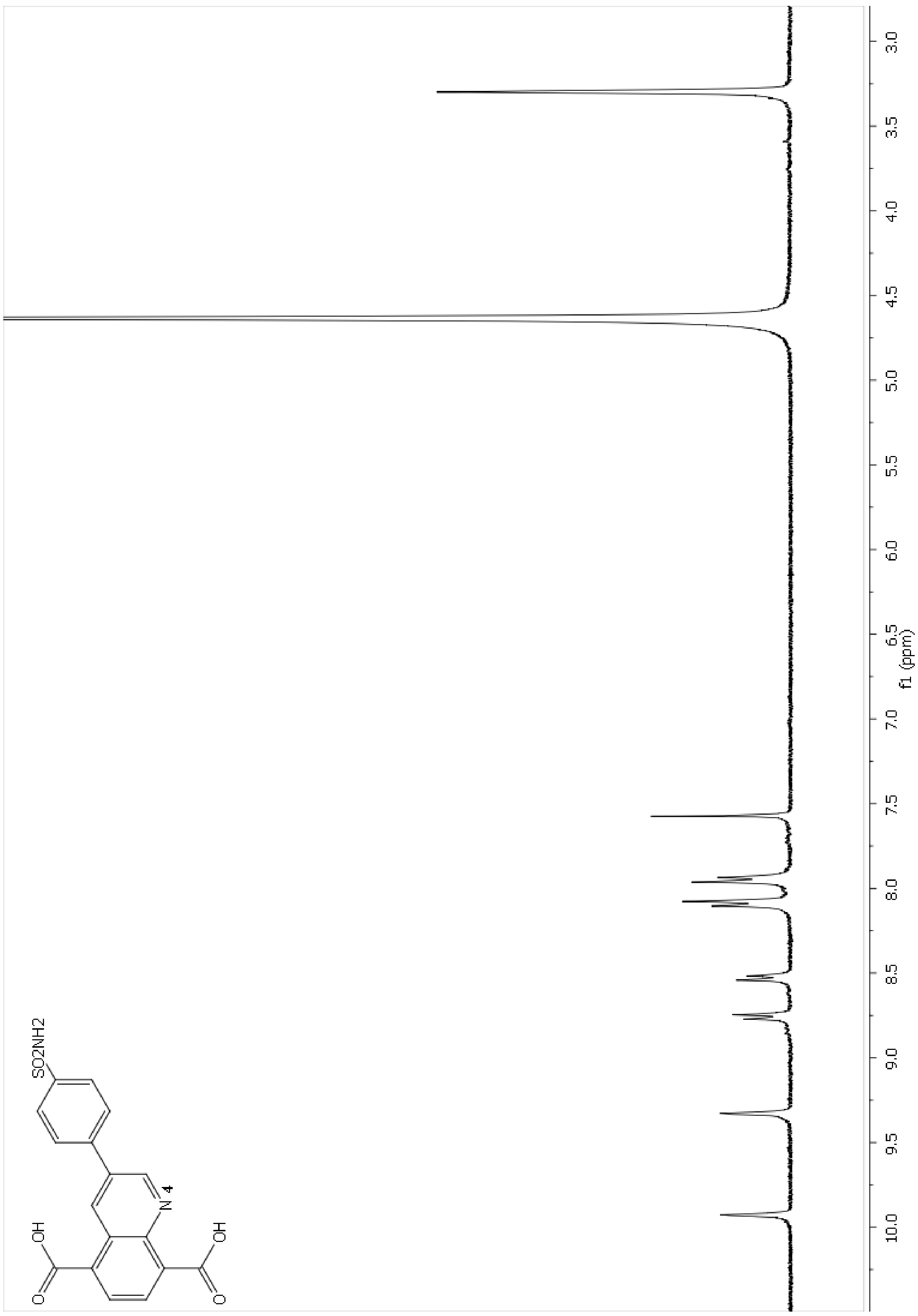

Figure S27. Compound **4** ^{1}H NMR ($CDCl_3/CD_3OD$ 1:1, 300 MHz).

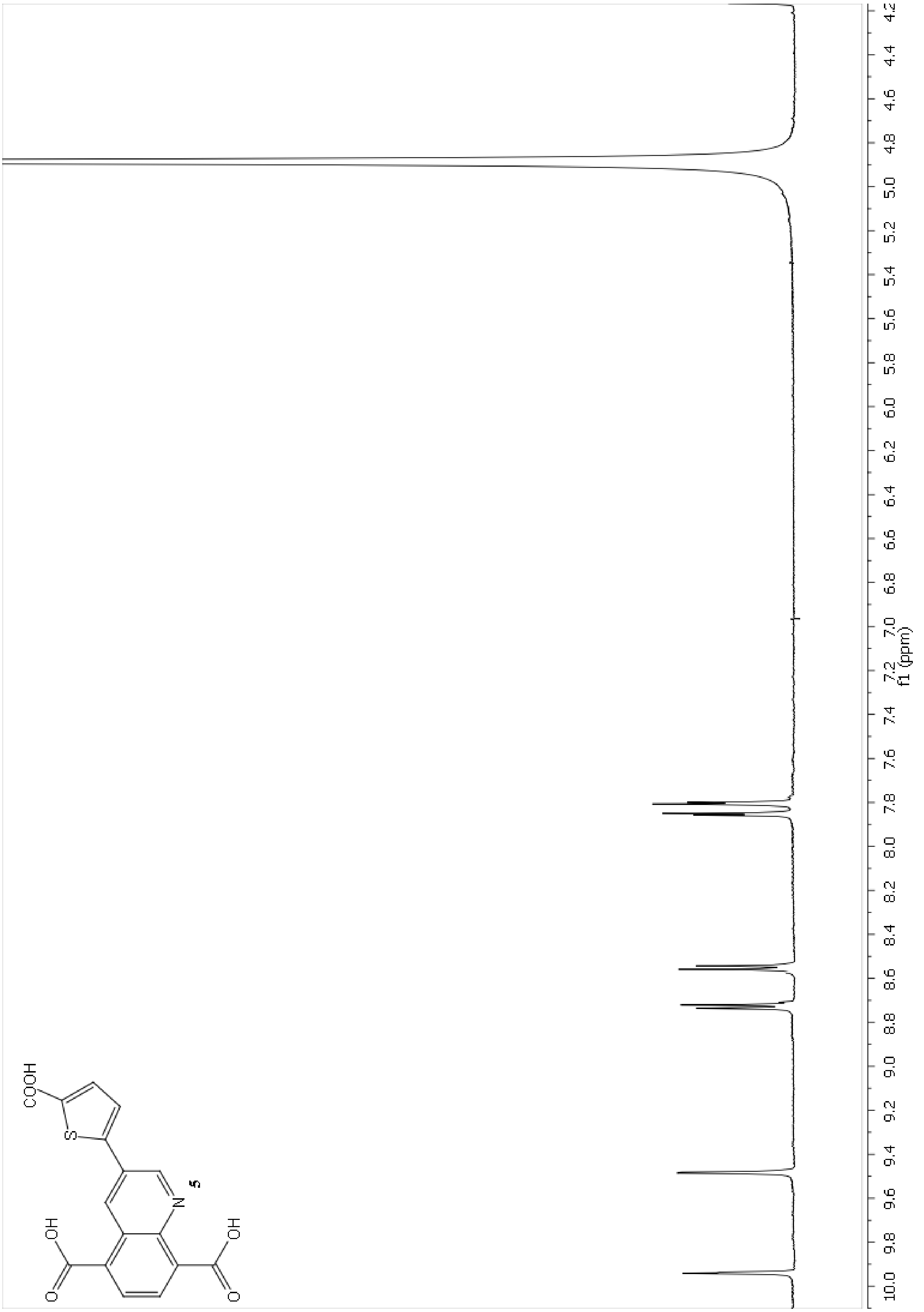

Figure S28. Compound **5** ^{1}H NMR (DMSO-d_6, 500 MHz).

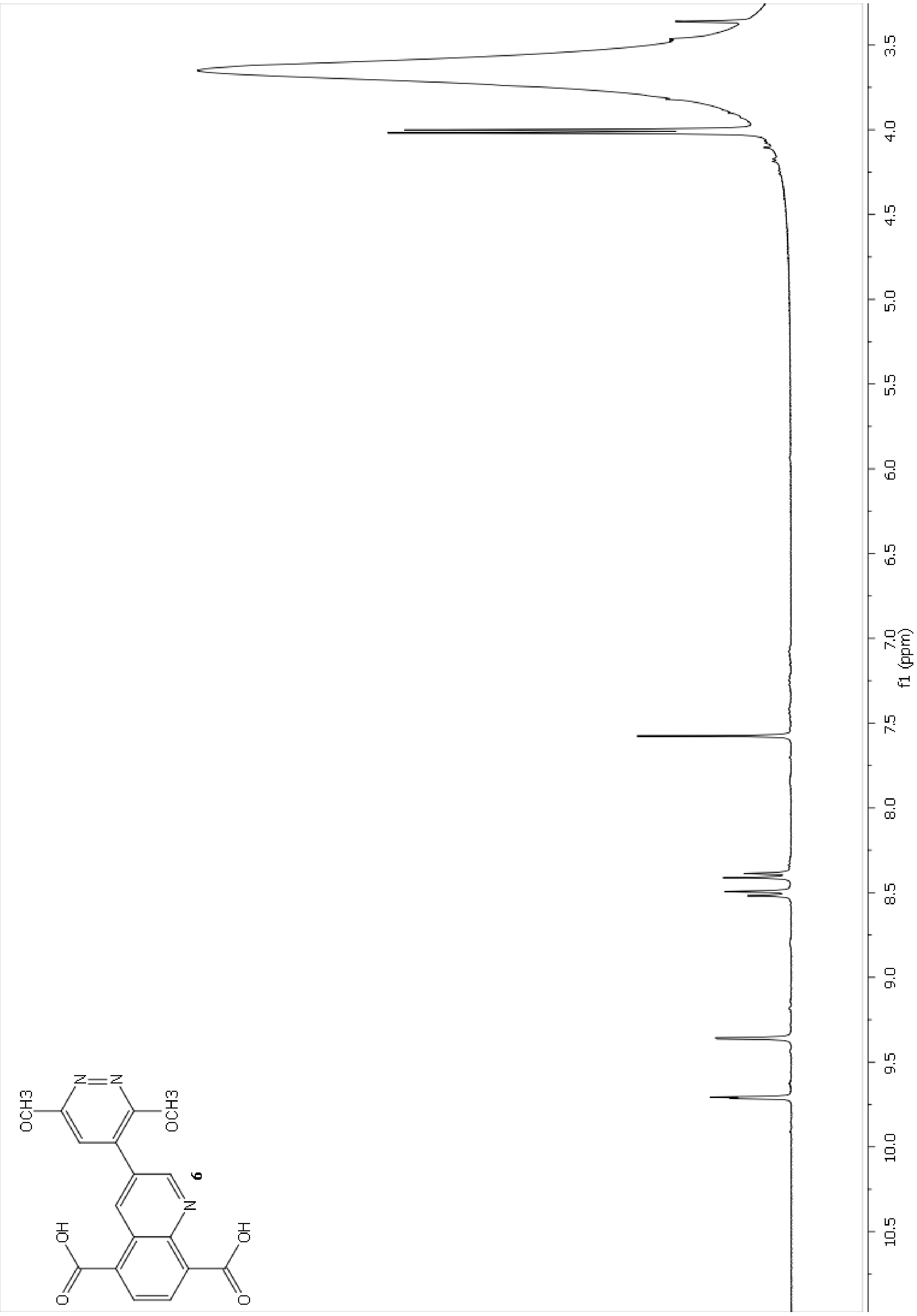

Figure S29. Compound **6** ^{1}H NMR (DMSO-d_6, 300 MHz).

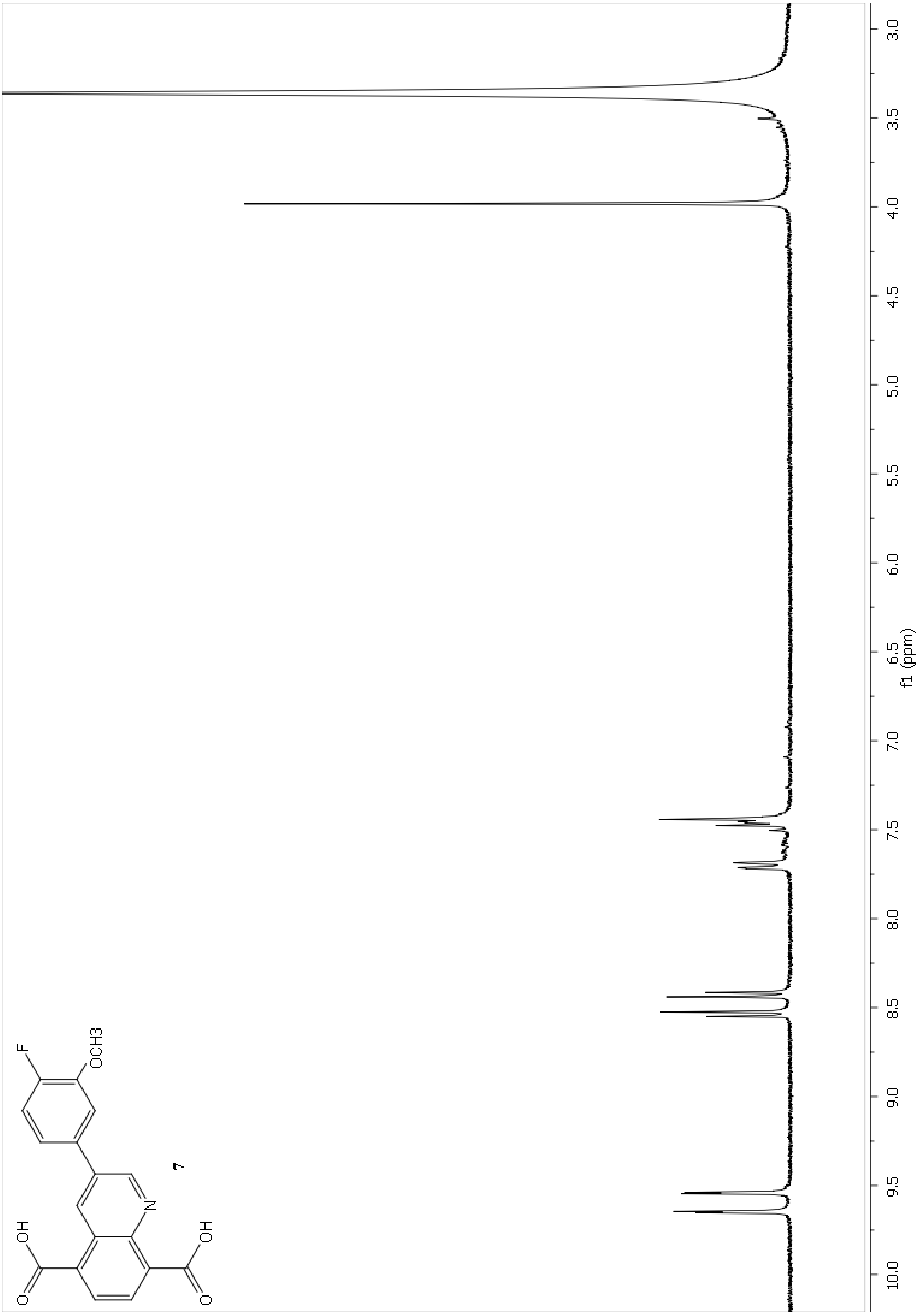

Figure S30. Compound **7** ^{1}H NMR (DMSO-d_6, 300 MHz).

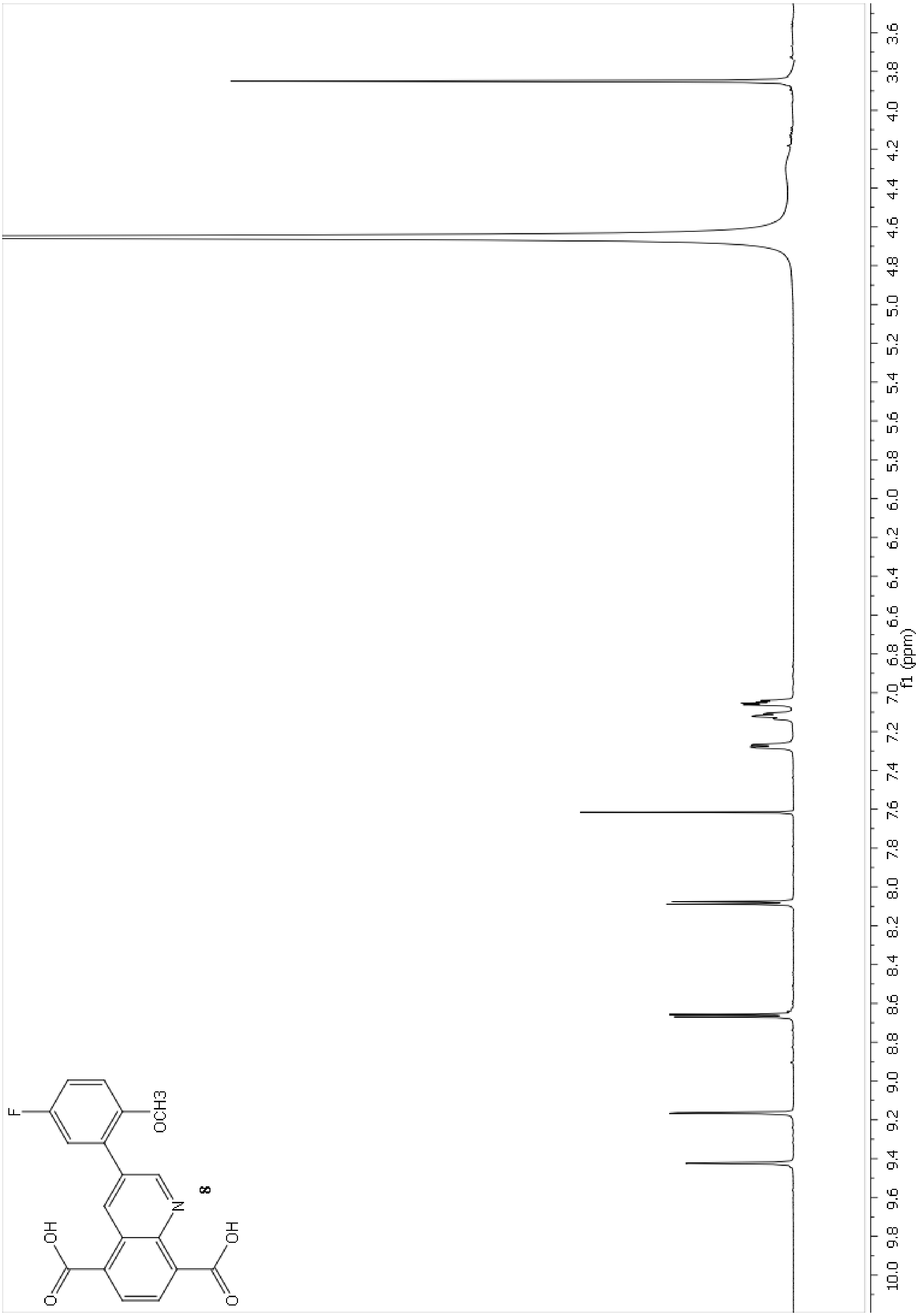

Figure S31. Compound **8** ^{1}H NMR ($CDCl_3$/CD_3OD 1:1, 300 MHz).

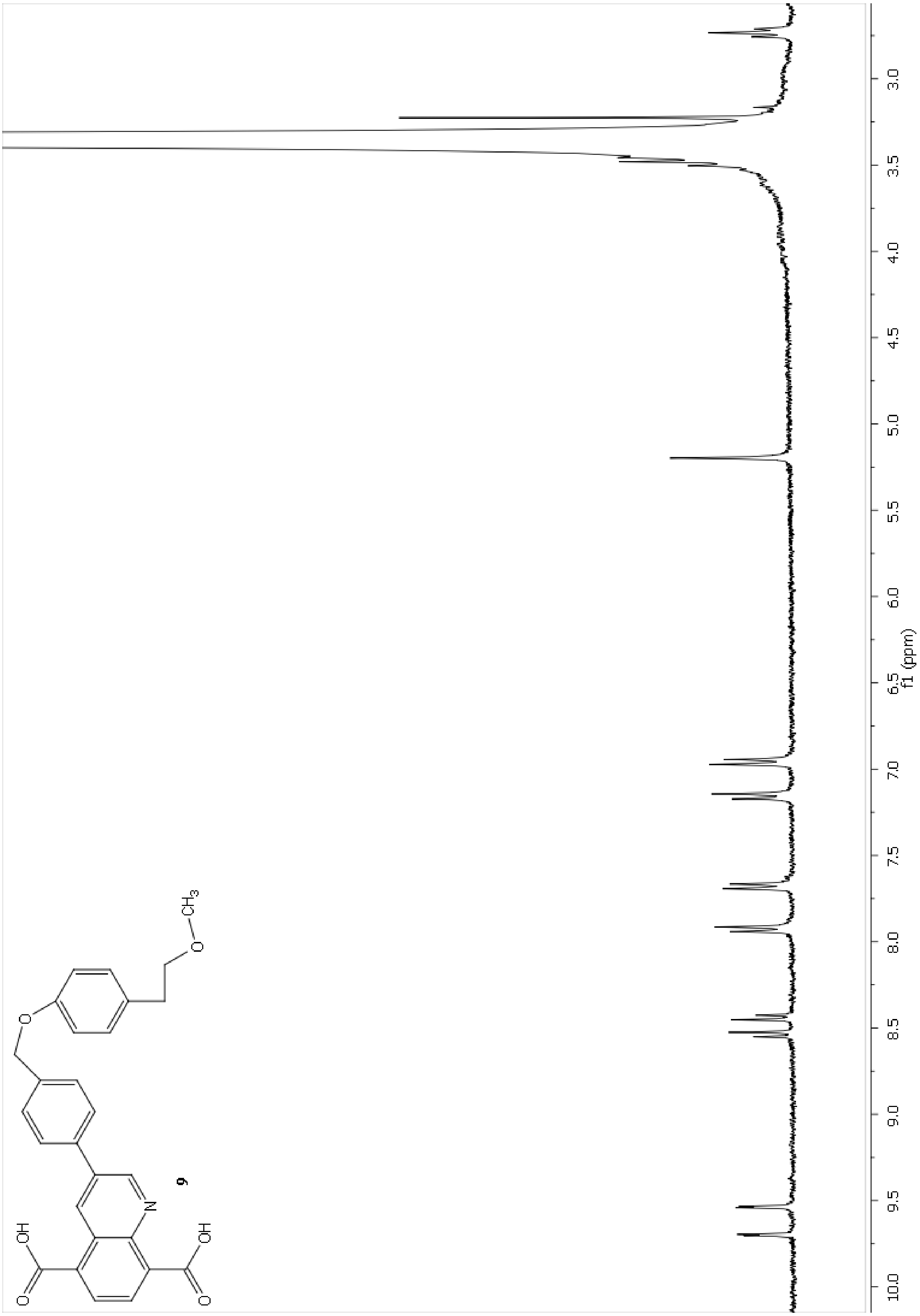

Figure S32. Compound **9** ^{1}H NMR (DMSO-d_6, 300 MHz).

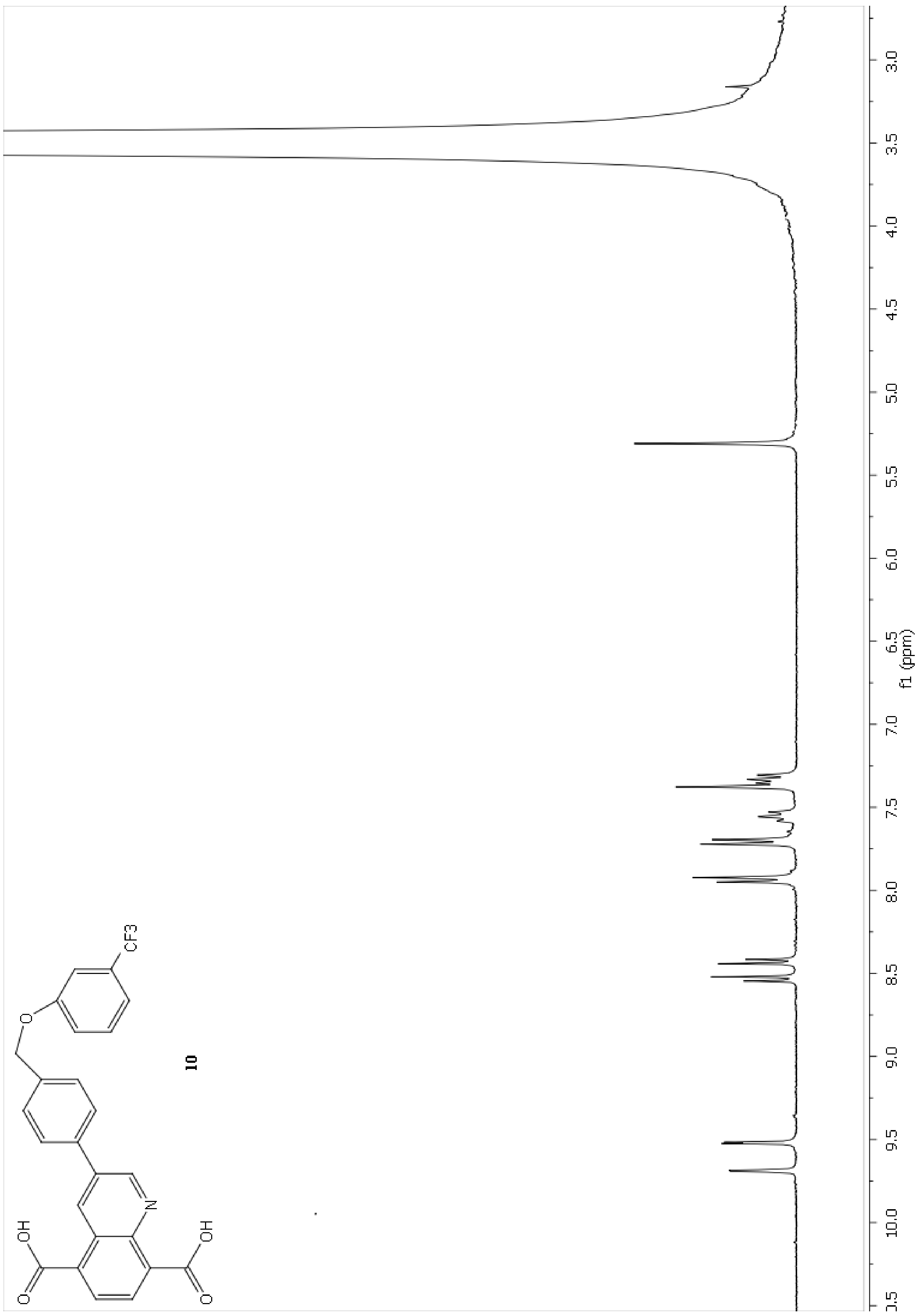

Figure S33. Compound **10** ^{1}H NMR (DMSO-d_6, 300 MHz).

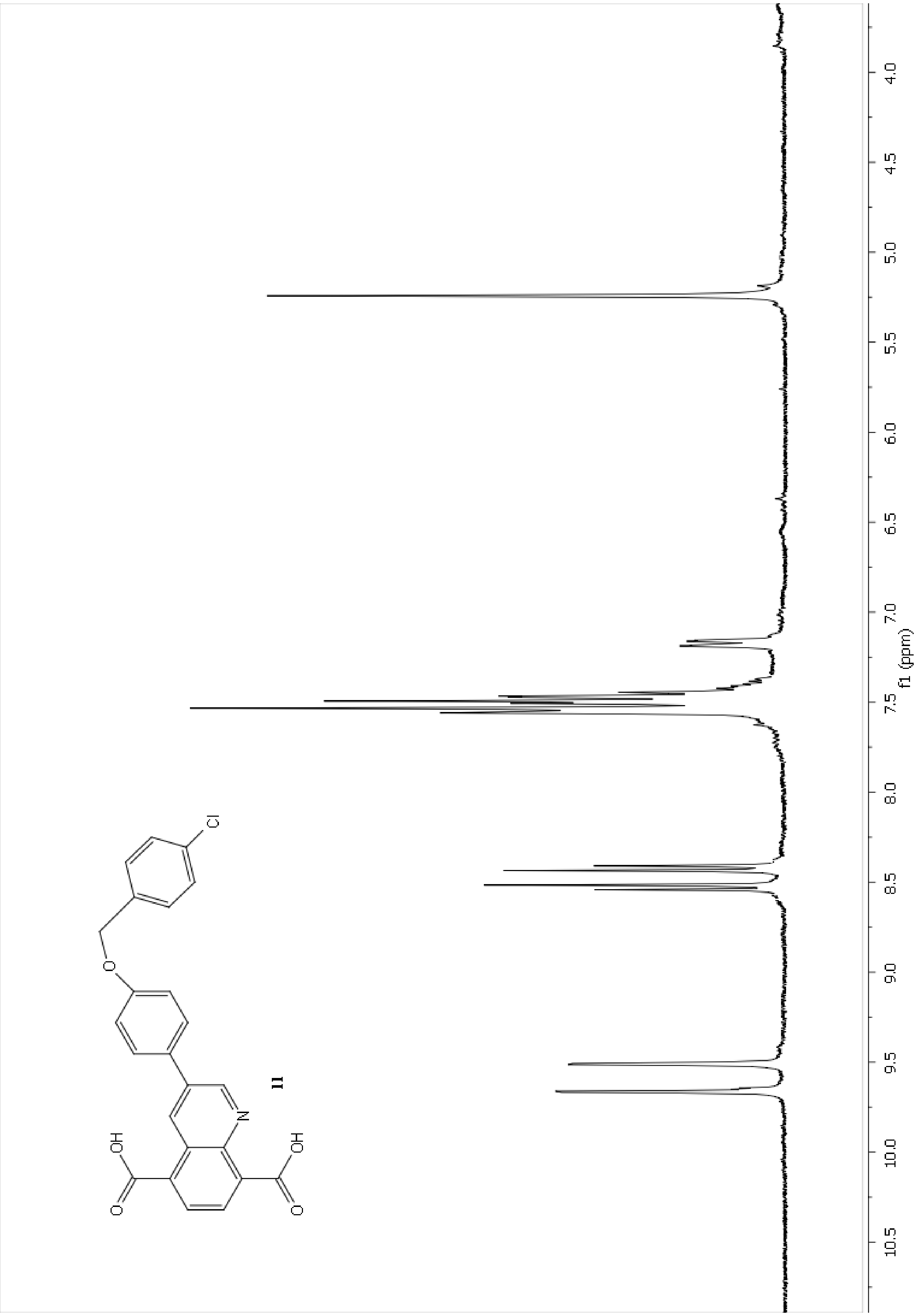

Figure S34. Compound **11** ^{1}H NMR (DMSO-d_6, 300 MHz).

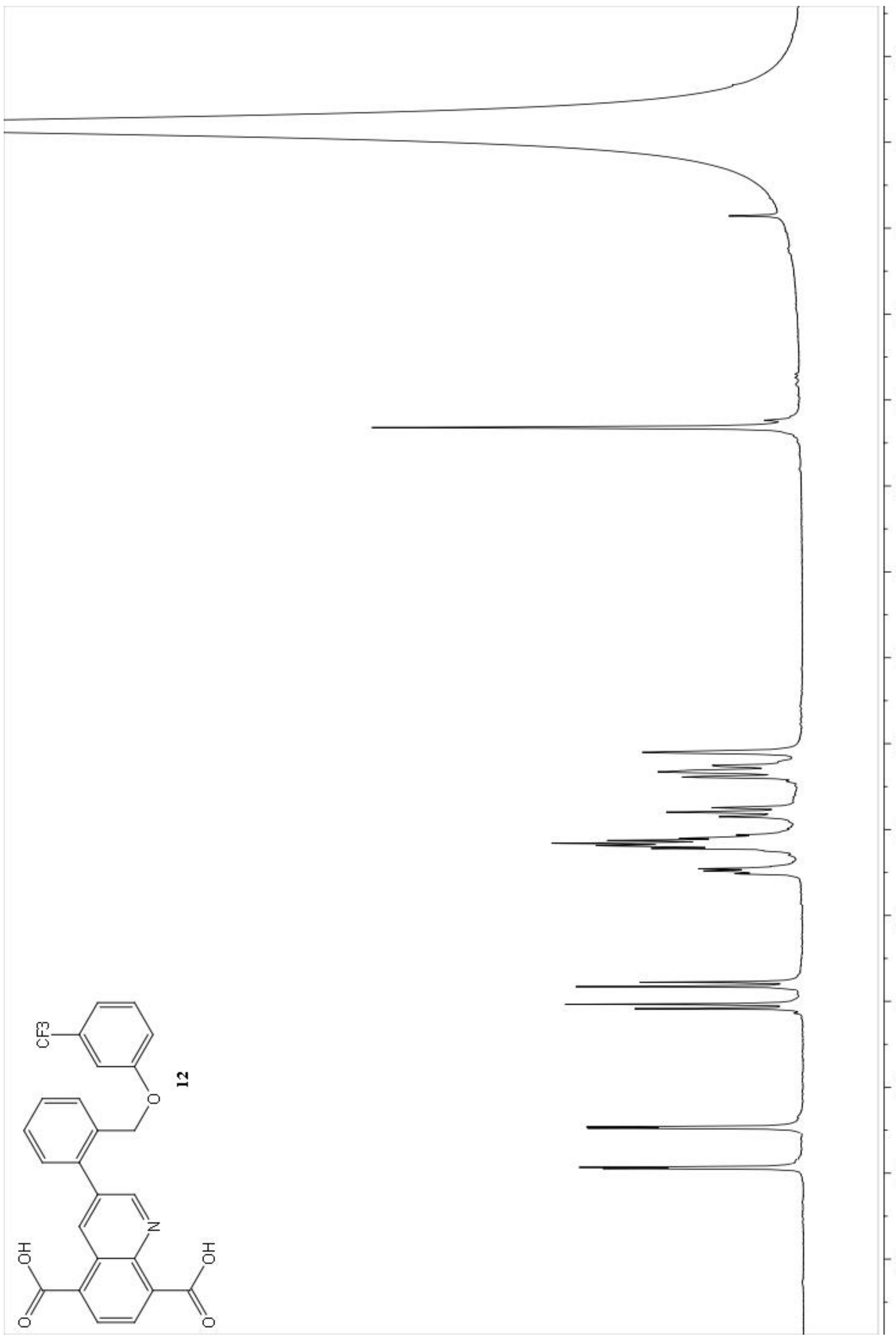

Figure S35. Compound **12** ^{1}H NMR (DMSO-d_6, 300 MHz).

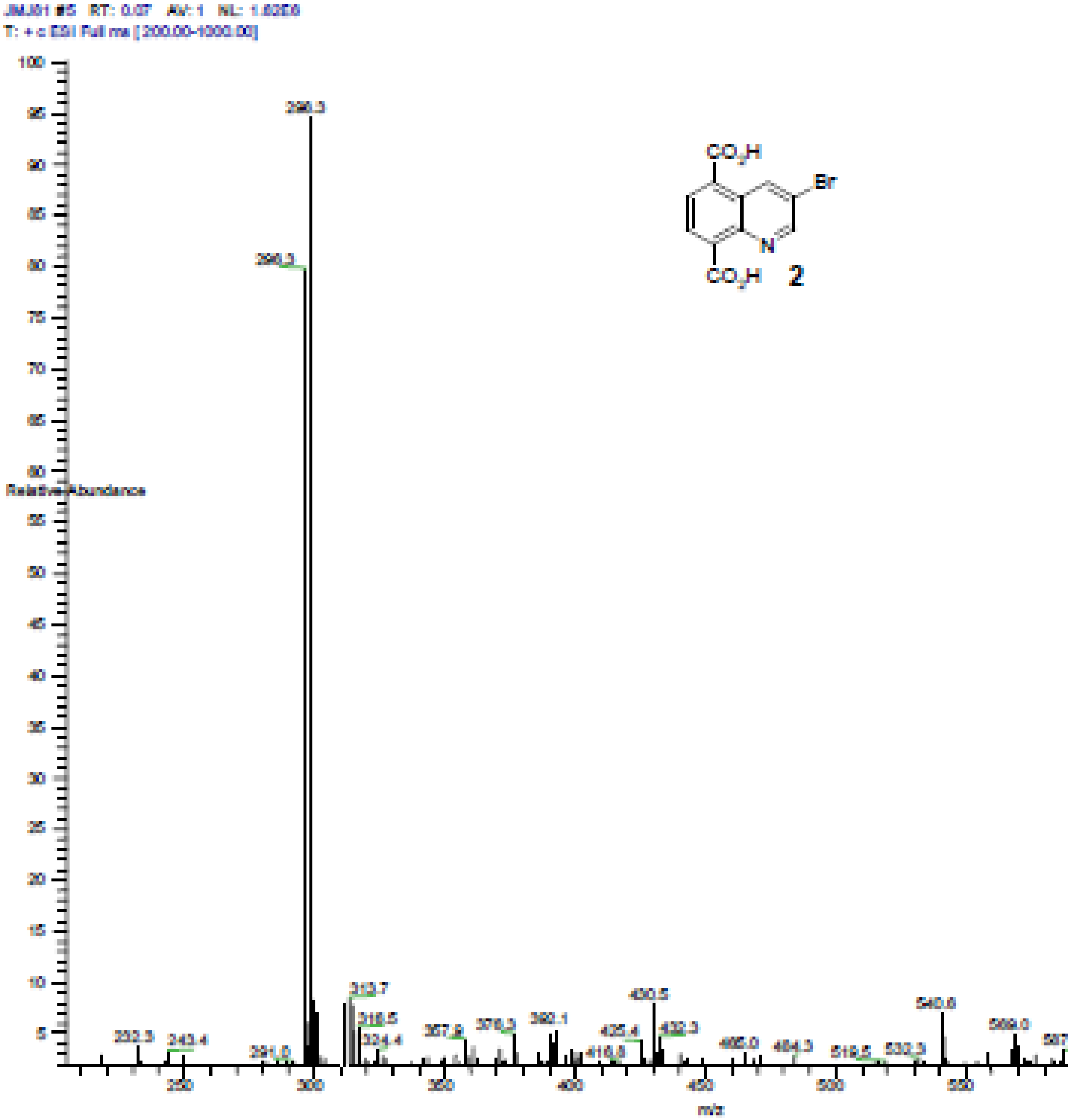

Figure S36. ESI-MS of **2**.

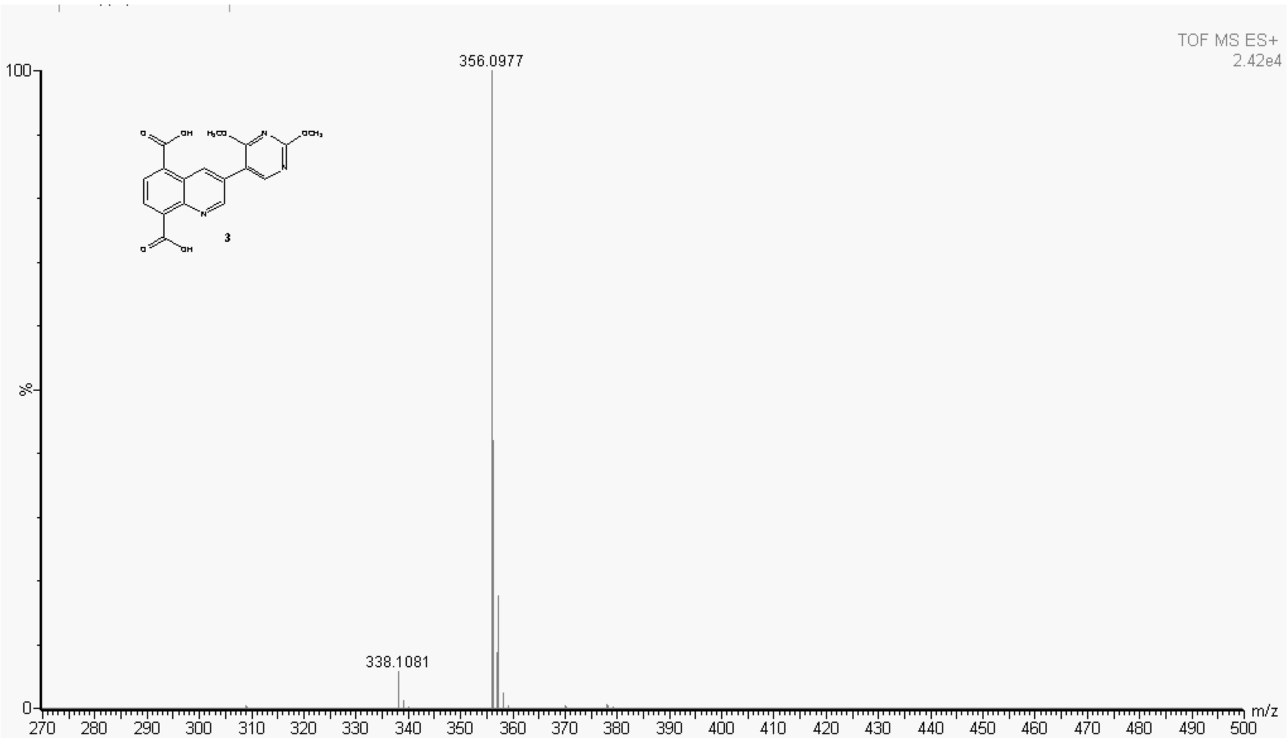

Figure S37. HRMS of **3**.

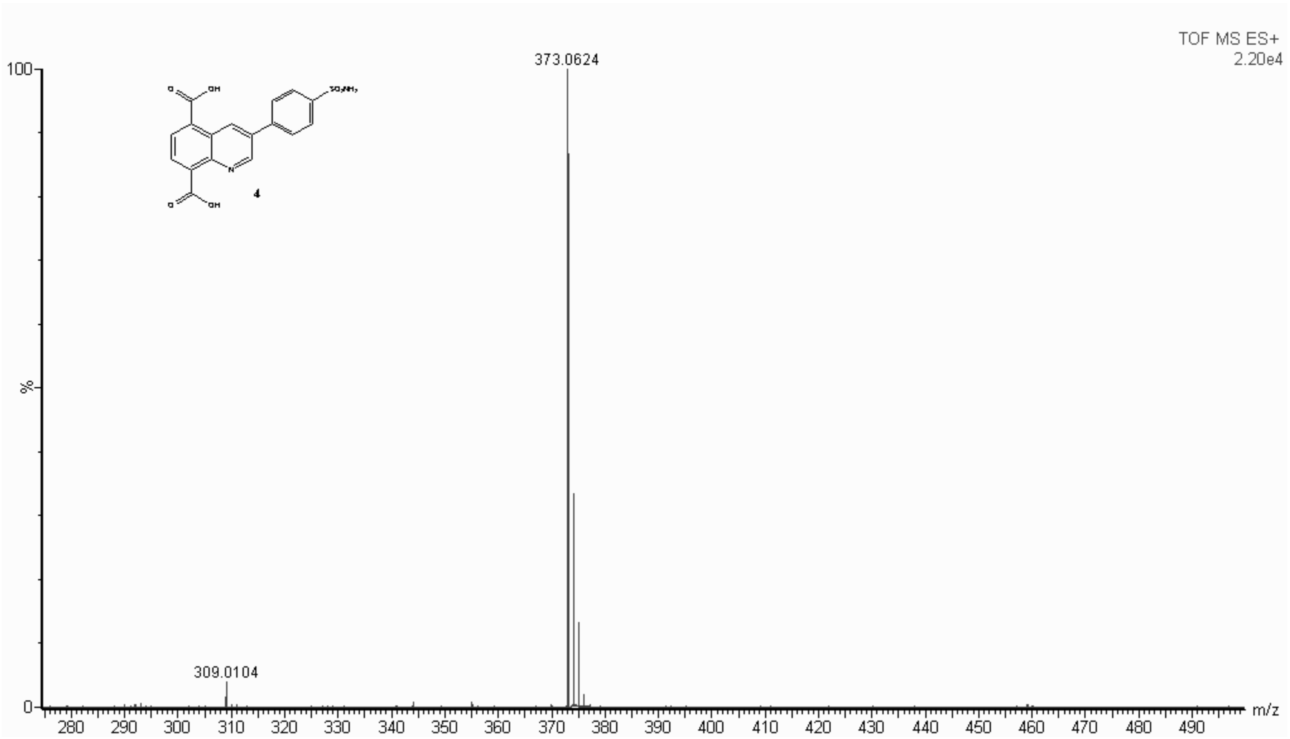

Figure S38. HRMS of **4**.

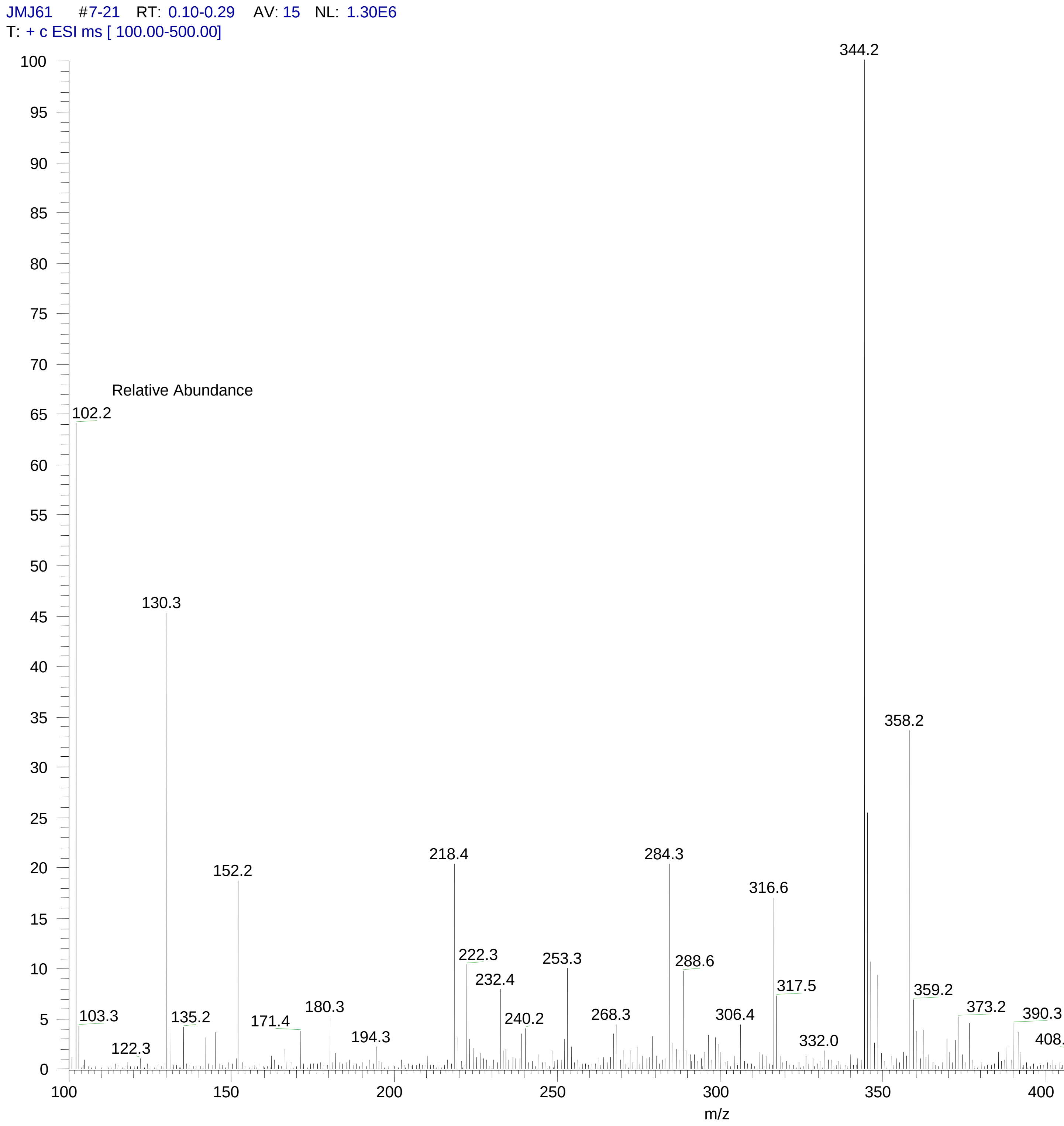

Figure S39. ESI-MS of **5**.

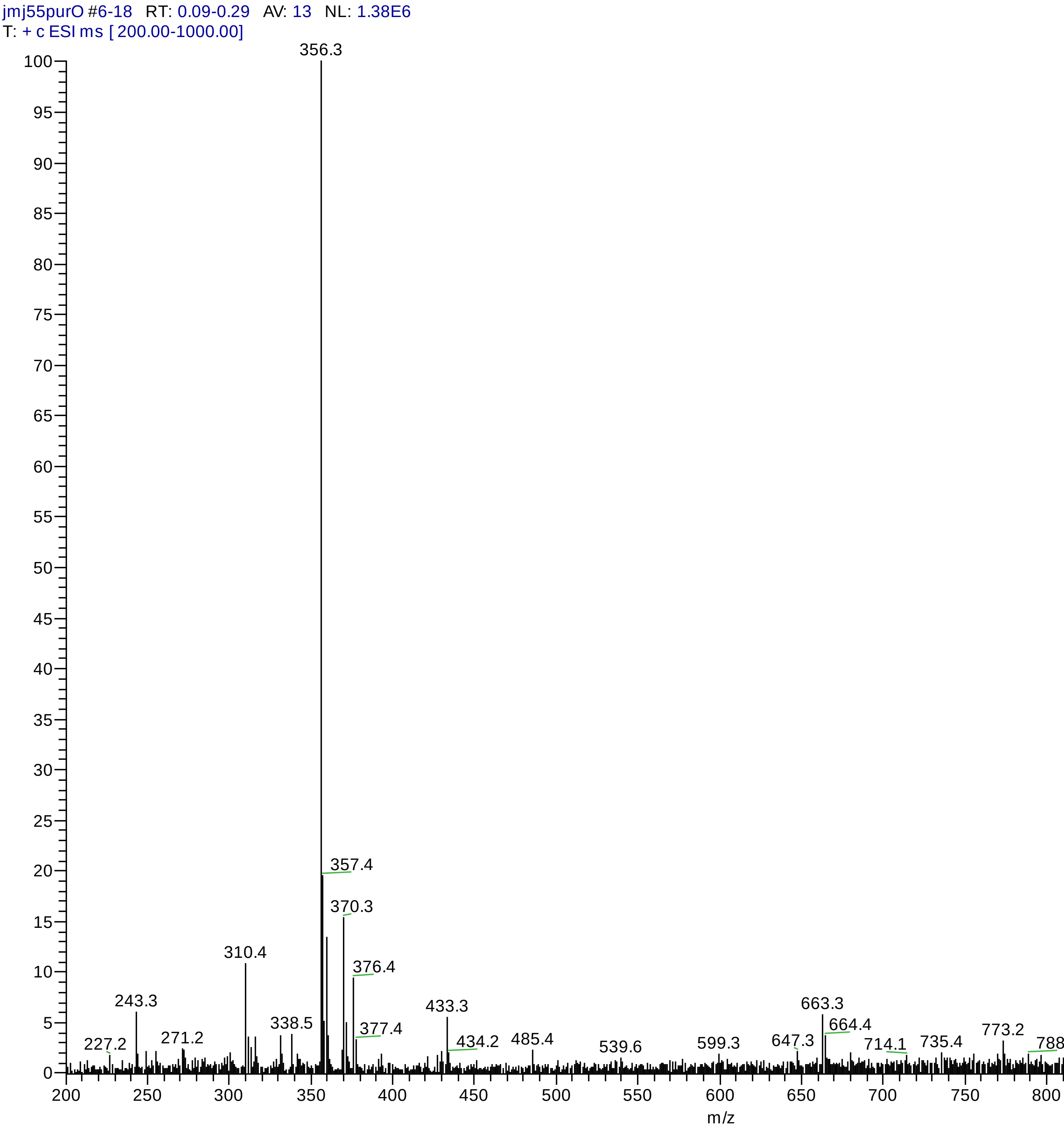

Figure S40. ESI-MS of **6**.

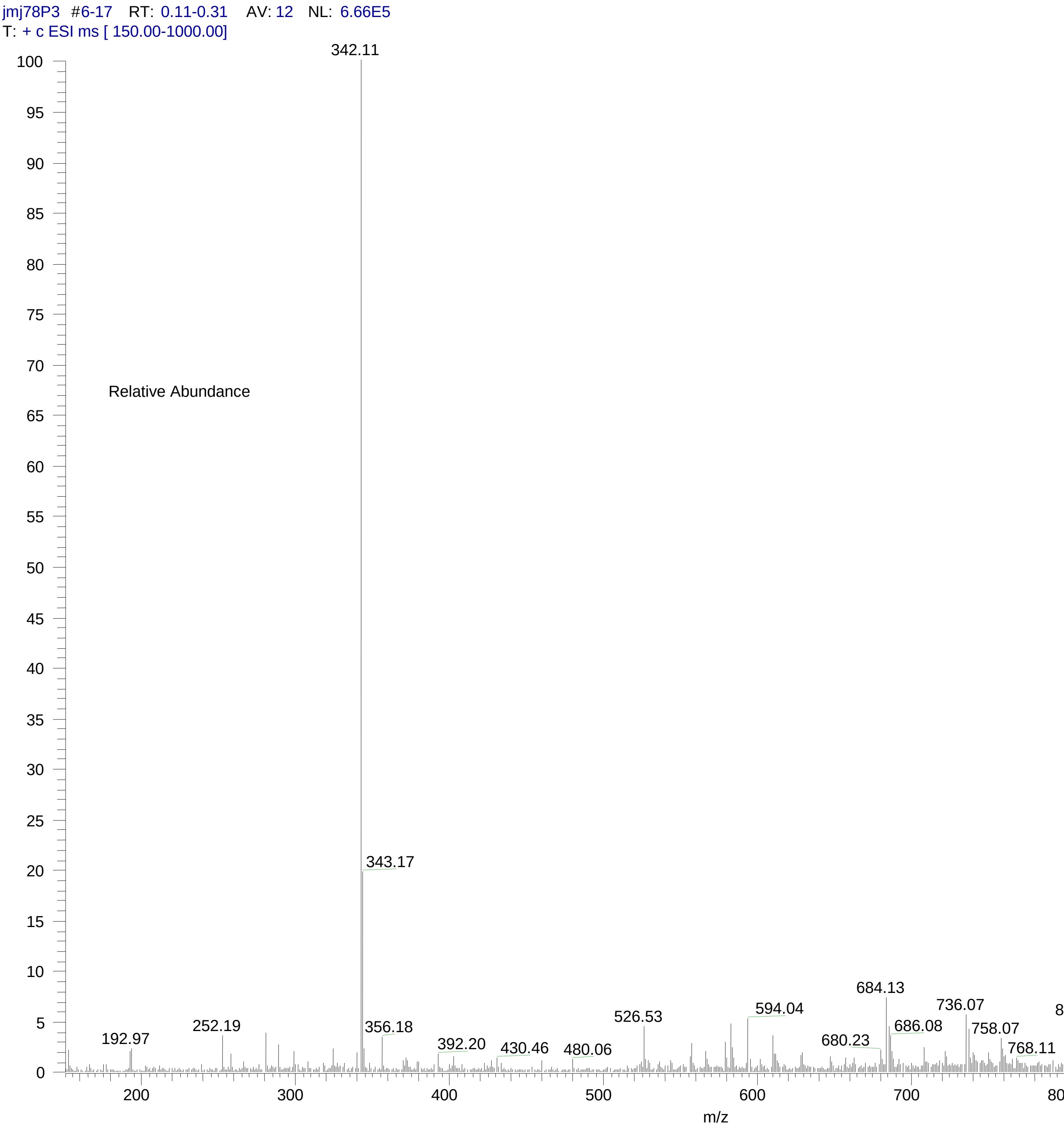

Figure S41. ESI-MS of **7**.

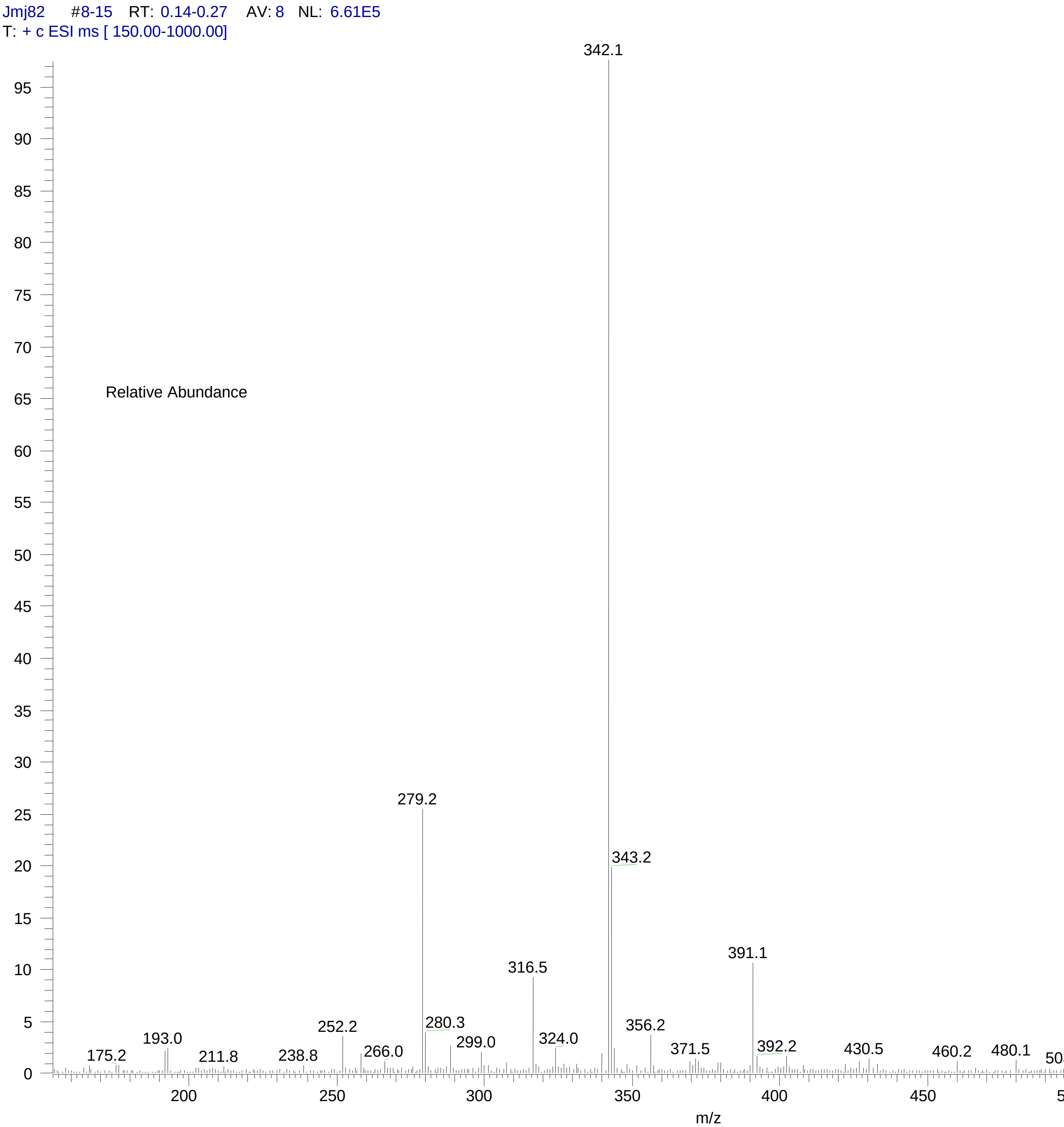

Figure S42. ESI-MS of **8**.

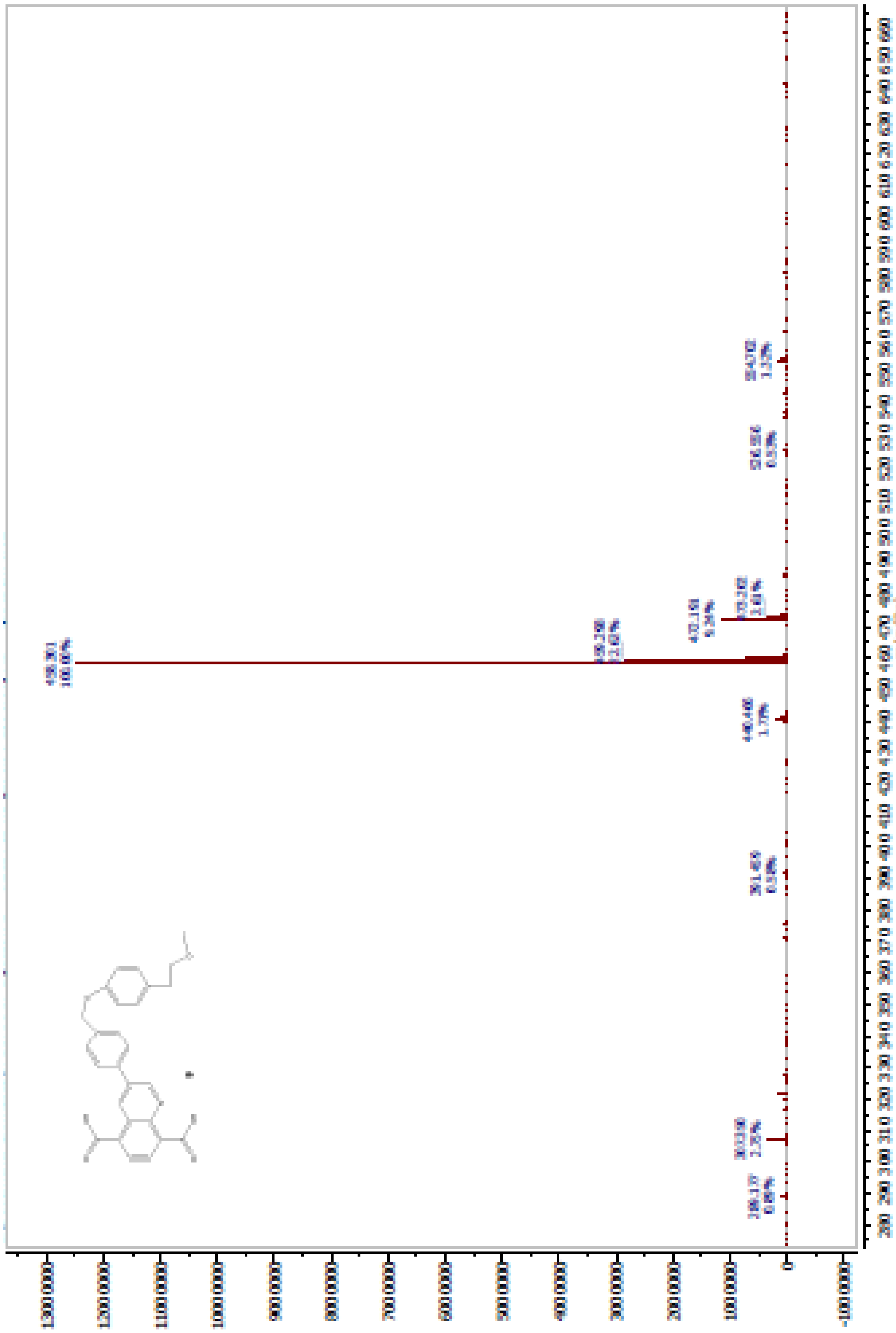

Figure S43. ESI-MS of **9**.

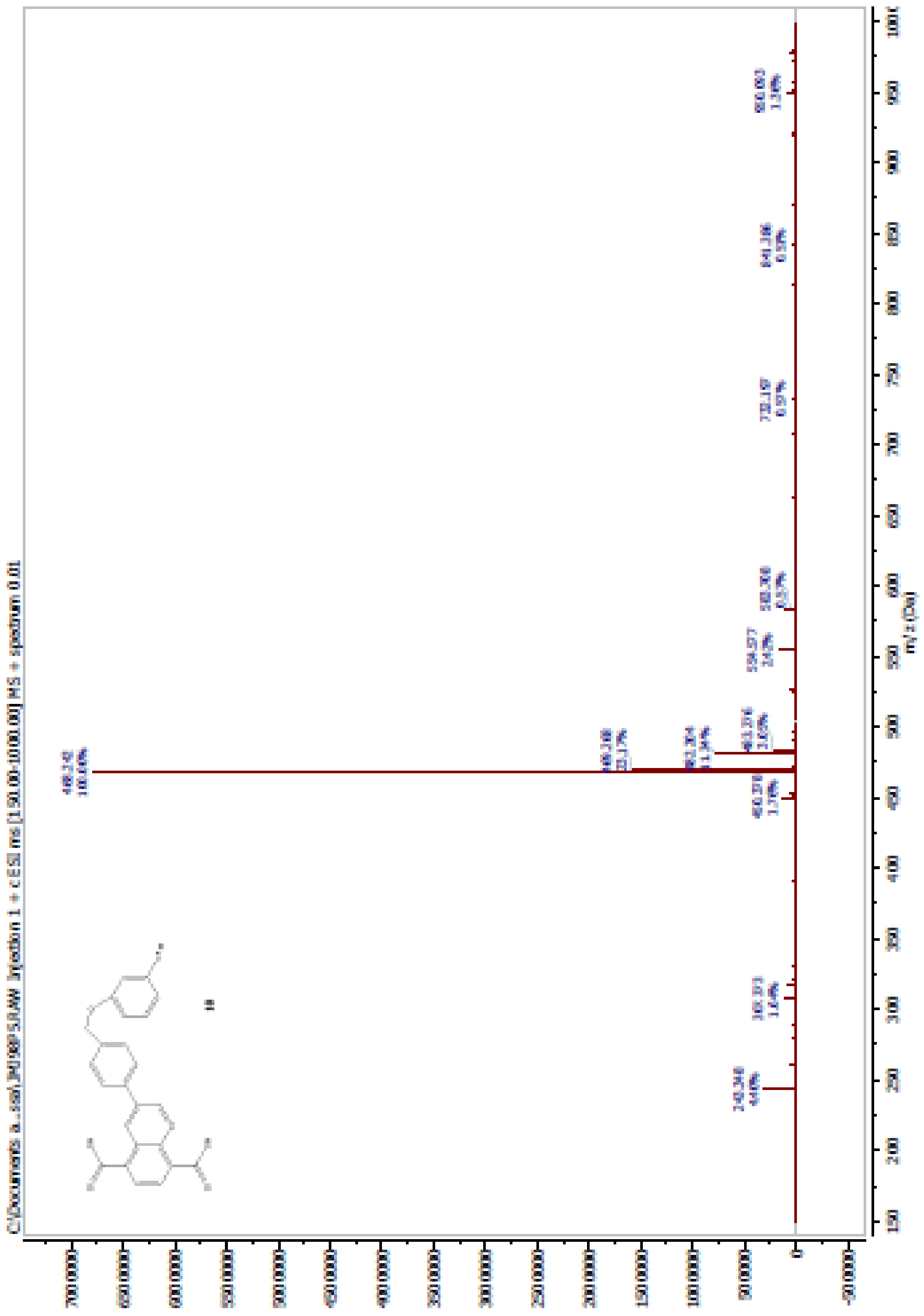

Figure S44. ESI-MS of **10**.

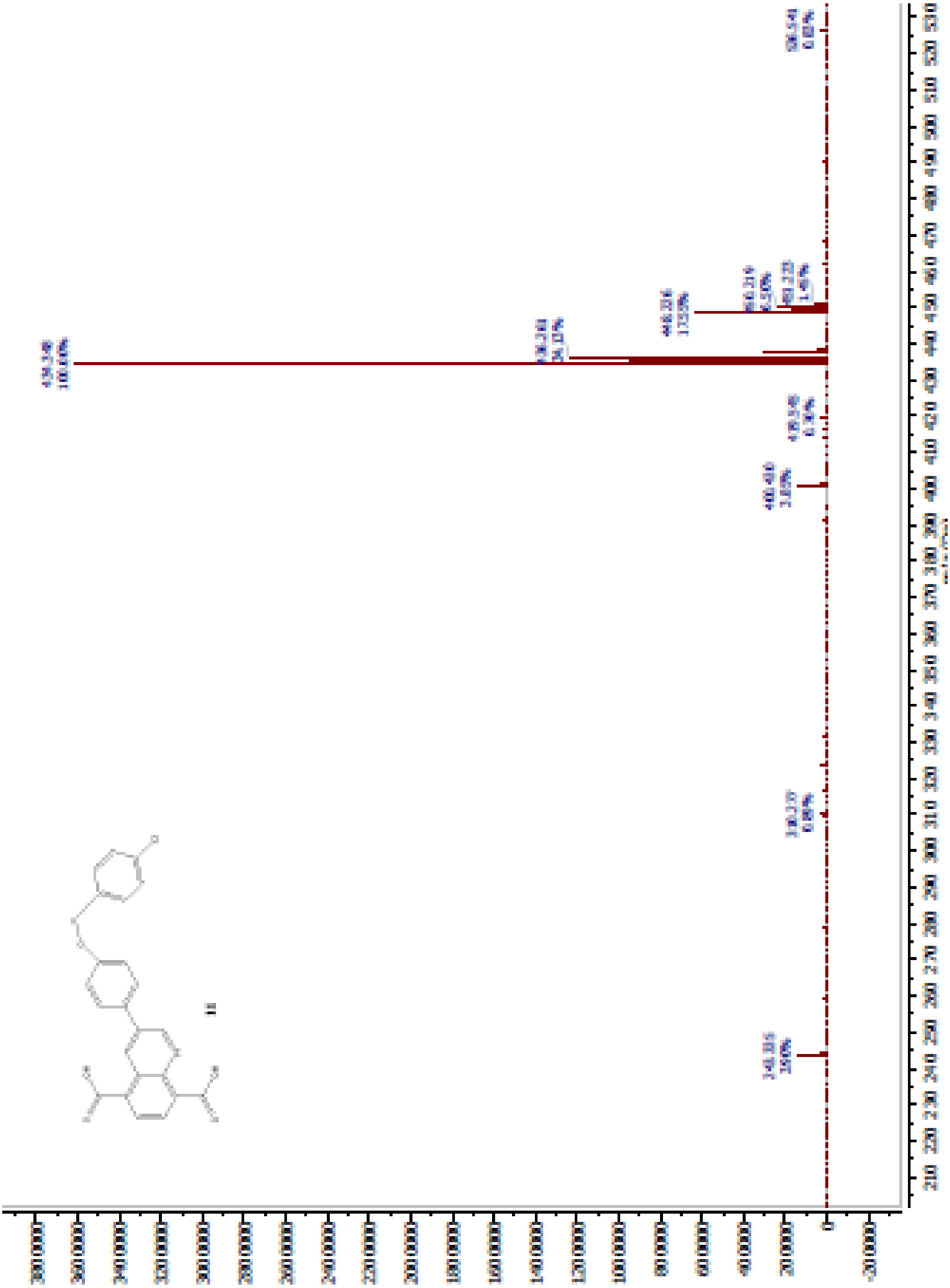

Figure S45. ESI-MS of **11**.

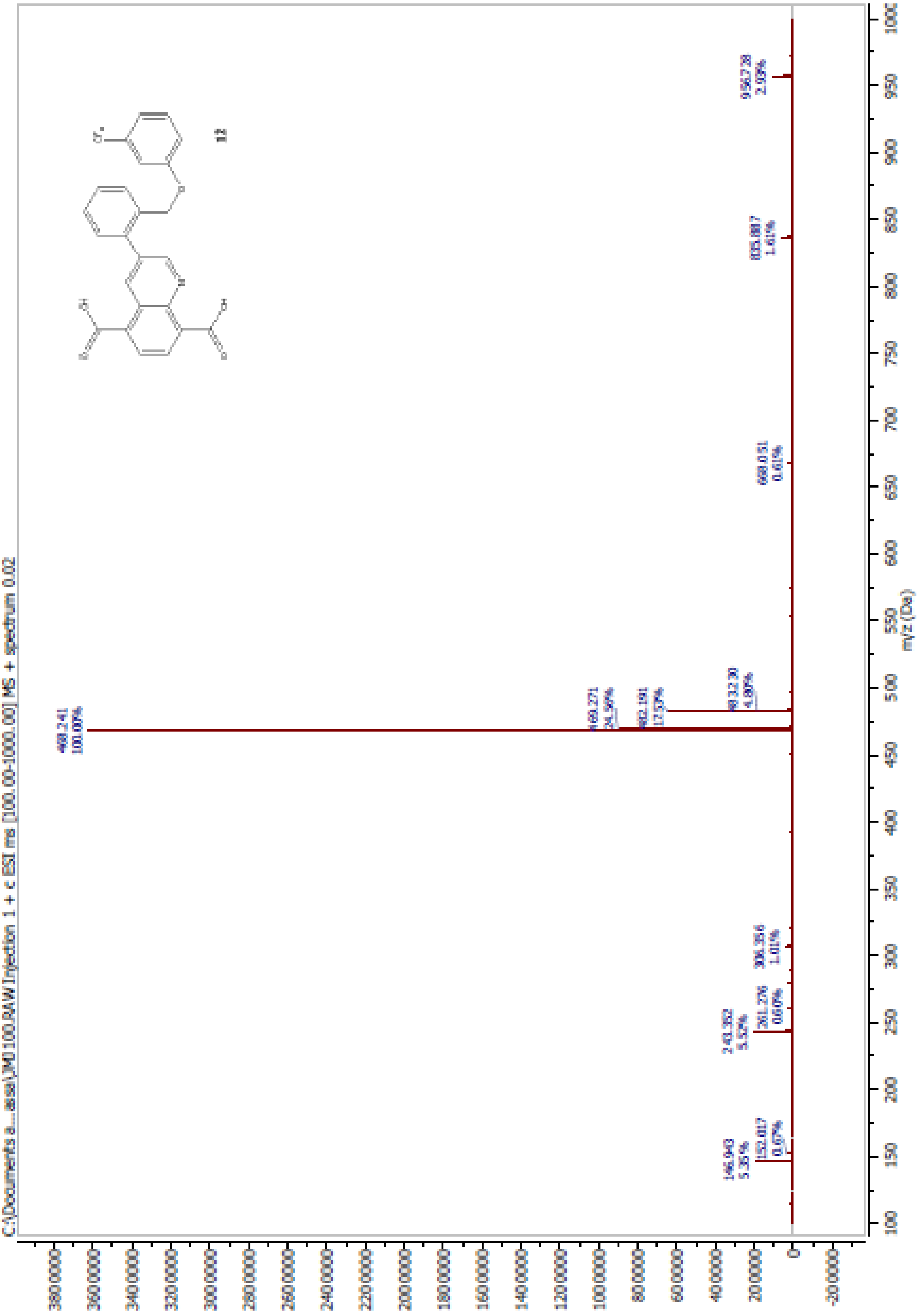

Figure S46. ESI-MS of **12**.

References

[1] Y. Zhao, N. E. Schultz, D. G. Truhlar, *J. Chem. Phys.*, 2005, **123**, 161103-1.
[2] C. M. Breneman, K. B. Wiberg, *J. Comput. Chem.*, 1990, **11**, 361.
[3] M. J. Frisch, G. W. Trucks, H. B. Schlegel, G. E. Scuseria, M. A. Robb, J. R. Cheeseman, G. Scalmani, V. Barone, B. Mennucci, G. A. Petersson, H. Nakatsuji, M. Caricato, X. Li, H. P. Hratchian, A. F. Izmaylov, J. Bloino, G. Zheng, J. L. Sonnenberg, M. Hada, M. Ehara, K. Toyota, R. Fukuda, J. Hasegawa, M. Ishida, T. Nakajima, Y. Honda, O. Kitao, H. Nakai, T. Vreven, J. A. Montgomery, Jr., J. E. Peralta, F. Ogliaro, M. Bearpark, J. J. Heyd, E. Brothers, K. N. Kudin, V. N. Staroverov, R. Kobayashi, J. Normand, K. Raghavachari, A. Rendell, J. C. Burant, S. S. Iyengar, J. Tomasi, M. Cossi, N. Rega, J. M. Millam, M. Klene, J. E. Knox, J. B. Cross, V. Bakken, C. Adamo, J. Jaramillo, R. Gomperts, R. E. Stratmann, O. Yazyev, A. J. Austin, R. Cammi, C. Pomelli, J. W. Ochterski, R. L. Martin, K. Morokuma, V. G. Zakrzewski, G. A. Voth, P. Salvador, J. J. Dannenberg, S. Dapprich, A. D. Daniels, Ö. Farkas, J. B.Foresman, J. V. Ortiz, J. Cioslowski, D. J. Fox, Gaussian 09, Revision A.1, Gaussian, Inc., Wallingford CT, 2009.
[4] Schrödinger Release 2017-3: Maestro, Schrödinger, LLC, New York, NY, 2017.
[5] Schrödinger Release 2017-3: MacroModel, Schrödinger, LLC, New York, NY, 2017.
[6] E. Harder, W. Damm, J. Maple, C. Wu, M. Reboul, J. Y. Xiang, L. Wang, D. Lupyan, M. K. Dahlgren, J. L. Knight, J. W. Kaus, D. Cerutti, G. Krilov, W. L. Jorgensen, R. Abel, R. A. Friesner, *J. Chem. Theory Comput.*, 2016, **12**, 281-296.
[7] W. C. Still, A. Tempczyk, R. C. Hawley, T. Hendrickson, *J. Am. Chem. Soc.*, 1990, **112**, 6127.
[8] Schrödinger Release 2017-3: LigPrep, Schrödinger, LLC, New York, NY, 2017.
[9] L. Kruidenier, C. W. Chung, Z. Cheng, J. Liddle, K. Che, G. Joberty, M. Bantscheff, C. Bountra, A. Bridges, H. Diallo, D. Eberhard, S. Hutchinson, E. Jones, R. Katso, M. Leveridge, P. K. Mander, J. Mosley, C. Ramirez-Molina, P. Rowland, C. J. Schofield, R. J. Sheppard, J. E. Smith, C. Swales, R. Tanner, P. Thomas, A. Tumber, G. Drewes, U. Oppermann, D. J. Patel, K. Lee, D. M. Wilson, *Nature*, 2012, **488**, 404-408.
[10] Schrödinger Release 2017-3: Schrödinger Suite 2017-3 Protein Preparation Wizard; Epik, Schrödinger, LLC, New York, NY, 2017; Impact, Schrödinger, LLC, New York, NY, 2017; Prime, Schrödinger, LLC, New York, NY, 2017.
[11] G. M. Sastry, M. Adzhigirey, T. Day, R. Annabhimoju, W. Sherman, *J. Comput. Aid. Mol. Des.*, 2013, **27**, 221-234.
[12] G. M. Morris, R. Huey, W. Lindstrom, M. F. Sanner, R. K. Belew, D. S. Goodsell, A. J. Olson, *J. Comput. Chem.*, 2009,**30**, 2785-2791.
[13] S. Cosconati, S. Forli, A. L. Perryman, R. Harris, D. S. Goodsell, A. J. Olson, *Expert Opin. Drug Disc.*,2010,**5**, 597-607.
14 S. Forli, A. J. Olson, *J. Med. Chem.*,2012,**55**, 623-638.
[15] M. F. Sanner, *J. Mol. Graph. Model.*, 1999, **17**, 57.
[16] R. Harris, A. J. Olson., and D. S. Goodsell, *Proteins*, 2007, 1506-1517.
[17] S. Forli, R. Huey, M. E. Pique, M. F. Sanner, D. S. Goodsell, A. J. Olson, *Nat. Prot.*,2016, **11**, 905–919.
[18] Maestro-Desmond Interoperability Tools, version 4.2, Schrçdinger, New York, NY, 2015
[19] Desmond Molecular Dynamics System, version 4.2, D. E. Shaw Research, New York, NY, 2015.
[20] K. J. Bowers, E. Chow, H. Xu, R. O. Dror, M. P. Eastwood, B. A. Gregersen, J. L. Klepeis, I. Kolossvary, M. A. Moraes, F. D. Sacerdoti, J. K. Salmon, Y. Shan , D. E. Shaw, in Proceedings of the 2006 ACM/IEEE Conference on Supercomputing, 2006.
[21] W. L. Jorgensen, J. Chandrasekhar, J. D. Madura, R. W. Impey, M. L. Klein, *J. Chem. Phys.*, 1983, **79**, 926-935.

[22] J. L. Banks, H. S. Beard, Y. Cao, A. E. Cho, W. Damm, R. Farid, A. K. Felts, T. A. Halgren, D. T. Mainz, J. R. Maple, R. Murphy, D. M. Philipp, M. P. Repasky, L. Y. Zhang, B. J. Berne, R. A. Friesner, E .Gallicchio, R. M. Levy, *J. Comp. Chem.*, 2005, **26**, 1752.
